# Genomic, morphological, and biochemical analyses of a multi-metal resistant but multi-drug susceptible strain of *Bordetella petrii* from hospital soil

**DOI:** 10.1038/s41598-022-12435-7

**Published:** 2022-05-19

**Authors:** Urmi Halder, Raju Biswas, Ashutosh Kabiraj, Rajendar Deora, Moitri Let, Rajendra Kr Roy, Annapurna Chitikineni, Krishnendu Majhi, Shrabana Sarkar, Bhramar Dutta, Anubhab Laha, Arunava Datta, Dibyendu Khan, Rajeev K. Varshney, Dipnarayan Saha, Saswati Chattopadhyay, Rajib Bandopadhyay

**Affiliations:** 1 Microbiology Section, Department of Botany, The University of Burdwan, Burdwan, West Bengal 713104 India; 2 Department of Microbial Infection and Immunity, The Ohio State University, Columbus, OH 43210 USA; 3 Center of Excellence in Genomics and Systems Biology, International Crops Research Institute for the Semi-Arid Tropics (ICRISAT), Hyderabad, 502324 India; 4 State Agricultural Biotechnology Centre, Centre for Crop and Food Innovation, Murdoch University, Murdoch, 6500 Australia; 5 Biotechnology Unit, Division of Crop Improvement, ICAR-Central Research Institute for Jute and Allied Fibres, Barrackpore, West Bengal 700121 India; 6 Department of Microbiology, Burdwan Medical College, Burdwan, West Bengal 713104 India

**Keywords:** Environmental microbiology, Microbial genetics, Bacterial genes

## Abstract

Contamination of soil by antibiotics and heavy metals originating from hospital facilities has emerged as a major cause for the development of resistant microbes. We collected soil samples surrounding a hospital effluent and measured the resistance of bacterial isolates against multiple antibiotics and heavy metals. One strain BMCSI 3 was found to be sensitive to all tested antibiotics. However, it was resistant to many heavy metals and metalloids like cadmium, chromium, copper, mercury, arsenic, and others. This strain was motile and potentially spore-forming. Whole-genome shotgun assembly of BMCSI 3 produced 4.95 Mb genome with 4,638 protein-coding genes. The taxonomic and phylogenetic analysis revealed it, to be a *Bordetella petrii* strain. Multiple genomic islands carrying mobile genetic elements; coding for heavy metal resistant genes, response regulators or transcription factors, transporters, and multi-drug efflux pumps were identified from the genome. A comparative genomic analysis of BMCSI 3 with annotated genomes of other free-living *B. petrii* revealed the presence of multiple transposable elements and several genes involved in stress response and metabolism. This study provides insights into how genomic reorganization and plasticity results in evolution of heavy metals resistance by acquiring genes from its natural environment.

## Introduction

Development of drug and heavy metal resistance in bacteria, particularly resulting in reduction of antibiotic efficacy has intensified in last few decades. Hospital activities like byproducts discharges are generally a big source of antibiotics and heavy metals^1–4^. Health care facilities from different geographical locations are getting contaminated with ~ 1.5–310 g/day due to the uncontrolled and extensive use of drugs. Over the past few years, in different countries hospital effluents have resulted in the discharge of multiple antibiotics; erythromycin (13–7545 ng/L), azithromycin (113–7351 ng/L), clarithromycin (10–14,000 ng/L), spiramycin (40–2200 ng/L), josamycin (12–15 ng/L), roxithromycin (140–2189 ng/L), clindamycin (31–1465 ng/L), lincomycin (7–48,400 ng/L), ofloxacin (662–37,000 ng/L), ciprofloxacin (11–101,000 ng/L), lomefloxacin (313–1162 ng/L), levofloxacin (150–750 ng/L), enoxacin (450–480 ng/L), norfloxacin (100–44,000 ng/L), sulfadiazine (19.2–6640 ng/L), and sulfamethoxazole (16.1–37,300 ng/L)^1^. Furthermore, hospitals are also a major source of toxic metal and metalloids contamination like, cyanide, gadolinium (2–320 μg/L), platinum (0.02–300 μg/L), mercury (0.05–6 μg/L), silver (120–500 μg/L), copper (60–250 μg/L), nickel (8–80 μg/L), lead (4–20 μg/L), zinc (80–700 μg/L) and arsenic (0.7–12 μg/L)^1^.

Due to these exposures in the natural environment, clinically-relevant bacteria evolve complex protective metabolic activities that help them to withstand the deleterious effects of antibiotics, resulting in a negative impact on public health^5–8^. Similarly, exposure to toxic metals like cadmium, arsenic, chromium, lead, mercury, etc. leads to acquisition of metal resistant genes and efflux pumps to minimize toxicity. For example, CadA and ArsAB proteins are involved in ATP-dependent efflux of cadmium and arsenite ions, respectively from bacterial cells^9^. In most cases, cytosolic, membrane bound or periplasmic enzymes like arsenate reductase, chromate reductase, mercuric reductase, arsenite oxidase, etc. are involved in metal detoxification^10–12^. In the presence of arsenite methyltransferase (ArsM), arsenite is methylated into different forms allowing cells to survive under arsenic like toxic material^13^. A primary focus of ongoing research is to determine if any connection exists between antibiotics and heavy metal resistance in microbes. Many studies suggest that there is a link between multidrug and heavy metal resistance in bacteria^14,15^. Mutations within the genome or the existence of resistant genes within the same mobile genetic element (MGE) are known to mediate cross-resistance to both antibiotics and heavy metals^16^. However, natural selection leads to the evolution in acquisition of resistance development in a random way that both antibiotic resistance and heavy metal tolerance are sometimes not linked^17^. One such interesting phenomenon has been highlighted in this study, where we isolated a bacterium *B. petrii* BMCSI 3 from effluent adjacent soil sample of the Medical College and Hospital located in Burdwan, West Bengal, India. Although, this strain was tolerant to multiple heavy metals, it was susceptible to several groups of antibiotics.

*Bordetella petrii* is a free-living, widely distributed environmental species. Earlier, a *B*. *petrii* strain was isolated from a cystic fibrosis patient,  shown to be resistant to a variety of antibiotics^18^. In this investigation, we have sequenced the genome, characterized different genomic regions, and identified morphological features. Furthermore, we have pointed out the major genomic features of this strain with other *Bordetella* species and strains of *B. petrii*.

## Results and discussion

### Isolation, multi-drug and multi-metal tolerance of strain BMCSI 3 from soil

**Figure 1:**
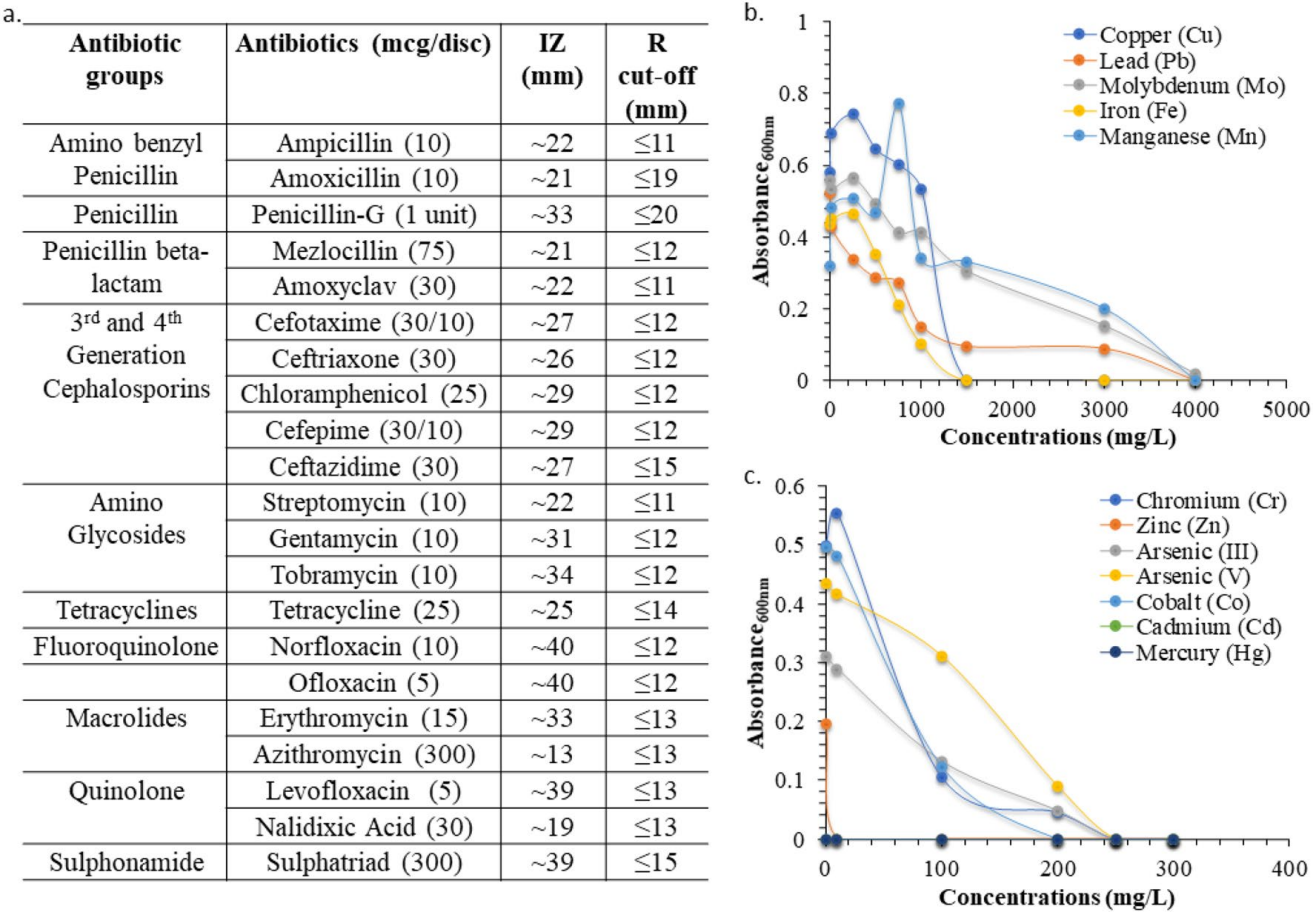
Multi-drug and multi-metal tolerance capacity of BMCSI 3. Antibiotic sensitivity against different antimicrobial agents (*IZ* inhibition zone; *R* resistance) (**a**); metal tolerance capacity against different heavy metals (**b**,**c**).

Four individual bacterial colonies grew on Luria–Bertani Agar plate of which one was designated as BMCSI 3. First, we tested its sensitivity against multiple antibiotics. It was found to be sensitive to all the tested antibiotics including Amino benzyl penicillin, Penicillin, Penicillin beta-lactam, 3^rd^ and 4^th^ generation Cephalosporins, Amino Glycosides, Tetracyclines, Fluoroquinolone, Macrolides, Quinolone, and Sulphonamide group of antibiotics (Fig. 1a). Using X-ray fluorescence spectroscopy (XRF), we determined that the soil was contaminated with multiple toxic metals like Titanium (Ti), Iron (Fe), Copper (Cu), Zinc (Zn), Molybdenum (Mo), Cadmium (Cd), and Lead (Pb). The presence of Potassium (K) and Calcium (Ca) were also detected from XRF spectroscopy. Therefore, we tested the growth of BMCSI 3 in the presence of toxic heavy metals. BMCSI 3 grew in the presence of very high concentrations of salts of Lead, Molybdenum, Manganese (⁓3000 mg/L), Copper (⁓1000 mg/L), and Iron (⁓750 mg/L) (Fig. 1b). In comparison to these heavy metals, BMCSI 3 tolerated lower concentrations (⁓10 mg/L) of Chromium, Cobalt, Arsenic (III and V) salts and did not grow in the presence of Zinc, Cadmium and Mercury (Fig. 1c). However, by analyzing these results, in one way, BMCSI 3 is susceptible to antibiotics, other way, it is highly resistant to multiple metals.

### Whole-genome sequences and phylogenomic inference

The draft genome of BMCSI 3 was assembled using the three genome assemblers, SPAdes, Velvet, and ABySS; the latter two were based on the best suited K-mer statistics. For instance, the best assembly statistics were obtained with K-mer length of 99 in Velvet and k-mer length of 96 in ABySS. The contigs produced by the SPAdes, Velvet, and ABySS programs are 66, 14, and 114, respectively. The comparative assembly statistics using the QUAST software revealed that the assemblies consisted of approximately 4.9 Mb genome with the highest N50 length of 3.8 Mb was found with Velvet assembly, which was finally processed for further analysis. The finalized draft assemblies of BMCSI 3 were 99.53% complete without any contaminations (Supp. file S1).

**Figure 2:**
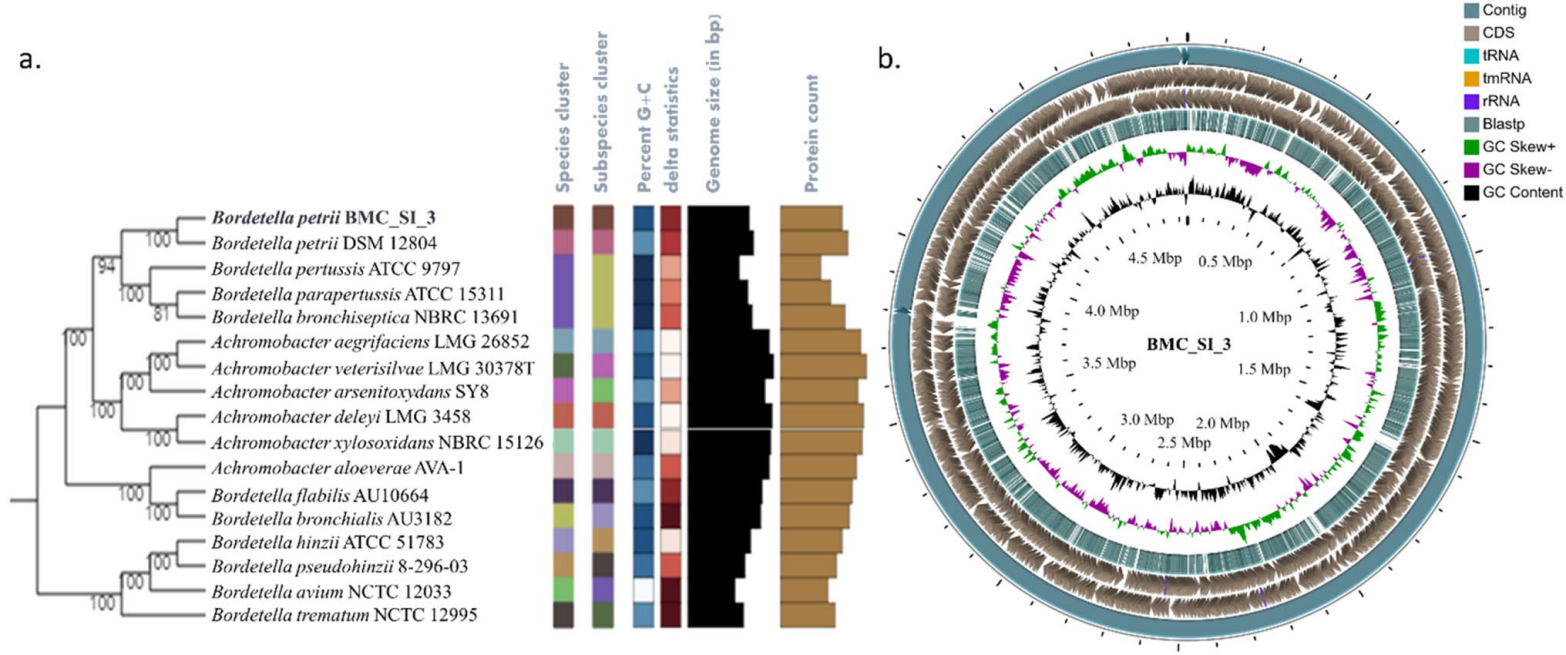
TYGS result for the BMCSI 3 genome (**a**). Circular representation of BMCSI 3 genome features as per the CG viewer server (**b**).

Whole-genome sequence of BMCSI 3 was analyzed to predict its taxonomic status through different web tools. As per the predictions made by MiGA, StrainSeeker, and TYGS webservers, the closest relative determined was *B. petrii* DSM 12804^ T^. The tree inferred with FastME 2.1.6.1^19^ from GBDP (Genome BLAST Distance Phylogeny) distances calculated from genome sequences. The branch lengths were scaled in terms of GBDP distance formula d_5_. The numbers above branches are GBDP pseudo-bootstrap support values > 60% from 100 replications, with average branch support of 97.9%. The tree was rooted at the midpoint^20^ (Fig. 2a). The taxonomic classification was thus resolved to *B. petrii* under the family Alcaligenaceae, order Burkholderiales, and phylum Proteobacteria. Different features of *B. petrii* BMCSI 3 genome were analyzed including the coding sequence of genes and GC content distributed across the scaffolds. From outermost to centre, distribution of scaffolds (Ring-1); protein-coding genes (CDS) in forward and reverse strand (Ring-2 and 3); Blastp hit with a reference *B*. *petrii* DSM 12804^ T^ genome (Ring-4); GC skew plot with a value on above average and below average (Ring-5); and plots of GC content (Ring-6) (Fig. 2b).

### Genome features

BMCSI 3 contains a total of 4,708 genes of which 4,638 were protein-coding genes (CDS) and 41 were pseudogenes. The number of genes that encode tRNAs, rRNAs, and ncRNAs were found to be 54, 12, and 4, respectively. Annotation by RAST resulted in 29% (1337) of the protein-coding genes being covered under the 324 subsystems. The metabolism of amino acids and derivatives occupied the largest number of genes (434) followed by carbohydrate metabolism (226), and protein metabolism (206). A significant number of genes (54) were related to virulence, disease, and defense.

Consistent with its tolerance to multiple metals, BMCSI 3 harbored a large number of genes with potential functions in multidrug efflux and heavy metal resistance like MATE family efflux transporter; multidrug efflux SMR transporter; chromate efflux transporter; multidrug efflux RND transporter permease subunit and periplasmic adaptor subunit; outer membrane efflux transporter subunit; fluoride efflux transporter CrcB; Bcr/CflA family efflux MFS transporter; DHA2 family efflux MFS transporter permease subunit; nickel/cobalt efflux transporter RcnA; ACR3 family arsenite efflux transporter; arsenical efflux pump membrane protein ArsB; Co^2+^/Mg^2+^ efflux protein ApaG; HlyD family efflux transporter periplasmic adaptor subunit; quaternary ammonium compound efflux SMR transporter SugE and QacE; CusA/CzcA family heavy metal efflux RND transporter; MdtB/MuxB and MdtA/MuxA family multidrug efflux RND transporter periplasmic adaptor subunit; AdeC/AdeK/OprM family multidrug efflux complex outer membrane factor basically involve in effluxing, detoxifying or scavenging excess metal ions^21^ was identified in the genome of metal-loving BMCSI 3. Presence of proteins like heavy metal sensor, response regulator and efflux system could also influence heavy metal resistant^22,23^. The presence of two-component response regulators and multiple genes with potential function in heavy metal resistance resulted in successful adaptation and survival of this strain in an environment that is heavily contaminated with toxic metals^24^.

### Morphology and biochemical features

**Figure 3:**
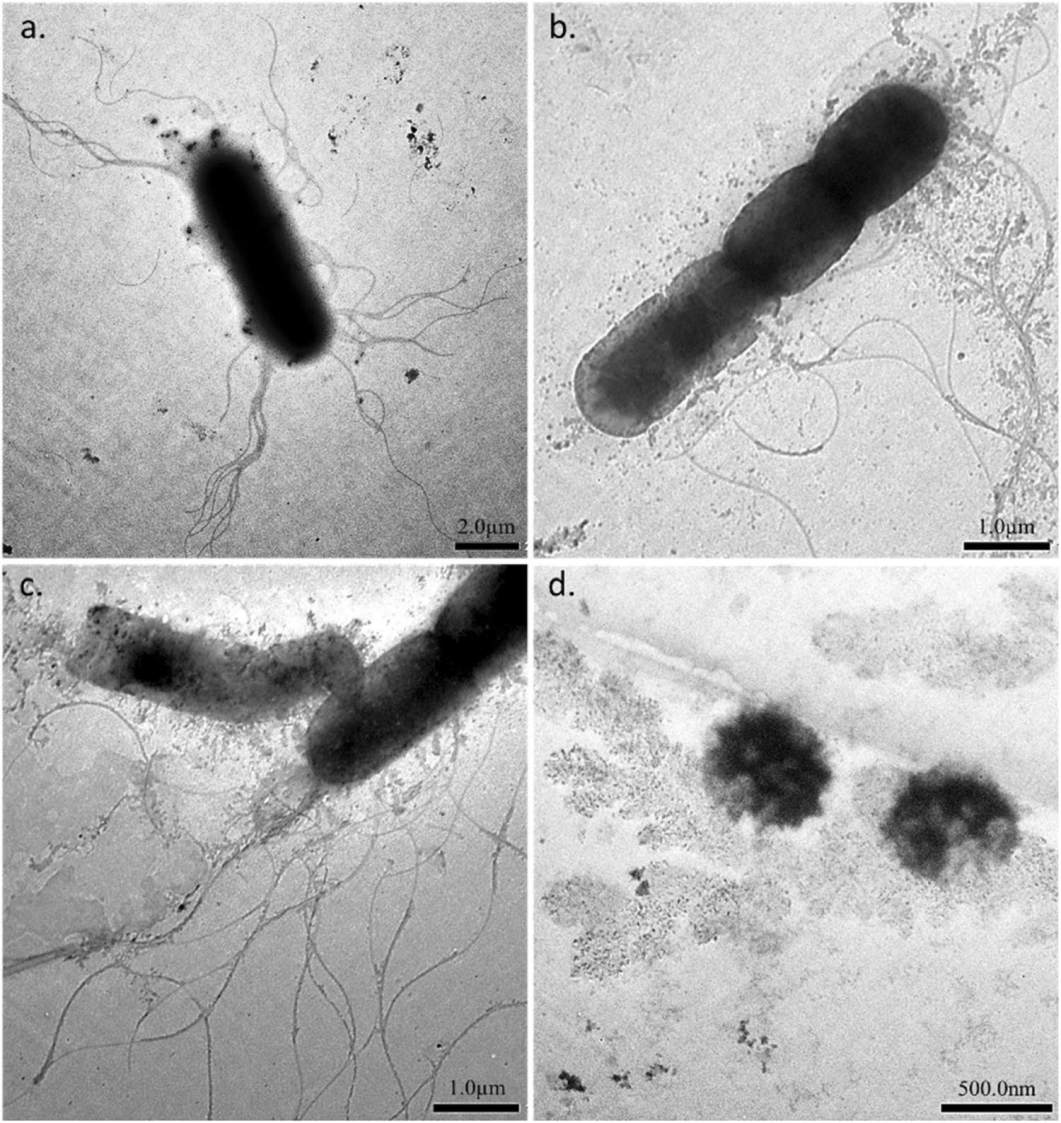
Transmission Electron micrographs showing mature vegetative cells with peritrichous flagellation (**a**); different stages of cell division (**b**,**c**); and free endospores (**d**) of BMCSI 3.

BMCSI 3 is a Gram-negative, rod-shaped bacterium. This strain tested positive for catalase, amylase, lipase and utilized citrate as carbon source. A total of 14 protein-coding genes related to flagella under the category of motility and chemotaxis were identified including flagellar biosynthesis proteins like FlhA, FlhB, FliR, FlhF; motor switch protein FliM, FliN; motor rotation protein MotA, MotB; basal-body rod modification protein FlgD; L-ring protein FlgH. Other chemotaxis related proteins, like CheR, CheW, CheB, CheA, response regulator CheY including a type IV pili methyl-accepting chemotaxis transducer N-terminal domain-containing protein that usually mediates chemotactic response were also found to be present^25^. Generally, *Bordetella* species sense environmental cues and control transcription of virulence genes by the activity of BvgAS (*Bordetella* virulence gene) system. When BvgAS is inactive, *Bordetella* produce functional flagella and thus are motile. The genome of BMCSI 3 lacks genes homologous to *bvgA* and *bvgS*^26^. Based on these findings, we hypothesized that BMCSI 3 will be flagellated. Consistent with this hypothesis, transmission electron micrographs revealed peritrichous flagellation and ⁓8–10 µm elongated mature vegetative cells (Fig. 3a–c).

A forespore-like formation and cell outburst was observed within the mature vegetative cell of BMCSI 3 (Fig. 3c). Free endospore-like structures (Fig. 3d) were also observed. To date, the phenotype of sporulation has not been reported in *Bordetella*. Additionally, micrographs clarifying the cell morphology are not available for *B. petrii*^27^. In the draft genome of BMCSI 3, *spoVC* that encodes peptidyl-tRNA hydrolase and is essential for vegetative growth was present along with spore maturation proteins^28^. However, genes homologous to sporulation-specific sigma factors of *Bacillus* species were absent^29–31^.

### Genomic islands

**Figure 4:**
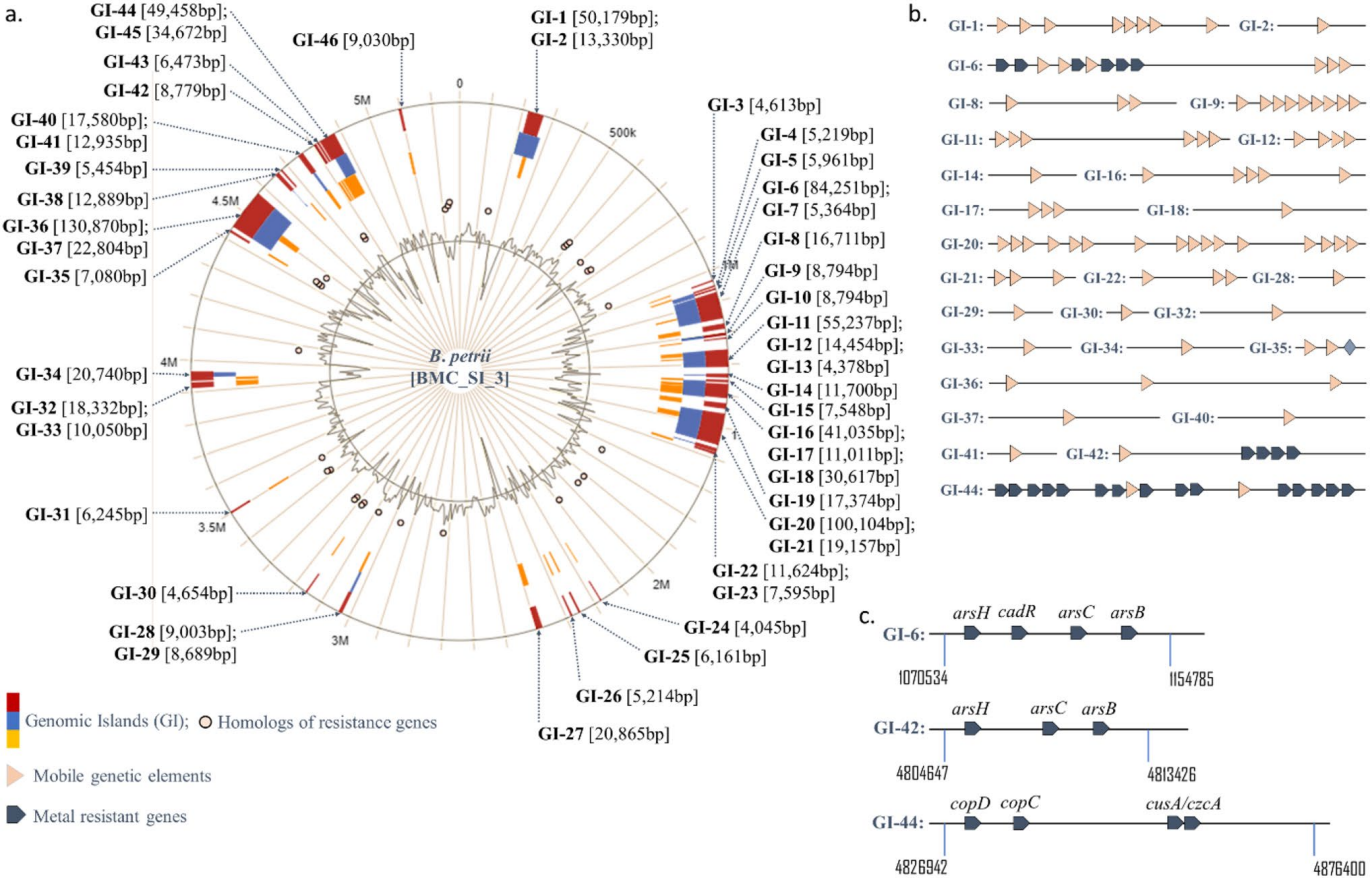
Genomic Islands (GIs) of BMCSI 3 genome (**a**); presence of mobile genetic elements (**b**) and metal resistant genes (**c**) were marked on the GIs (Developed from Supp. file S2).

A total of 46 Genomic Islands (GIs) of BMCSI 3 were identified which covered about 20% of the genome. Approximately, 10% (88,743 bp) of the genomic islands were comprised of mobile genetic elements in the form of integrative and conjugative elements like the most abundant IS3 family including ISL3, IS5, IS21, IS66, IS91, IS110, IS360, IS1066, and IS1663 families (Fig. 4). Consistent with previously published report, the repetitive element IS481, which is a frequent target of diagnosis of other *Bordetella* species was not found in BMCSI 3^27,32^. Most of the putative laterally acquired GIs found in BMCSI 3 harbored heavy metal resistant genes, multiple response regulators and other transcription factors, transporters, and multi-drug efflux pumps (Supp. file S2). GI-1 contains a TniQ family protein having a role in transposition of the mercury-resistance transposon Tn5053^33^. GI-1 and 2 harbours genes having homology to the ZorAB system, which is involved in the opening of the proton pump leading to the membrane depolarization upon phage infection and ensuring abortive transduction^34^. This may lower the chances of acquiring new genes, especially antibiotic resistance genes into the bacterial genome. Consistent with this, BMCSI 3, has genes that are usually associated with antibiotic resistance, such as small multidrug resistance (SMR) and resistance-nodulation-division (RND) antibiotic efflux pump^35^. Interestingly, no such cross-resistance link between heavy metals resistance and antibiotic resistance was found. Eventually, almost all the SMR and RND groups of efflux pump systems are likely to involve metals resistance, instead of antibiotic resistance during different environmental stresses (Fig. 1). HlyD, an important component of CusCFA efflux membrane fusion protein is present in multiple GIs. This type of resistance-nodulation-division group (RND) has evolved to secrete toxic metallic ions outside the periplasmic space^36^. MFS transporters involved in both efflux and influx of various substances including drug efflux from the cell are also present in the genome^37^. Another major uncharacterized transporter belonging to the DMT family of transporters is also integrated into the genome. GI-6 and GI-42 harboured arsenical resistance protein-coding gene *arsH*, Cd(II)/Pb(II)-responsive transcriptional regulator protein-coding gene *cadR*, arsenate reductase protein-coding gene *arsC*, and ACR3 family arsenite efflux transporter related gene *arsB*. GI-44 harbors multicopper oxidase domain-containing protein, copper-translocating P-type ATPase, efflux RND transporter periplasmic adaptor and permease subunit, copper-binding protein, metal-binding protein, copper homeostasis membrane and periplasmic binding protein, copper resistance protein B, copper resistance system multicopper oxidase, heavy metal sensor histidine kinase, heavy metal response regulator transcription factor, CusA/CzcA family heavy metal efflux RND transporter, and periplasmic adaptor subunit. GI-33 acquired a universal stress protein that may regulate a broad range of cellular responses against biotic and abiotic stress^38^. Additionally, GI-44 harboured the gene encoding for the copper-translocating P-type ATPase whose primary function is to transport copper across the biological membrane^39^. Copper homeostasis membrane protein CopD, periplasmic protein CopC, copper resistant protein B, multicopper oxidase are also present in the same genomic island which may promote tolerance in the presence of copper. A MATE family efflux transporter CusA/CzcA family heavy metal efflux is present in GI-44. Additionally, genes encoding heavy metal sensor histidine kinase and heavy metal response regulator transcription factor are integrated within the GI-44 at multiple sites.

### Genome comparison

**Figure 5:**
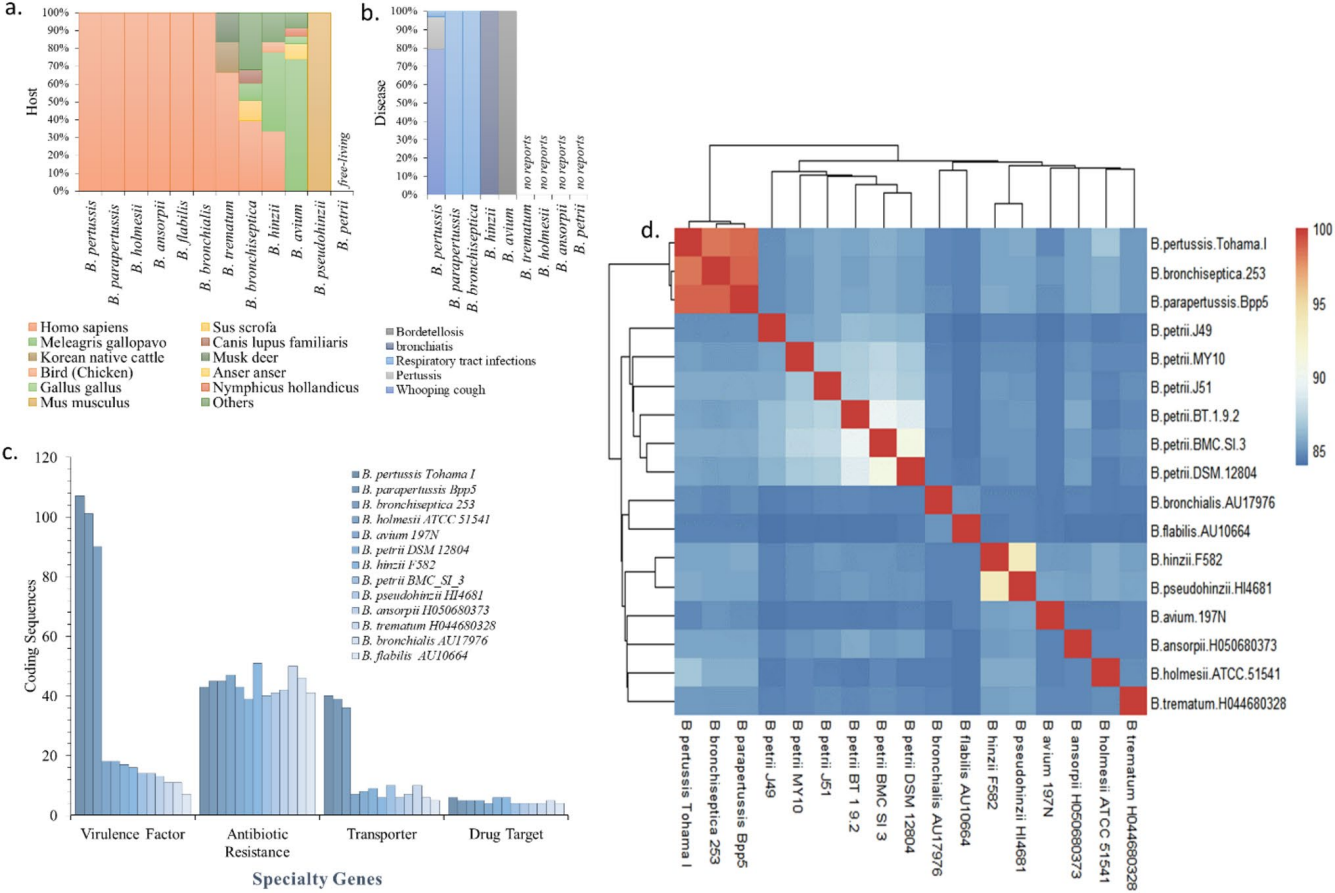
Genome information of the genus *Bordetella*. Host specification (**a**); disease causing abilities (**b**); specialty genes (**c**) (developed from Supp. file S3), and ANI heat map cluster of all representative species including strains of *B. petrii* (**d**).

Bacteria belonging to the genus *Bordetella* are gram-negative coccobacilli of the phylum proteobacteria. Most species in this genus are capable of causing a wide spectrum of pulmonary and bronchial diseases, in humans, animals, and birds^40,41^. To date, of the described nine species, *B. pertussis* and *B. parapertussis* cause whooping cough in humans^42^, while *B. bronchiseptica* infects primarily animals, and rarely humans^43^. Despite the fact that, evolutionary trend analysis demonstrated that the ancestors of this genus were of environmental origin, a significant loss of metabolic genes and acquisition or retention of virulence factors within the genome has driven this particular genus to emerge as an opportunistic pathogen^44,45^. Comparative analysis within the representative strains from *Bordetella* spp. revealed the abundance of transposable elements and presence of stress response genes within genomes of free-living *B*. *petrii*. Virulence factor and transporter-related protein numbers were significantly high in *B*. *pertussis*, *B*. *parapertussis*, *B*. *bronchiseptica* species. In comparison, *B*. *petrii* contains a relatively lower number of virulence genes; antibiotic resistance and drug target-related protein numbers were almost equal among all the selected species. Genome similarities were represented and clusters were determined within the species of *Bordetella* based on Average Nucleotide Identity (ANI) analysis, where 95–96% was the species distinction cut-off value. Likewise, species of *B*. *pertussis*, *B*. *parapertussis*, *B*. *bronchiseptica*, strains of *B. petrii* were not correctly grouped with that threshold value. ANI value < 90% is usually evidenced by taxonomic differences^46^ (Fig. 5).

**Figure 6:**
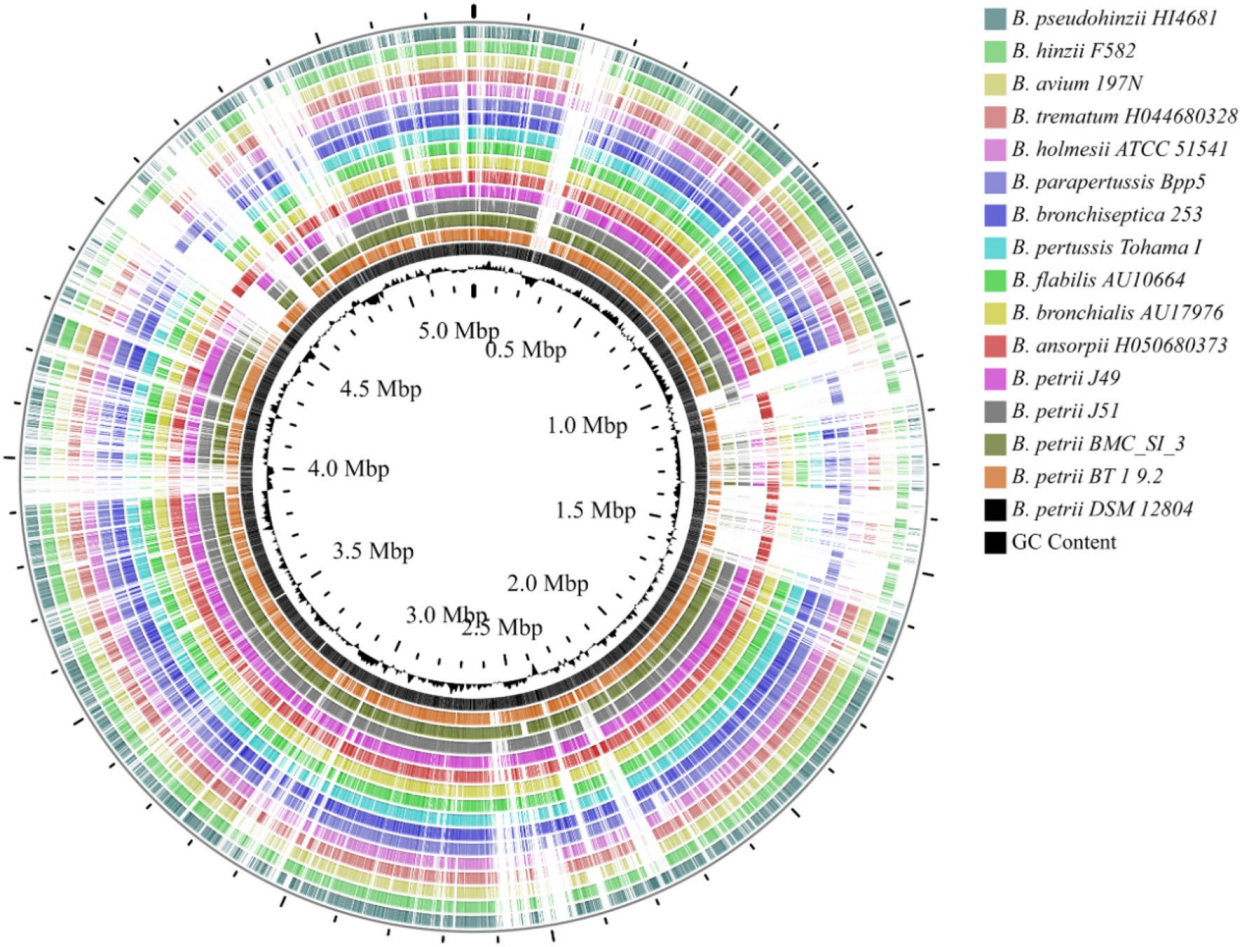
Blast analysis and circular genome comparison of the species of *Bordetella*.

All the sixteen genomes of different species of *Bordetella* were compared along with the available strains of *B*. *petrii* using *B*. *petrii* DSM 12804 as a reference. From outermost to centre (Fig. 6), *B. pseudohinzii* (Ring-1), *B. hinzii* (Ring-2), *B. avium* (Ring-3), *B. trematum* (Ring-4), *B. holmesii* (Ring-5), *B. parapertussis* (Ring-6), *B. bronchiseptica* (Ring-7), *B*. *pertussis* (Ring-8), *B. flabilis* (Ring-9), *B. bronchialis* (Ring-10), *B. ansorpii* (Ring-11), *B. petrii* J49 (Ring-12), *B. petrii* J51 (Ring-13), *B. petrii* BMCSI 3 (Ring-14), *B. petrii* BT 1 9.2 (Ring-15), *B. petrii* DSM 12804 (Ring-16) and plots of GC content (Ring-17). Transposable elements were most abundant within the genomes of free-living *B*. *petrii*. A lot of metabolism related genes like putative monooxygenase coding gene, *alsT*, *yhiB*, *menH* (2-succinyl-6-hydroxy-2,4-cyclohexadiene-1-carboxylate synthase)^47^, *arcD* (arginine/ornithine antiporter), *areA* (Nitrogen regulatory proteins which are GATA type transcription factors)^48^, *hdfR* (encodes a LysR family protein)^49^, *cysIJ*, *hemN* (oxygen-independent coproporphyrinogen-III oxidases)^50^, *nrdD* (Anaerobic ribonucleoside-triphosphate reductase activating protein)^51^, *yhbU*, *ptlB*, *nimT* (2-nitroimidazole transporter and similar proteins of the Major Facilitator Superfamily of transporters)^52^, *tmoT*, NADH dehydrogenase like protein coding gene, *sasA* (Adaptive-response sensory-kinase)^53^, *yheG* putative multidrug efflux transporter coding gene, *norB*, *qoxC* (subunit III of the aa3-type quinone oxidase)^54^, *nirBQST*, *mftC*, *eysG*, *ccmH* (Cytochrome C biogenesis protein)^55^, *zupT* (Zinc transporter)^56^, *cusAB*, *dsbD*, *cnrA* (membrane-bound protein complex catalyzing an energy-dependent efflux of Ni^2+^ and Co^2+^)^57^, *hcaR*, *gcd*, *iolG*, *wbpl*, *mshA*, *pglK* (six-transmembrane helical domain of the ABC transporter)^58^ were only found within the genome of *B*. *petrii*. Interestingly, virulence regulon transcriptional activator related gene *virB* was only found within the genomes of *B*. *petrii* (Supp. file S4).

Next, we carried out a comparative genome analysis of *B*. *petrii*. To date, six soil-born strains of *B. petrii* whose genome has been sequenced (Table 1) of which only the Type Strain *B. petrii* DSM 12804 with a complete genome was reported from a river sediment enriched dechlorinating bioreactor^27^. Other strains viz.,* B. petrii* J49 and *B. petrii* J51 were reported from aquatic soil and strain BT 1 9.2 was reported from contaminated soil. Another strain MY 10 was also reported from soil^59^. Genome sizes of these other strains ranged from 4.21 to 6.10 Mb, with an average GC% ranging from 65.4 to 67.3. The 4.95 Mb genome of BMCSI 3 was smaller than three other *B. petrii* strains and the overall GC content of 67.3% was the second highest among the six sequenced strains. The different categories of the genes present in the five genomes are shown in Table 1. Both BMCSI 3 and BT 1 9.2 isolated from contaminated soil harboured a significantly large number of heavy metal response and efflux transporter related proteins, but a smaller number of ABC transporter, virulence, motility and chemotaxis related proteins compare to the other four strains. Only the strain BT 1 9.2 is devoid of any type IV pili methyl-accepting chemotaxis transducer protein related to chemotaxis. Transmembrane signaling receptor PAS domain-containing methyl-accepting chemotaxis protein was found within strain DSM 12804, J49 and BMCSI 3. Two SOS response-associated peptidase family proteins were found within the chromosome of strain DSM 12804 but as a sign of rapid genome adaptation, this protein was acquired within the island of strain BMCSI 3 genome. Only three strains (J49, J51, and BT 1 9.2) of *B*. *petrii* harbour genes involved in sulfur metabolism and only two strains BMCSI 3 and DSM 12804 harbored the Type IV secretion system, multi-subunit secretion apparatus involved in secretion of macromolecules across the membranes. The highest numbers of tRNA were found within BMCSI 3 followed by DSM 12804. The decoding of mRNA into protein is governed by tRNA. Since the structural diversity of tRNA most likely co-evolved with their processing RNA splicing endonuclease^60^, it may be significant in the evolution of BMCSI 3.

**Table 1:** NCBI PGAP annotated genome features comparison between all available strains of *B. petrii*.

	*B. petrii* DSM 12804^T^	*B. petrii* BMCSI 3	*B. petrii* BT 1 9.2	*B. petrii* MY 10	*B. petrii* J49	*B. petrii* J51
Isolation source	River sediment	Hospital soil	Contaminated soil	Soil	Aquatic soil	Aquatic soil
Assembly	GCA_000067205.1	GCA_017356245.1	GCA_017745595.1	GCA_020991325.1	GCA_000518845.1	GCA_000518965.1
Size	5.29	4.95	6.10	4.95	4.21	5.04
GC%	65.50	67.30	65.90	65.70	65.40	68.50
Genes (total)	5105	4708	5686	5055	3990	4870
CDSs (total)	5041	4638	5630	4991	3899	4779
rRNAs	9	12	5	11	5	8
tRNAs	51	54	47	49	47	47
ncRNAs	4	4	4	4	4	4
Pseudo genes (total)	132	41	58	77	35	32
Motility and chemotaxis related protein	17	9	8	13	11	13
Methyl-accepting chemotaxis proteins	19	13	12	17	13	16
Heavy metal related proteins	18	23	26	16	10	18
Transporter	656	671	760	767	597	771
ABC transporter	315	225	294	340	319	386
Efflux transporter related proteins	42	53	51	36	29	41
Virulence related proteins	2	1	1	2	2	3
Stress-related proteins	40	38	43	35	30	43

**Figure 7:**
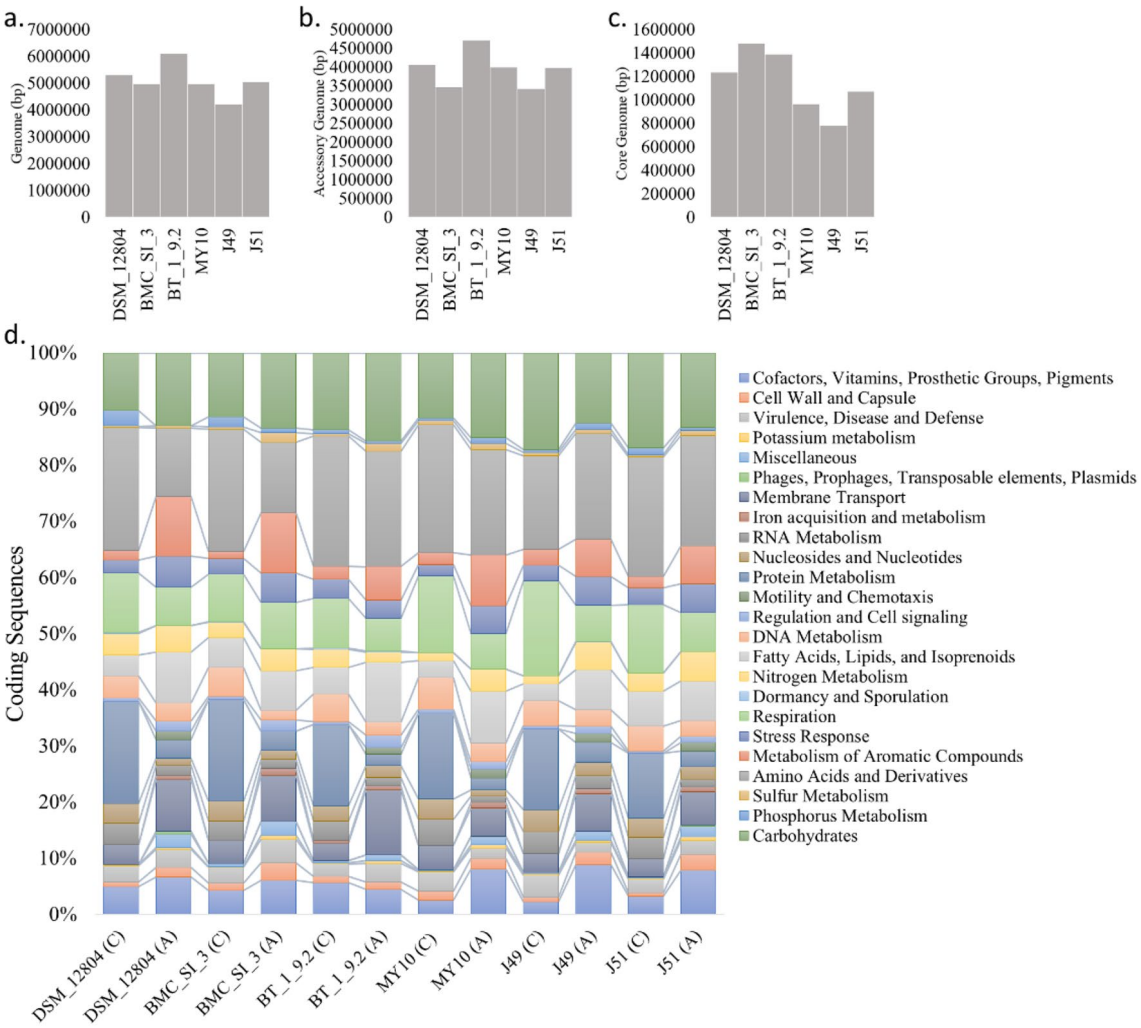
Pangenome analysis strains representing strain-specific genome size (**a**), accessory genome size (**b**), core genome size (**c**), and functional annotation of *Bordetella petrii*.

Pangenome analysis of six genomes of *B. petrii* represents 52,87,950 bp to be pangenome size including 10,69,661 bp core genes, 12,34,707 bp accessory genes and 29,83,582 bp strain-specific genes shared by at least 2 strains. So, the genome diversity of *B. petrii* represents an open pangenome model. However, functional analysis of strain-specific accessory and core genome suggests a usual core metabolism by those strains (Fig. 7).

## Conclusion

Bacteria belonging to the genus *Bordetella* can cause a wide range of pulmonary and bronchial infections in humans, animals, and birds. However, *B*. *petrii*, a member of this genus has a wide spectrum of occurrence within the natural habitat. In this study, we report that a soil-inhabiting *B. petrii* strain isolated from soil sample adjacent to a hospital’s effluent can tolerate toxic metalloids by acquiring different metal tolerant genes. Surprisingly, despite being of hospital origin, this strain was susceptible to multiple antibiotics. BMCSI 3 featured putative multidrug efflux transporters like MATE, MSF, RND, DMT, as well as ABC family proteins that are possibly involved in the effluxing of excess metal ions but not the drug molecules. The genomic islands represented a significant number of integrative and conjugative transposable elements. Moreover, genomic islands carried genes that encode metal efflux transporter (ACR3), copper resistant operon (Cop), and mercury-resistance transporter (mer) proteins that may result in metal resistance. Collectively, our results suggest that resistance to antibiotics and tolerance to metals are not always linked. A detailed phenotypic investigation of *B. petrii* is needed to understand the mechanisms through which microbes become resistant to heavy metals.

## Methodology

### Sampling and isolation

Soil samples were aseptically collected from effluent adjacent dump backyard area of the Emergency Unit, Burdwan Medical College and Hospital, Burdwan, West Bengal, India (23.2489° N, 87.8536° E). Qualitative elemental analysis of the soil sample was analyzed using X-ray fluorescence spectroscopy (Artax, Bruker). For isolation of bacterial species, 1.0 g of soil sample was diluted gradually up to 10^−4^ in sterile saline Milli-Q (0.1% NaCl, HiMedia) and 100 μL of each dilution was plated onto Luria Bertani Agar medium (HiMedia) and incubated at 30 °C for 24 h^61^.

### Screening of multi-drug and multi-metal tolerance

All individual bacterial colony forming units were subjected to the preliminary antibiotic sensitivity assay against different groups of antibiotics, viz., ampicillin (10 mcg), amoxicillin (10 mcg), penicillin G (10 IU), mezlocillin (75 mcg), amoxiclav (30 mcg), cefotaxime (30/10 mcg), ceftriaxone (390 mcg), chloramphenicol (30 mcg), cefepime (30/10 mcg), ceftazidime (30 mcg), streptomycin (10 mcg), gentamicin (10 mcg), tobramycin (10 mcg), tetracycline (30 mcg), norfloxacin (10 mcg), ofloxacin (5 mcg), erythromycin (15 mcg), azithromycin (15 mcg), levofloxacin (5 mcg), nalidixic acid (10 mcg), sulphatriad (300 mcg) (Himedia). Freshly prepared bacterial culture was spread onto the culture plates containing Mueller Hinton Agar media (HiMedia) and was allowed to incubate overnight at 30 °C^61^. This experiment was performed in triplicate and inhibition zones (IZ) were measured. The antibiotic concentrations were selected and the sensitivity was determined on the basis of clinical breakpoints provided by the Clinical and Laboratory Standards Institute and European Committee on Antimicrobial Susceptibility Testing^62,63^. Multi-metal resistant capability was also tested in triplicate against 10 different heavy metal salts (Fe, Cu, Cr, Pb, Cd, Hg, Mn, Mo, Co, Zn) and 1 metalloid (As)^64^. Nutrient Broth (HiMedia) media were prepared and supplemented with 200 μg/L and (10, 100, 200, 250, 300, 500, 750, 1000, 1500, 3000, and 4000) mg/L concentrations of FeCl_3_, 6H_2_O; CuSO_4_, 5H_2_O; CrO_3_; PbCl_2_; CdCl_2_; HgCl_2_; MnCl_2_, 4H_2_O; Na_2_MoO_4_; CoCl_2_, 6H_2_O; ZnSO_4_, 7H_2_O along with Na_2_AsHO_4_, 7H_2_O and NaAsO_2_ (HiMedia; Merck). A freshly prepared 10 µL of bacterial culture was inoculated on each prepared broth and were allowed to incubate overnight at 30 °C, 130 rpm. Cell density was measured by the absorbance value taken at 600 nm using a Spectrophotometer (Lasany LI-721). Based on the multi-drug and multi-metal tolerance capability, strain BMCSI 3 was selected for this study.

### Morphology and physicochemical study

Cell morphology of BMCSI 3 was studied by performing Transmission electron microscopy. Cell culture was drop cast onto a carbon-coated Cu grid (Sigma Aldrich), air-dried, and images were acquired under JEM 1400 plus, JEOL Transmission Electron Microscope (120 keV).

Different physicochemical properties of strain BMCSI 3 like gram characteristics, extracellular enzyme activities (catalase, amylase, and protease), substrate hydrolysis (tributarin, gelatin, starch), and substrate utilization (lysine, citrate) were also performed^65^.

### Genome sequencing

Cells of BMCSI 3 were cultured on Luria Bertani Agar media (HiMedia) at 30 °C, overnight and genomic DNA was extracted using the Quick-DNA Fungal/Bacterial Miniprep Kit (Zymo Research, USA). Final DNA concentration and purity were obtained using NanoDrop 1000 (Thermo Scientific, USA) and the genomic DNA integrity was checked on 1.5% agarose gel electrophoresis^66^. Approximately, 1 µg of genomic DNA was used to construct sequencing libraries by using the TruSeq™ DNA PCR-Free library preparation kit (Ilumina, Inc., USA). Before library preparation, the genomic DNA was fragmented using Covaris followed by end repair and adapter ligation. Finally, the sequencing was performed on Illumina MiSeq (Illumina, USA) platform.

### Assembly and annotation

The raw paired-end fastq reads (2 × 301 bp) were quality checked using FastQC v.0.11.5^67^ followed by trimming of low-quality bases in a sliding window approach using Trimmomatic v.0.39^68^. The cleaned reads were assembled separately using the SPAdes v.3.13.0^69^, VelvetOptimiser v.2.2.4^70^, and ABySS v.1.9.0^71^. The comparative evaluation of the assemblies generated from the three assemblers was carried out using the Quality Assessment Tool (QUAST v.5.0.2)^72^. The best assembly (with the highest N50 length) found with the QUAST analysis proceeded further for genome assembly-assisted bacterial strain identification using the MiGA webserver^73^, StrainSeeker^74^, and TYGS server^75^. The contigs from the Velvet assembly were processed for scaffolding and gap filling using the programs SSPACE v3.0^76^ and GapFiller v.1.10^77^. Finally, the contigs were ordered using the Progressive Mauve^78^ and a similar reference genome of *Bordetella petrii* DSM 12804 (NC_010170.1) to obtain the draft assembly of BMCSI 3. The assembly quality and genomic contaminations, if any, were evaluated using the CheckM—v.1.0.18^79^ in KBase^80^. Further genomic analysis, annotation, and other comparative genomics studies were carried out using this BMCSI 3 draft assembly.

The contig assembly of BMCSI 3 was annotated using the software Prokka v.1.13.7^81^, Rapid Annotation System Technology (RAST) Pipeline^82^, PATRIC Pipeline^83^ and National Center for Biotechnology Information (NCBI) stand-alone Prokaryotic Genome Annotation Pipeline^84^. The visualization of the genome and its typical features was carried out using the CGviewer server beta^85^. Genomic Islands within the genome of BMCSI 3 were predicted using IslandViewer 4 server^86^.

### Comparative genomics

Genome sequences available in the public domain of eight representative strains of each *Bordetella* species i.e., *B*. *pertussis* Tohama I (NC_002929.2), *B. parapertussis* Bpp5 (NC_018828.1), *B. bronchiseptica* 253 (NC_019382.1), *B. trematum* H044680328 (NZ_LT546645.1), *B. holmesii* ATCC 51,541 (NZ_CP007494.1), *B. avium* 197 N (NC_010645.1), *B. hinzii* F582 (NZ_CP012076.1), *B. ansorpii* H050680373 (NZ_FKIF01000001.1), *B. flabilis* AU10664 (NZ_CP016172.1), *B. bronchialis* AU17976 (NZ_CP016171.1) and *B. pseudohinzii* HI4681 (NZ_CP016440.1) including all available strains of *B*. *petrii* were retrieved from the National Center for Biotechnology Information (NCBI) GenBank (https://www.ncbi.nlm.nih.gov/genome).

### Genome-informed differential characteristics

Selected representative species of the genus *Bordetella* including all available strains of *B. petrii* were compared to identify the potential differences, viz, the presence or absence of specific genes related to virulence factors, antibiotic resistance, and transporter using PATRIC server^87^. To study the relatedness of all the taxa, BLAST-based average nucleotide identity (ANIb) was investigated using JSpecies server^88^ and heatmap was produced in R version 4.1.1 using "pheatmap" package. For agglomeration of dendrogram, the default method "complete" was used^89^. Circular genome comparison was analyzed using CGView-Circular Genome Viewer^90^. Pangenome were analyzed using Spine and AGEnt tool^91^ considering all six strains of *B. petrii*. Core and accessory genomes were further annotated using RAST Pipeline^82^.

## Supplementary Information

Supplementary Information 1.

Supplementary Information 2.

Supplementary Information 3.

Supplementary Information 4.

## Data Availability

This whole-genome shotgun project was deposited at DDBJ/ENA/GenBank under the accession number JAFMZZ000000000, version JAFMZZ000000000.1 (BioProject PRJNA708297 and BioSample SAMN18228559).
