# Supplementary material for: Genomic, morphological, and biochemical analyses of a multi-metal resistant but multi-drug susceptible strain of Bordetella petrii from hospital soil
Source: Sci Rep. 2022 May 19;12:8439. doi: 10.1038/s41598-022-12435-7 (PMC9120033; doi:10.1038/s41598-022-12435-7)

**Supplementary File 1.**

**Assembly statistics of Strain BMCSI 3 in three genome assemblers**

The whole genome of BMCSI 3 was assembled using the three genome assemblers, SPAdes, Velvet, and ABySS; the latter two were based on the best suited K-mer statistics. For instance, the best assembly statistics were obtained with K-mer length of 99 in Velvet and k-mer length of 96 in ABySS. The contigs produced by the SPAdes, Velvet, and ABySS programs are 66, 14, and 114, respectively

| **Assembly** | **SPAdes** | **Velvet (kmer-99)** | **ABYSS (kmer-96)** |
| --- | --- | --- | --- |
| # contigs (>= 0 bp) | 66 | 14 | 114 |
| # contigs (>= 1000 bp) | 19 | 6 | 51 |
| # contigs (>= 5000 bp) | 16 | 3 | 40 |
| # contigs (>= 10000 bp) | 15 | 2 | 37 |
| # contigs (>= 25000 bp) | 13 | 2 | 33 |
| # contigs (>= 50000 bp) | 13 | 2 | 28 |
| Total length (>= 0 bp) | 4946935 | 4948225 | 4935740 |
| Total length (>= 1000 bp) | 4927595 | 4943940 | 4922127 |
| Total length (>= 5000 bp) | 4917532 | 4940362 | 4903855 |
| Total length (>= 10000 bp) | 4912064 | 4934471 | 4884187 |
| Total length (>= 25000 bp) | 4864999 | 4934471 | 4811229 |
| Total length (>= 50000 bp) | 4864999 | 4934471 | 4615658 |
| # contigs | 56 | 14 | 66 |
| Largest contig | 674329 | 3800126 | 586817 |
| Total length | 4944731 | 4948225 | 4928273 |
| GC (%) | 67.32 | 67.32 | 67.37 |
| N50 | 577700 | 3800126 | 215905 |
| N75 | 330381 | 3800126 | 103528 |
| L50 | 4 | 1 | 7 |
| L75 | 7 | 1 | 16 |
| # N's per 100 kbp | 0 | 6.67 | 0 |

**Completeness and quality of BMCSI 3 draft assemblies through CheckM tool:**

The marker lineage used was Burkholderiales with 193 genomes. 426 out of the 427 single-copy genes revealed 99.53% genome completeness with no heterogeneous sequence as contaminations.


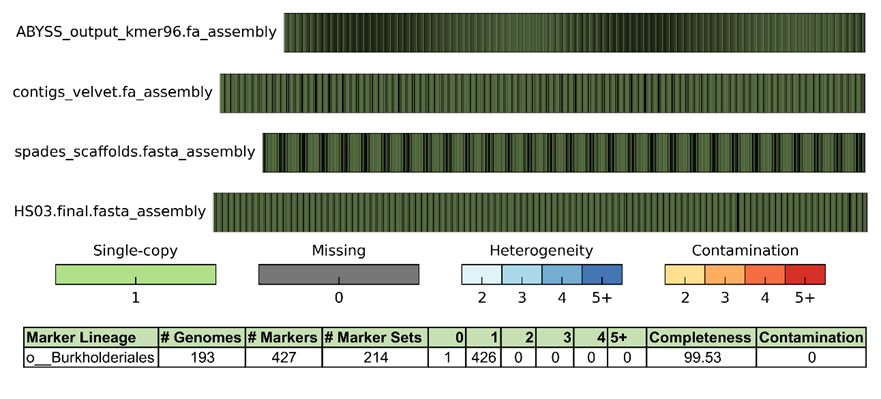

Supplement: Supplementary file 1 — Supplementary Information 1. [file 41598_2022_12435_MOESM1_ESM.docx]
