# Supplementary material for: Genomic, morphological, and biochemical analyses of a multi-metal resistant but multi-drug susceptible strain of Bordetella petrii from hospital soil
Source: Sci Rep. 2022 May 19;12:8439. doi: 10.1038/s41598-022-12435-7 (PMC9120033; doi:10.1038/s41598-022-12435-7)
Supplement: Supplementary file 2 — Supplementary Information 2. [file 41598_2022_12435_MOESM2_ESM.docx]

**Supplementary File 2.** Genomic Islands present in the genome of BMCSI 3

A total of 46 Genomic Islands (GIs) of BMCSI 3 were identified which covered about 20% of the total genome. Approximately, 10% (88,743 bp) of the genome is comprised of mobile genetic elements in the form of integrative and conjugative elements. Most of the putative laterally acquired GIs found in BMCSI 3 harbored heavy metal resistant genes, multiple response regulators and other transcription factors, transporters, and multi-drug efflux pumps

| **Island start** | **Island end** | **Length** | **Gene name** | **Gene ID** | **Gene start** | **Gene end** | **Product** |
| --- | --- | --- | --- | --- | --- | --- | --- |
| 217276 | 267455 | 50179 | WP_158310063.1 |  | 217276 | 218145 | winged helix-turn-helix domain-containing protein |
|  |  |  | WP_085970192.1 |  | 218226 | 219313 | IS3 family transposase |
|  |  |  | WP_158310064.1 |  | 219926 | 220318 | helix-turn-helix domain-containing protein |
|  |  |  | WP_085970193.1 |  | 220666 | 221753 | IS3 family transposase |
|  |  |  | WP_012247232.1 |  | 225573 | 227390 | AAA family ATPase |
|  |  |  | WP_085970194.1 |  | 228224 | 229344 | IS3 family transposase |
|  |  |  | WP_012247236.1 |  | 229877 | 230881 | TniQ family protein |
|  |  |  | WP_012247237.1 |  | 230905 | 232401 | helix-turn-helix domain-containing protein |
|  |  |  | WP_085970191.1 |  | 232551 | 233725 | IS3 family transposase |
|  |  |  |  |  | 234942 | 236494 | IS3 family transposase |
|  |  |  |  |  | 240858 | 240929 | integrase |
|  |  |  |  |  | 241034 | 241338 | transposase |
|  |  |  | WP_012247247.1 |  | 241480 | 245220 | restriction endonuclease |
|  |  |  | WP_012247249.1 |  | 246971 | 247684 | OmpA family protein |
|  |  |  | WP_151209006.1 | zorA | 247687 | 249651 | anti-phage defense ZorAB system ZorA |
|  |  |  |  |  | 250097 | 250833 | transposase |
|  |  |  | WP_081482953.1 |  | 251302 | 251616 | helix-turn-helix domain-containing protein |
|  |  |  | WP_012247255.1 |  | 253237 | 253503 | helix-turn-helix domain-containing protein |
|  |  |  | WP_012247256.1 |  | 253500 | 254810 | HipA domain-containing protein |
|  |  |  | WP_081482954.1 |  | 255305 | 256564 | nucleotide sugar dehydrogenase |
|  |  |  | WP_012247259.1 |  | 259080 | 261137 | glycosyltransferase |
|  |  |  | WP_012247260.1 |  | 261180 | 262337 | polysaccharide biosynthesis/export family protein |
|  |  |  | WP_012247261.1 |  | 262345 | 263685 | ABC transporter permease |
|  |  |  | WP_012247262.1 |  | 263695 | 264408 | ATPase |
|  |  |  | WP_012247263.1 |  | 264530 | 265558 | sugar ABC transporter |
|  |  |  | WP_012247264.1 | vexE | 265551 | 267455 | Vi polysaccharide transport protein VexE |
| 241480 | 254810 | 13330 | WP_012247247.1 |  | 241480 | 245220 | restriction endonuclease |
|  |  |  | WP_012247249.1 |  | 246971 | 247684 | OmpA family protein |
|  |  |  | WP_151209006.1 | zorA | 247687 | 249651 | anti-phage defense ZorAB system ZorA |
|  |  |  |  |  | 250097 | 250833 | transposase |
|  |  |  | WP_081482953.1 |  | 251302 | 251616 | helix-turn-helix domain-containing protein |
|  |  |  | WP_012247255.1 |  | 253237 | 253503 | helix-turn-helix domain-containing protein |
|  |  |  | WP_012247256.1 |  | 253500 | 254810 | HipA domain-containing protein |
| 1030589 | 1035202 | 4613 | WP_012247949.1 |  | 1029715 | 1030590 | GyrI-like domain-containing protein |
|  |  |  | WP_012247952.1 |  | 1032221 | 1032535 | DUF1153 domain-containing protein |
|  |  |  | WP_012247954.1 |  | 1033647 | 1034633 | site-specific integrase |
| 1048077 | 1053296 | 5219 | WP_012247973.1 |  | 1048456 | 1048776 | TM2 domain-containing protein |
|  |  |  | WP_173376860.1 |  | 1049332 | 1049931 | helix-turn-helix transcriptional regulator |
|  |  |  | WP_012247979.1 |  | 1052041 | 1052562 | DUF1367 family protein |
| 1057006 | 1062967 | 5961 | WP_012247987.1 |  | 1057383 | 1057961 | terminase small subunit |
|  |  |  | WP_012247988.1 | terL | 1057963 | 1059354 | phage terminase large subunit |
|  |  |  | WP_012247989.1 |  | 1059357 | 1060637 | DUF1073 domain-containing protein |
|  |  |  | WP_012247990.1 |  | 1060573 | 1061412 | minor capsid protein |
|  |  |  | WP_012247991.1 |  | 1061423 | 1062505 | DUF2213 domain-containing protein |
| 1070534 | 1154785 | 84251 | WP_012248002.1 |  | 1068675 | 1070537 | tape measure protein |
|  |  |  | WP_012248009.1 |  | 1074840 | 1075595 | DUF2612 domain-containing protein |
|  |  |  | WP_012248015.1 |  | 1079149 | 1079589 | glycoside hydrolase family protein |
|  |  |  | WP_012248017.1 |  | 1080336 | 1081418 | polysaccharide pyruvyl transferase family protein |
|  |  |  | WP_012248019.1 |  | 1082211 | 1082876 | SOS response-associated peptidase |
|  |  |  |  | arsH | 1084242 | 1084802 | arsenical resistance protein ArsH |
|  |  |  | WP_012248023.1 |  | 1085860 | 1086342 | helix-turn-helix domain-containing protein |
|  |  |  |  |  | 1086354 | 1087324 | arsenic resistance protein |
|  |  |  |  |  | 1087386 | 1088054 | chromosome partitioning protein ParB |
|  |  |  |  |  | 1088063 | 1088488 | transposase family protein |
|  |  |  |  |  | 1088536 | 1088760 | ATP-binding protein |
|  |  |  | WP_041863552.1 |  | 1089183 | 1090181 | cation transporter |
|  |  |  | WP_124084334.1 |  | 1090293 | 1091533 | IS3 family transposase |
|  |  |  | WP_012248032.1 | cadR | 1091695 | 1092108 | Cd(II)/Pb(II)-responsive transcriptional regulator |
|  |  |  |  |  | 1092235 | 1092756 | transposase |
|  |  |  | WP_041862728.1 | arsC | 1092800 | 1093222 | arsenate reductase (glutaredoxin) |
|  |  |  | WP_012248035.1 | arsB | 1093237 | 1094322 | ACR3 family arsenite efflux transporter |
|  |  |  | WP_012248036.1 |  | 1094333 | 1094830 | arsenate reductase ArsC |
|  |  |  | WP_012248037.1 |  | 1094843 | 1095313 | glyoxalase/bleomycin resistance/dioxygenase family protein |
|  |  |  | WP_041863555.1 |  | 1095326 | 1095655 | helix-turn-helix transcriptional regulator |
|  |  |  | WP_003090093.1 |  | 1097136 | 1097348 | AlpA family transcriptional regulator |
|  |  |  | WP_012248040.1 |  | 1097391 | 1098266 | ParA family protein |
|  |  |  | WP_012248042.1 |  | 1098512 | 1100191 | ParB family protein |
|  |  |  | WP_012248043.1 |  | 1100206 | 1100766 | DUF2857 domain-containing protein |
|  |  |  | WP_003090099.1 |  | 1102433 | 1103227 | TIGR03761 family integrating conjugative element protein |
|  |  |  | WP_012248045.1 |  | 1103224 | 1103751 | DUF3158 family protein |
|  |  |  | WP_003120036.1 |  | 1103826 | 1104278 | single-stranded DNA-binding protein |
|  |  |  | WP_012248046.1 |  | 1104554 | 1106575 | DNA topoisomerase III |
|  |  |  | WP_012248047.1 |  | 1107107 | 1107700 | DNA cytosine methyltransferase |
|  |  |  | WP_012248048.1 |  | 1107785 | 1108834 | tyrosine-type recombinase/integrase |
|  |  |  | WP_012248049.1 |  | 1108827 | 1109822 | tyrosine-type recombinase/integrase |
|  |  |  | WP_012248050.1 |  | 1109822 | 1111030 | tyrosine-type recombinase/integrase |
|  |  |  | WP_012248051.1 |  | 1111201 | 1112211 | DNA cytosine methyltransferase |
|  |  |  | WP_012248059.1 |  | 1117860 | 1118213 | DUF3085 domain-containing protein |
|  |  |  | WP_012248060.1 |  | 1118576 | 1119493 | DUF3577 domain-containing protein |
|  |  |  | WP_012248061.1 |  | 1119638 | 1120465 | DUF945 domain-containing protein |
|  |  |  | WP_003098913.1 |  | 1120560 | 1121249 | DUF3275 family protein |
|  |  |  | WP_012248063.1 | ltrA | 1122690 | 1124408 | group II intron reverse transcriptase/maturase |
|  |  |  | WP_012248065.1 | ltrA | 1126339 | 1128054 | group II intron reverse transcriptase/maturase |
|  |  |  | WP_041862730.1 |  | 1128122 | 1129132 | SAM-dependent methyltransferase |
|  |  |  | WP_041862731.1 |  | 1130035 | 1132320 | DEAD/DEAH box helicase |
|  |  |  | WP_012248071.1 |  | 1133464 | 1134063 | PilL N-terminal domain-containing protein |
|  |  |  | WP_012248073.1 |  | 1134719 | 1135444 | TIGR03759 family integrating conjugative element protein |
|  |  |  | WP_012248074.1 |  | 1135426 | 1136031 | transglycosylase SLT domain-containing protein |
|  |  |  | WP_012248075.1 |  | 1136028 | 1136576 | integrating conjugative element protein |
|  |  |  | WP_012248076.1 | traD | 1136581 | 1138770 | type IV conjugative transfer system coupling protein TraD |
|  |  |  | WP_012248077.1 |  | 1138767 | 1139516 | TIGR03747 family integrating conjugative element membrane protein |
|  |  |  | WP_012248078.1 |  | 1139615 | 1139998 | RAQPRD family integrative conjugative element protein |
|  |  |  | WP_012248079.1 |  | 1139995 | 1140228 | TIGR03758 family integrating conjugative element protein |
|  |  |  | WP_012248080.1 |  | 1140245 | 1140604 | TIGR03745 family integrating conjugative element membrane protein |
|  |  |  | WP_012248081.1 |  | 1140617 | 1141027 | TIGR03750 family conjugal transfer protein |
|  |  |  | WP_012248082.1 |  | 1141024 | 1141716 | TIGR03746 family integrating conjugative element protein |
|  |  |  | WP_012248083.1 |  | 1141713 | 1142630 | TIGR03749 family integrating conjugative element protein |
|  |  |  | WP_012248084.1 |  | 1142620 | 1144038 | TIGR03752 family integrating conjugative element protein |
|  |  |  | WP_012248085.1 |  | 1144019 | 1144468 | TIGR03751 family conjugal transfer lipoprotein |
|  |  |  | WP_012248086.1 |  | 1144468 | 1147356 | conjugative transfer ATPase |
|  |  |  | WP_012248087.1 |  | 1147370 | 1148134 | DsbA family protein |
|  |  |  | WP_012248088.1 | radC | 1148309 | 1148803 | DNA repair protein RadC |
|  |  |  | WP_012248089.1 |  | 1148967 | 1149413 | TIGR03757 family integrating conjugative element protein |
|  |  |  | WP_041862733.1 |  | 1149410 | 1150357 | TIGR03756 family integrating conjugative element protein |
|  |  |  | WP_012248091.1 |  | 1150367 | 1151761 | integrating conjugative element protein |
|  |  |  | WP_012248092.1 |  | 1151758 | 1152117 | hypothetical protein |
|  |  |  | WP_012248093.1 |  | 1152131 | 1153678 | conjugal transfer protein TraG |
|  |  |  | WP_012248094.1 |  | 1153706 | 1154071 | DUF3742 family protein |
|  |  |  | WP_012248095.1 |  | 1154153 | 1154785 | RES family NAD+ phosphorylase |
| 1122690 | 1128054 | 5364 | WP_012248063.1 | ltrA | 1122690 | 1124408 | group II intron reverse transcriptase/maturase |
|  |  |  | WP_012248065.1 | ltrA | 1126339 | 1128054 | group II intron reverse transcriptase/maturase |
| 1171311 | 1188022 | 16711 | WP_012248110.1 |  | 1171311 | 1172009 | FUSC family protein |
|  |  |  | WP_012248104.1 |  | 1172096 | 1173337 | ISL3 family transposase |
|  |  |  | WP_012248111.1 |  | 1173353 | 1174993 | hydantoinase/oxoprolinase family protein |
|  |  |  |  |  | 1175142 | 1176694 | IS3 family transposase |
|  |  |  | WP_012247241.1 |  | 1176920 | 1177411 | transposase |
|  |  |  | WP_041863569.1 |  | 1177661 | 1178806 | acyl-CoA dehydrogenase family protein |
|  |  |  | WP_012248113.1 |  | 1178858 | 1179925 | MBL fold metallo-hydrolase |
|  |  |  | WP_012248114.1 |  | 1179936 | 1181156 | phenylacetate--CoA ligase family protein |
|  |  |  | WP_012248115.1 |  | 1181184 | 1181972 | enoyl-CoA hydratase/isomerase family protein |
|  |  |  | WP_012248116.1 |  | 1182158 | 1183255 | CoA transferase |
|  |  |  | WP_012248117.1 |  | 1183379 | 1184617 | thiolase family protein |
|  |  |  | WP_012248118.1 |  | 1184682 | 1186235 | AMP-binding protein |
|  |  |  | WP_012248119.1 |  | 1186326 | 1187087 | SDR family NAD(P)-dependent oxidoreductase |
|  |  |  | WP_012248120.1 |  | 1187222 | 1188022 | ABC transporter ATP-binding protein |
| 1200644 | 1209438 | 8794 | WP_081482976.1 |  | 1201348 | 1202025 | peptidoglycan-binding protein |
|  |  |  |  |  | 1202027 | 1203579 | IS3 family transposase |
|  |  |  |  |  | 1203655 | 1203897 | peptidoglycan-binding protein |
|  |  |  | WP_012248136.1 |  | 1203985 | 1204383 | transposase |
|  |  |  | WP_012248137.1 | tnpB | 1204380 | 1204724 | IS66 family insertion sequence element accessory protein TnpB |
|  |  |  |  |  | 1205005 | 1205820 | IS66 family transposase |
|  |  |  | WP_158310073.1 |  | 1205850 | 1206005 | IS3 family transposase |
|  |  |  | WP_041863593.1 | istB | 1206043 | 1206840 | IS21-like element helper ATPase IstB |
|  |  |  | WP_012248141.1 | istA | 1206891 | 1208297 | IS21 family transposase |
|  |  |  | WP_012248143.1 | tnpB | 1208771 | 1209127 | IS66 family insertion sequence element accessory protein TnpB |
|  |  |  | WP_050978208.1 |  | 1209127 | 1209438 | transposase |
| 1215195 | 1219644 | 4449 | WP_041863596.1 |  | 1215195 | 1216418 | CoA transferase |
|  |  |  | WP_012248150.1 |  | 1216458 | 1216946 | MaoC family dehydratase N-terminal domain-containing protein |
|  |  |  | WP_012248151.1 |  | 1217014 | 1217979 | tripartite tricarboxylate transporter substrate binding protein |
|  |  |  | WP_012248152.1 |  | 1218053 | 1218841 | enoyl-CoA hydratase/isomerase family protein |
|  |  |  | WP_012248153.1 |  | 1218844 | 1219644 | enoyl-CoA hydratase/isomerase family protein |
|  |  |  | WP_012248154.1 |  | 1219641 | 1220426 | helix-turn-helix domain-containing protein |
| 1253396 | 1308633 | 55237 | WP_081483052.1 |  | 1253396 | 1254127 | LysR family transcriptional regulator |
|  |  |  | WP_012248191.1 |  | 1254072 | 1254761 | transposase |
|  |  |  |  |  | 1254794 | 1255303 | ISL3 family transposase |
|  |  |  |  |  | 1255334 | 1256886 | IS3 family transposase |
|  |  |  | WP_012248193.1 |  | 1257038 | 1257940 | LysR family transcriptional regulator |
|  |  |  | WP_158310075.1 |  | 1258034 | 1259491 | efflux transporter outer membrane subunit |
|  |  |  | WP_012248195.1 |  | 1259493 | 1260398 | HlyD family secretion protein |
|  |  |  | WP_151209021.1 |  | 1260400 | 1260558 | DUF1656 domain-containing protein |
|  |  |  | WP_012247241.1 |  | 1260635 | 1261126 | transposase |
|  |  |  | WP_012248196.1 |  | 1261123 | 1262052 | IS3 family transposase |
|  |  |  |  |  | 1262088 | 1263640 | IS3 family transposase |
|  |  |  | WP_012248198.1 |  | 1264178 | 1265152 | tripartite tricarboxylate transporter substrate binding protein |
|  |  |  | WP_012248199.1 |  | 1265291 | 1267405 | acetate--CoA ligase family protein |
|  |  |  | WP_012248200.1 |  | 1267550 | 1268704 | acyl-CoA/acyl-ACP dehydrogenase |
|  |  |  | WP_012248201.1 |  | 1269012 | 1269788 | enoyl-CoA hydratase/isomerase family protein |
|  |  |  | WP_012248202.1 |  | 1269788 | 1270567 | SDR family oxidoreductase |
|  |  |  | WP_012248203.1 |  | 1270606 | 1271748 | acyl-CoA dehydrogenase family protein |
|  |  |  | WP_012248204.1 |  | 1271745 | 1273913 | acetate--CoA ligase family protein |
|  |  |  | WP_012248205.1 |  | 1273910 | 1274902 | nitronate monooxygenase |
|  |  |  | WP_158310076.1 |  | 1275093 | 1275575 | TRAP transporter small permease subunit |
|  |  |  | WP_041862745.1 |  | 1275577 | 1276887 | TRAP transporter large permease |
|  |  |  | WP_012248208.1 |  | 1276937 | 1278043 | C4-dicarboxylate TRAP transporter substrate-binding protein |
|  |  |  | WP_012248209.1 |  | 1278140 | 1279336 | CoA transferase |
|  |  |  | WP_041862746.1 |  | 1279386 | 1280519 | CoA transferase |
|  |  |  | WP_012248211.1 |  | 1280560 | 1281315 | enoyl-CoA hydratase/isomerase family protein |
|  |  |  | WP_041862747.1 |  | 1281435 | 1282640 | CoA transferase |
|  |  |  | WP_012248213.1 |  | 1282677 | 1283537 | MaoC family dehydratase N-terminal domain-containing protein |
|  |  |  | WP_012248214.1 |  | 1283551 | 1284531 | tripartite tricarboxylate transporter substrate binding protein |
|  |  |  | WP_012248215.1 |  | 1284594 | 1285574 | tripartite tricarboxylate transporter substrate binding protein |
|  |  |  | WP_041863619.1 |  | 1285651 | 1286910 | CoA transferase |
|  |  |  | WP_012248217.1 |  | 1287007 | 1287477 | dehydratase |
|  |  |  | WP_151208941.1 |  | 1287543 | 1288721 | thiolase family protein |
|  |  |  | WP_012248219.1 |  | 1288730 | 1289143 | Zn-ribbon domain-containing OB-fold protein |
|  |  |  | WP_012248220.1 |  | 1289140 | 1289955 | enoyl-CoA hydratase/isomerase family protein |
|  |  |  | WP_012248222.1 |  | 1291257 | 1292246 | tripartite tricarboxylate transporter substrate binding protein |
|  |  |  | WP_041862750.1 |  | 1292634 | 1294274 | sigma 54-interacting transcriptional regulator |
|  |  |  | WP_012248224.1 |  | 1294509 | 1294973 | PaaI family thioesterase |
|  |  |  | WP_109433485.1 |  | 1294988 | 1296184 | ABC transporter substrate-binding protein |
|  |  |  | WP_109433486.1 |  | 1296532 | 1298235 | sigma 54-interacting transcriptional regulator |
|  |  |  | WP_012248227.1 |  | 1298385 | 1299632 | CoA transferase |
|  |  |  | WP_012248228.1 |  | 1299673 | 1301379 | acyl--CoA ligase |
|  |  |  | WP_012248229.1 |  | 1301376 | 1302146 | enoyl-CoA hydratase/isomerase family protein |
|  |  |  | WP_012248230.1 |  | 1302150 | 1302896 | SDR family oxidoreductase |
|  |  |  | WP_012248231.1 |  | 1302940 | 1304115 | acyl-CoA dehydrogenase family protein |
|  |  |  | WP_012248232.1 |  | 1304112 | 1304987 | 3-hydroxyacyl-CoA dehydrogenase family protein |
|  |  |  | WP_012248233.1 |  | 1304993 | 1305646 | 3-hydroxyacyl-CoA dehydrogenase |
|  |  |  | WP_012248234.1 |  | 1305643 | 1306776 | CoA transferase |
|  |  |  | WP_012248235.1 |  | 1306773 | 1307762 | NADPH:quinone oxidoreductase family protein |
|  |  |  | WP_012248236.1 |  | 1307824 | 1308633 | enoyl-CoA hydratase/isomerase family protein |
| 1255334 | 1269788 | 14454 |  |  | 1255334 | 1256886 | IS3 family transposase |
|  |  |  | WP_012248193.1 |  | 1257038 | 1257940 | LysR family transcriptional regulator |
|  |  |  | WP_158310075.1 |  | 1258034 | 1259491 | efflux transporter outer membrane subunit |
|  |  |  | WP_012248195.1 |  | 1259493 | 1260398 | HlyD family secretion protein |
|  |  |  | WP_151209021.1 |  | 1260400 | 1260558 | DUF1656 domain-containing protein |
|  |  |  | WP_012247241.1 |  | 1260635 | 1261126 | transposase |
|  |  |  | WP_012248196.1 |  | 1261123 | 1262052 | IS3 family transposase |
|  |  |  |  |  | 1262088 | 1263640 | IS3 family transposase |
|  |  |  | WP_012248198.1 |  | 1264178 | 1265152 | tripartite tricarboxylate transporter substrate binding protein |
|  |  |  | WP_012248199.1 |  | 1265291 | 1267405 | acetate--CoA ligase family protein |
|  |  |  | WP_012248200.1 |  | 1267550 | 1268704 | acyl-CoA/acyl-ACP dehydrogenase |
|  |  |  | WP_012248201.1 |  | 1269012 | 1269788 | enoyl-CoA hydratase/isomerase family protein |
| 1276937 | 1281315 | 4378 | WP_012248208.1 |  | 1276937 | 1278043 | C4-dicarboxylate TRAP transporter substrate-binding protein |
|  |  |  | WP_012248209.1 |  | 1278140 | 1279336 | CoA transferase |
|  |  |  | WP_041862746.1 |  | 1279386 | 1280519 | CoA transferase |
|  |  |  | WP_012248211.1 |  | 1280560 | 1281315 | enoyl-CoA hydratase/isomerase family protein |
| 1330119 | 1341819 | 11700 | WP_012248255.1 |  | 1330119 | 1330844 | amino acid ABC transporter ATP-binding protein |
|  |  |  | WP_012248256.1 |  | 1330837 | 1331499 | amino acid ABC transporter permease |
|  |  |  | WP_012248257.1 |  | 1331503 | 1332168 | amino acid ABC transporter permease |
|  |  |  | WP_012248258.1 |  | 1332232 | 1333071 | transporter substrate-binding domain-containing protein |
|  |  |  | WP_012248259.1 |  | 1333155 | 1334057 | dihydrodipicolinate synthase family protein |
|  |  |  | WP_041863633.1 |  | 1334386 | 1335066 | GntR family transcriptional regulator |
|  |  |  | WP_012248261.1 |  | 1335320 | 1336210 | LysR family transcriptional regulator |
|  |  |  | WP_012248262.1 |  | 1336235 | 1336528 | helix-turn-helix transcriptional regulator |
|  |  |  | WP_012248263.1 |  | 1336636 | 1337280 | LysR family transcriptional regulator |
|  |  |  | WP_012248264.1 |  | 1337333 | 1339258 | tyrosine-type recombinase/integrase |
|  |  |  | WP_012248265.1 |  | 1339793 | 1340359 | DUF2478 domain-containing protein |
|  |  |  | WP_012248266.1 |  | 1340356 | 1341150 | class I SAM-dependent methyltransferase |
|  |  |  | WP_012248267.1 |  | 1341169 | 1341819 | glutathione S-transferase |
| 1349423 | 1356971 | 7548 | WP_012248277.1 |  | 1350756 | 1351655 | LysR family transcriptional regulator |
|  |  |  | WP_012248278.1 |  | 1352135 | 1352347 | AlpA family phage regulatory protein |
|  |  |  | WP_012248279.1 |  | 1352385 | 1353248 | ParA family protein |
|  |  |  | WP_012248281.1 |  | 1353482 | 1355206 | ParB family protein |
|  |  |  | WP_012248282.1 |  | 1355218 | 1355778 | DUF2857 domain-containing protein |
| 1362023 | 1403058 | 41035 | WP_085970191.1 |  | 1362023 | 1363197 | IS3 family transposase |
|  |  |  | WP_012248289.1 |  | 1363278 | 1364081 | topoisomerase DNA-binding C4 zinc finger domain-containing protein |
|  |  |  | WP_012248290.1 |  | 1364635 | 1365204 | DNA cytosine methyltransferase |
|  |  |  | WP_012248050.1 |  | 1365341 | 1366549 | tyrosine-type recombinase/integrase |
|  |  |  | WP_012248049.1 |  | 1366549 | 1367544 | tyrosine-type recombinase/integrase |
|  |  |  | WP_012248048.1 |  | 1367537 | 1368586 | tyrosine-type recombinase/integrase |
|  |  |  | WP_151209025.1 |  | 1369122 | 1369904 | DNA cytosine methyltransferase |
|  |  |  | WP_012248293.1 |  | 1370007 | 1370522 | GNAT family N-acetyltransferase |
|  |  |  | WP_012248294.1 |  | 1370522 | 1370800 | DUF1778 domain-containing protein |
|  |  |  | WP_012248298.1 |  | 1374834 | 1375199 | DUF3085 domain-containing protein |
|  |  |  | WP_012248300.1 |  | 1376054 | 1376602 | DUF3577 domain-containing protein |
|  |  |  | WP_012248301.1 |  | 1376709 | 1377317 | DUF3275 family protein |
|  |  |  | WP_012248303.1 |  | 1377971 | 1378798 | DUF945 domain-containing protein |
|  |  |  | WP_012248305.1 |  | 1379652 | 1380758 | class I SAM-dependent methyltransferase |
|  |  |  | WP_012248309.1 |  | 1382099 | 1384372 | DEAD/DEAH box helicase |
|  |  |  | WP_012248310.1 |  | 1384388 | 1385263 | helix-turn-helix domain-containing protein |
|  |  |  | WP_012248311.1 | hemA | 1385419 | 1386660 | 5-aminolevulinate synthase |
|  |  |  | WP_012248312.1 |  | 1386661 | 1387161 | GNAT family N-acetyltransferase |
|  |  |  | WP_012248313.1 |  | 1387158 | 1388225 | Gfo/Idh/MocA family oxidoreductase |
|  |  |  | WP_012248314.1 | asnB | 1388255 | 1390108 | asparagine synthase (glutamine-hydrolyzing) |
|  |  |  | WP_197535842.1 |  | 1390166 | 1391332 | MFS transporter |
|  |  |  | WP_012248316.1 |  | 1392232 | 1393371 | FAD-dependent monooxygenase |
|  |  |  | WP_012248317.1 |  | 1393394 | 1395133 | FAD-dependent monooxygenase |
|  |  |  | WP_012248318.1 |  | 1395199 | 1395825 | TetR/AcrR family transcriptional regulator |
|  |  |  | WP_012248319.1 |  | 1395907 | 1397118 | MFS transporter |
|  |  |  | WP_012248320.1 |  | 1397137 | 1397862 | DMT family transporter |
|  |  |  | WP_041862764.1 |  | 1397873 | 1398787 | lysine exporter LysO family protein |
|  |  |  | WP_012248322.1 |  | 1398784 | 1399206 | 2OG-Fe dioxygenase family protein |
|  |  |  | WP_085970191.1 |  | 1399285 | 1400459 | IS3 family transposase |
|  |  |  | WP_012248324.1 |  | 1400898 | 1401554 | LysE family translocator |
|  |  |  | WP_197535843.1 |  | 1401556 | 1402290 | 2OG-Fe(II) oxygenase |
|  |  |  | WP_012248326.1 |  | 1402426 | 1403058 | alanyl-tRNA synthetase |
| 1364635 | 1375646 | 11011 | WP_012248290.1 |  | 1364635 | 1365204 | DNA cytosine methyltransferase |
|  |  |  | WP_012248050.1 |  | 1365341 | 1366549 | tyrosine-type recombinase/integrase |
|  |  |  | WP_012248049.1 |  | 1366549 | 1367544 | tyrosine-type recombinase/integrase |
|  |  |  | WP_012248048.1 |  | 1367537 | 1368586 | tyrosine-type recombinase/integrase |
|  |  |  | WP_151209025.1 |  | 1369122 | 1369904 | DNA cytosine methyltransferase |
|  |  |  | WP_012248293.1 |  | 1370007 | 1370522 | GNAT family N-acetyltransferase |
|  |  |  | WP_012248294.1 |  | 1370522 | 1370800 | DUF1778 domain-containing protein |
|  |  |  | WP_012248298.1 |  | 1374834 | 1375199 | DUF3085 domain-containing protein |
| 1376709 | 1407326 | 30617 | WP_012248301.1 |  | 1376709 | 1377317 | DUF3275 family protein |
|  |  |  | WP_012248303.1 |  | 1377971 | 1378798 | DUF945 domain-containing protein |
|  |  |  | WP_012248305.1 |  | 1379652 | 1380758 | class I SAM-dependent methyltransferase |
|  |  |  | WP_012248309.1 |  | 1382099 | 1384372 | DEAD/DEAH box helicase |
|  |  |  | WP_012248310.1 |  | 1384388 | 1385263 | helix-turn-helix domain-containing protein |
|  |  |  | WP_012248311.1 | hemA | 1385419 | 1386660 | 5-aminolevulinate synthase |
|  |  |  | WP_012248312.1 |  | 1386661 | 1387161 | GNAT family N-acetyltransferase |
|  |  |  | WP_012248313.1 |  | 1387158 | 1388225 | Gfo/Idh/MocA family oxidoreductase |
|  |  |  | WP_012248314.1 | asnB | 1388255 | 1390108 | asparagine synthase (glutamine-hydrolyzing) |
|  |  |  | WP_197535842.1 |  | 1390166 | 1391332 | MFS transporter |
|  |  |  | WP_012248316.1 |  | 1392232 | 1393371 | FAD-dependent monooxygenase |
|  |  |  | WP_012248317.1 |  | 1393394 | 1395133 | FAD-dependent monooxygenase |
|  |  |  | WP_012248318.1 |  | 1395199 | 1395825 | TetR/AcrR family transcriptional regulator |
|  |  |  | WP_012248319.1 |  | 1395907 | 1397118 | MFS transporter |
|  |  |  | WP_012248320.1 |  | 1397137 | 1397862 | DMT family transporter |
|  |  |  | WP_041862764.1 |  | 1397873 | 1398787 | lysine exporter LysO family protein |
|  |  |  | WP_012248322.1 |  | 1398784 | 1399206 | 2OG-Fe dioxygenase family protein |
|  |  |  | WP_085970191.1 |  | 1399285 | 1400459 | IS3 family transposase |
|  |  |  | WP_012248324.1 |  | 1400898 | 1401554 | LysE family translocator |
|  |  |  | WP_197535843.1 |  | 1401556 | 1402290 | 2OG-Fe(II) oxygenase |
|  |  |  | WP_012248326.1 |  | 1402426 | 1403058 | alanyl-tRNA synthetase |
|  |  |  | WP_012248327.1 |  | 1403154 | 1404140 | LysR family transcriptional regulator |
|  |  |  | WP_012248328.1 |  | 1404231 | 1404815 | PilL N-terminal domain-containing protein |
|  |  |  | WP_012248330.1 |  | 1405469 | 1406209 | TIGR03759 family integrating conjugative element protein |
|  |  |  | WP_012248331.1 |  | 1406194 | 1406778 | transglycosylase SLT domain-containing protein |
|  |  |  | WP_012248332.1 |  | 1406775 | 1407326 | integrating conjugative element protein |
| 1423236 | 1440610 | 17374 | WP_151209027.1 |  | 1423236 | 1423943 | thioredoxin domain-containing protein |
|  |  |  | WP_012248349.1 |  | 1423971 | 1424603 | ParB-like nuclease domain-containing protein |
|  |  |  | WP_041862769.1 |  | 1425030 | 1426256 | DUF3440 domain-containing protein |
|  |  |  | WP_050978219.1 |  | 1426282 | 1427016 | helix-turn-helix transcriptional regulator |
|  |  |  | WP_197535837.1 | dosP | 1427315 | 1429894 | oxygen-sensing cyclic-di-GMP phosphodiesterase |
|  |  |  | WP_012248353.1 |  | 1430684 | 1432189 | PAS domain-containing methyl-accepting chemotaxis protein |
|  |  |  | WP_012248354.1 |  | 1432239 | 1433117 | LysR family transcriptional regulator |
|  |  |  | WP_012248355.1 | tdo | 1433241 | 1434017 | L-threonine 4-hydroxylase Tdo |
|  |  |  | WP_012248356.1 |  | 1434074 | 1434691 | LysE family translocator |
|  |  |  | WP_012248357.1 | thrH | 1434704 | 1435333 | bifunctional phosphoserine phosphatase/homoserine phosphotransferase ThrH |
|  |  |  | WP_012248358.1 |  | 1435548 | 1435997 | TIGR03757 family integrating conjugative element protein |
|  |  |  | WP_012248359.1 |  | 1435994 | 1436938 | TIGR03756 family integrating conjugative element protein |
|  |  |  | WP_012248360.1 |  | 1436940 | 1438346 | integrating conjugative element protein |
|  |  |  | WP_012248362.1 |  | 1438707 | 1440224 | conjugal transfer protein TraG |
|  |  |  | WP_012248363.1 |  | 1440233 | 1440610 | DUF3742 family protein |
| 1456182 | 1556286 | 100104 | WP_012248380.1 |  | 1456182 | 1456955 | 1,6-dihydroxycyclohexa-2,4-diene-1-carboxylate dehydrogenase |
|  |  |  | WP_012248381.1 |  | 1456968 | 1457993 | ring-hydroxylating dioxygenase ferredoxin reductase family protein |
|  |  |  | WP_012248382.1 | benB | 1458011 | 1458511 | benzoate 1,2-dioxygenase small subunit |
|  |  |  | WP_012248383.1 |  | 1458508 | 1459872 | Rieske 2Fe-2S domain-containing protein |
|  |  |  | WP_012248384.1 | catA | 1459999 | 1460934 | catechol 1,2-dioxygenase |
|  |  |  | WP_041862778.1 |  | 1460977 | 1462173 | muconate cycloisomerase |
|  |  |  | WP_041863662.1 |  | 1462315 | 1463271 | LysR family transcriptional regulator |
|  |  |  |  |  | 1463695 | 1464458 | IS5 family transposase |
|  |  |  |  |  | 1464607 | 1464735 | transposase |
|  |  |  |  |  | 1464809 | 1465030 | IS3 family transposase |
|  |  |  | WP_012248392.1 |  | 1465555 | 1466010 | helix-turn-helix domain containing protein |
|  |  |  | WP_012248393.1 |  | 1466321 | 1468411 | recombinase family protein |
|  |  |  |  |  | 1468417 | 1469429 | IS3 family transposase |
|  |  |  | WP_012248396.1 |  | 1469516 | 1470343 | CbtA family protein |
|  |  |  | WP_012248397.1 |  | 1470523 | 1470714 | CbtB-domain containing protein |
|  |  |  | WP_197535838.1 |  | 1470755 | 1471195 | nitrile hydratase accessory protein |
|  |  |  | WP_012248399.1 | nthB | 1471192 | 1471848 | nitrile hydratase subunit beta |
|  |  |  | WP_034112801.1 | nthA | 1471859 | 1472494 | nitrile hydratase subunit alpha |
|  |  |  | WP_012248401.1 |  | 1472754 | 1474160 | Asp-tRNA(Asn)/Glu-tRNA(Gln) amidotransferase GatCAB subunit A |
|  |  |  |  |  | 1474518 | 1475280 | IS5 family transposase |
|  |  |  | WP_012248404.1 |  | 1475915 | 1477492 | methyl-accepting chemotaxis protein |
|  |  |  | WP_081482989.1 |  | 1480499 | 1480792 | transposase |
|  |  |  | WP_012248409.1 |  | 1480737 | 1481072 | 3-oxoacid CoA-transferase subunit A |
|  |  |  | WP_012248411.1 |  | 1481766 | 1482545 | IclR family transcriptional regulator |
|  |  |  | WP_012248412.1 |  | 1482530 | 1483702 | 3-hydroxybenzoate 6-monooxygenase |
|  |  |  | WP_012248413.1 |  | 1483719 | 1484573 | fumarylacetoacetate hydrolase family protein |
|  |  |  | WP_012248414.1 | gtdA | 1484603 | 1485646 | gentisate 1,2-dioxygenase |
|  |  |  | WP_012248415.1 |  | 1485814 | 1487190 | MFS transporter |
|  |  |  | WP_012248416.1 |  | 1487225 | 1487650 | recombinase family protein |
|  |  |  |  |  | 1487803 | 1488005 | transcriptional regulator |
|  |  |  | WP_012248417.1 |  | 1488052 | 1488378 | helix-turn-helix transcriptional regulator |
|  |  |  | WP_012248418.1 |  | 1488485 | 1489135 | LysR family substrate-binding domain-containing protein |
|  |  |  | WP_012248419.1 |  | 1489427 | 1490008 | TetR/AcrR family transcriptional regulator |
|  |  |  | WP_012248420.1 |  | 1490093 | 1491262 | MFS transporter |
|  |  |  | WP_012248421.1 |  | 1491479 | 1493314 | tyrosine-type recombinase/integrase |
|  |  |  | WP_003460278.1 |  | 1495220 | 1495432 | AlpA family phage regulatory protein |
|  |  |  | WP_011489277.1 |  | 1495476 | 1496351 | ParA family protein |
|  |  |  | WP_011489278.1 |  | 1496585 | 1498237 | ParB N-terminal domain-containing protein |
|  |  |  | WP_011489279.1 |  | 1498253 | 1498813 | DUF2857 domain-containing protein |
|  |  |  | WP_011489281.1 |  | 1500392 | 1501171 | TIGR03761 family integrating conjugative element protein |
|  |  |  | WP_003460292.1 |  | 1501168 | 1501695 | DUF3158 family protein |
|  |  |  | WP_011489282.1 |  | 1501769 | 1502209 | single-stranded DNA-binding protein |
|  |  |  | WP_011489283.1 |  | 1502488 | 1504500 | DNA topoisomerase III |
|  |  |  | WP_012248422.1 |  | 1505037 | 1505642 | DNA cytosine methyltransferase |
|  |  |  | WP_012248050.1 |  | 1505779 | 1506987 | tyrosine-type recombinase/integrase |
|  |  |  | WP_012248049.1 |  | 1506987 | 1507982 | tyrosine-type recombinase/integrase |
|  |  |  | WP_085970191.1 |  | 1508109 | 1509283 | IS3 family transposase |
|  |  |  |  |  | 1509344 | 1510309 | tyrosine-type recombinase/integrase |
|  |  |  |  |  | 1510392 | 1511417 | DNA cytosine methyltransferase |
|  |  |  |  |  | 1515515 | 1516219 | GTPase |
|  |  |  | WP_085970191.1 |  | 1516254 | 1517428 | IS3 family transposase |
|  |  |  | WP_003290193.1 |  | 1517756 | 1518109 | DUF3085 domain-containing protein |
|  |  |  | WP_011489292.1 |  | 1518255 | 1519097 | phosphoadenosine phosphosulfate reductase family protein |
|  |  |  | WP_003290197.1 |  | 1519875 | 1520792 | DUF3577 domain-containing protein |
|  |  |  | WP_012248427.1 |  | 1520850 | 1521539 | DUF3275 family protein |
|  |  |  | WP_011489296.1 |  | 1523540 | 1524649 | O-methyltransferase |
|  |  |  |  |  | 1524749 | 1525066 | DEAD/DEAH box helicase |
|  |  |  |  |  | 1527604 | 1529565 | DEAD/DEAH box helicase |
|  |  |  | WP_011489299.1 |  | 1530709 | 1531308 | PilL N-terminal domain-containing protein |
|  |  |  | WP_003290214.1 |  | 1531970 | 1532689 | TIGR03759 family integrating conjugative element protein |
|  |  |  | WP_011489301.1 |  | 1532671 | 1533261 | transglycosylase SLT domain-containing protein |
|  |  |  | WP_003460312.1 |  | 1533258 | 1533806 | integrating conjugative element protein |
|  |  |  | WP_003290220.1 | traD | 1533811 | 1535997 | type IV conjugative transfer system coupling protein TraD |
|  |  |  | WP_003290222.1 |  | 1535994 | 1536743 | TIGR03747 family integrating conjugative element membrane protein |
|  |  |  | WP_011489303.1 |  | 1537370 | 1537753 | RAQPRD family integrative conjugative element protein |
|  |  |  | WP_003050225.1 |  | 1537750 | 1537983 | TIGR03758 family integrating conjugative element protein |
|  |  |  | WP_003290228.1 |  | 1538000 | 1538359 | TIGR03745 family integrating conjugative element membrane protein |
|  |  |  | WP_003290229.1 |  | 1538372 | 1538782 | TIGR03750 family conjugal transfer protein |
|  |  |  | WP_003460319.1 |  | 1538779 | 1539471 | TIGR03746 family integrating conjugative element protein |
|  |  |  | WP_003290231.1 |  | 1539468 | 1540400 | TIGR03749 family integrating conjugative element protein |
|  |  |  | WP_011489305.1 |  | 1540390 | 1541808 | TIGR03752 family integrating conjugative element protein |
|  |  |  | WP_003290235.1 |  | 1541789 | 1542229 | TIGR03751 family conjugal transfer lipoprotein |
|  |  |  |  |  | 1542229 | 1543284 | TraC family protein |
|  |  |  | WP_085970191.1 |  | 1543350 | 1544524 | IS3 family transposase |
|  |  |  |  |  | 1544564 | 1546381 | conjugative transfer ATPase |
|  |  |  | WP_011489307.1 |  | 1546395 | 1547159 | thioredoxin domain-containing protein |
|  |  |  |  |  | 1547343 | 1547681 | DNA repair protein RadC |
|  |  |  | WP_012248433.1 |  | 1547845 | 1549104 | tyrosine-type recombinase/integrase |
|  |  |  | WP_012248434.1 |  | 1549101 | 1550093 | tyrosine-type recombinase/integrase |
|  |  |  | WP_012248435.1 |  | 1550090 | 1551100 | tyrosine-type recombinase/integrase |
|  |  |  |  |  | 1551280 | 1551441 | DNA repair protein RadC |
|  |  |  | WP_011489309.1 |  | 1551600 | 1552046 | TIGR03757 family integrating conjugative element protein |
|  |  |  | WP_011489310.1 |  | 1552043 | 1552990 | TIGR03756 family integrating conjugative element protein |
|  |  |  | WP_085953625.1 |  | 1553060 | 1554397 | integrating conjugative element protein |
|  |  |  | WP_011489313.1 |  | 1554769 | 1556286 | conjugal transfer protein TraG |
| 1474157 | 1493314 | 19157 | WP_012248401.1 |  | 1472754 | 1474160 | Asp-tRNA(Asn)/Glu-tRNA(Gln) amidotransferase GatCAB subunit A |
|  |  |  |  |  | 1474518 | 1475280 | IS5 family transposase |
|  |  |  | WP_012248404.1 |  | 1475915 | 1477492 | methyl-accepting chemotaxis protein |
|  |  |  | WP_081482989.1 |  | 1480499 | 1480792 | transposase |
|  |  |  | WP_012248409.1 |  | 1480737 | 1481072 | 3-oxoacid CoA-transferase subunit A |
|  |  |  | WP_012248411.1 |  | 1481766 | 1482545 | IclR family transcriptional regulator |
|  |  |  | WP_012248412.1 |  | 1482530 | 1483702 | 3-hydroxybenzoate 6-monooxygenase |
|  |  |  | WP_012248413.1 |  | 1483719 | 1484573 | fumarylacetoacetate hydrolase family protein |
|  |  |  | WP_012248414.1 | gtdA | 1484603 | 1485646 | gentisate 1,2-dioxygenase |
|  |  |  | WP_012248415.1 |  | 1485814 | 1487190 | MFS transporter |
|  |  |  | WP_012248416.1 |  | 1487225 | 1487650 | recombinase family protein |
|  |  |  |  |  | 1487803 | 1488005 | transcriptional regulator |
|  |  |  | WP_012248417.1 |  | 1488052 | 1488378 | helix-turn-helix transcriptional regulator |
|  |  |  | WP_012248418.1 |  | 1488485 | 1489135 | LysR family substrate-binding domain-containing protein |
|  |  |  | WP_012248419.1 |  | 1489427 | 1490008 | TetR/AcrR family transcriptional regulator |
|  |  |  | WP_012248420.1 |  | 1490093 | 1491262 | MFS transporter |
|  |  |  | WP_012248421.1 |  | 1491479 | 1493314 | tyrosine-type recombinase/integrase |
| 1567491 | 1579115 | 11624 | WP_041862790.1 | gorA | 1566127 | 1567494 | glutathione-disulfide reductase |
|  |  |  | WP_151209032.1 |  | 1567491 | 1568105 | glutathione S-transferase |
|  |  |  | WP_012248448.1 |  | 1568129 | 1568749 | glutathione S-transferase N-terminal domain-containing protein |
|  |  |  | WP_012248449.1 |  | 1568763 | 1569353 | malonic semialdehyde reductase |
|  |  |  | WP_012248450.1 |  | 1569368 | 1569670 | Dabb family protein |
|  |  |  | WP_012248451.1 |  | 1569683 | 1569988 | YciI family protein |
|  |  |  |  |  | 1570186 | 1571139 | IS91-like element ISPps1 family transposase |
|  |  |  |  |  | 1571134 | 1571988 | DegT/DnrJ/EryC1/StrS aminotransferase family protein |
|  |  |  | WP_041862792.1 |  | 1571985 | 1572680 | class I SAM-dependent methyltransferase |
|  |  |  | WP_012248453.1 |  | 1572677 | 1573705 | glycosyltransferase family 2 protein |
|  |  |  | WP_012248454.1 |  | 1573702 | 1574760 | glycosyltransferase |
|  |  |  | WP_012248455.1 |  | 1574753 | 1575355 | acyltransferase |
|  |  |  | WP_041862793.1 |  | 1575339 | 1576571 | oligosaccharide flippase family protein |
|  |  |  | WP_012248457.1 |  | 1576782 | 1577165 | IS91 family transposase |
|  |  |  | WP_012248458.1 |  | 1577568 | 1579115 | IS91-like element ISPps1 family transposase |
| 1580078 | 1587673 | 7595 |  |  | 1580078 | 1580688 | LysE family transporter |
|  |  |  | WP_012248462.1 |  | 1580832 | 1581716 | LysR family transcriptional regulator |
|  |  |  | WP_012248463.1 |  | 1581886 | 1582668 | chlorocatechol 1,2-dioxygenase |
|  |  |  | WP_011489354.1 |  | 1582665 | 1583777 | muconate cycloisomerase family protein |
|  |  |  | WP_012248464.1 |  | 1583804 | 1584787 | tripartite tricarboxylate transporter substrate binding protein |
|  |  |  | WP_011489356.1 |  | 1584809 | 1585519 | dienelactone hydrolase family protein |
|  |  |  | WP_011489357.1 |  | 1585516 | 1586574 | maleylacetate reductase |
|  |  |  | WP_011489358.1 |  | 1586690 | 1587673 | AraC family transcriptional regulator |
| 2176477 | 2180522 | 4045 | WP_197535839.1 |  | 2176477 | 2176902 | CopD family protein |
|  |  |  | WP_012249003.1 |  | 2176902 | 2177279 | DUF3817 domain-containing protein |
|  |  |  | WP_041862854.1 |  | 2177434 | 2178390 | helix-turn-helix domain-containing protein |
|  |  |  | WP_012249005.1 |  | 2178529 | 2179428 | LysR family transcriptional regulator |
|  |  |  | WP_012249006.1 |  | 2179533 | 2180522 | zinc-dependent alcohol dehydrogenase family protein |
| 2251969 | 2258130 | 6161 | WP_012249077.1 |  | 2251969 | 2252328 | type II toxin-antitoxin system RelE/ParE family toxin |
|  |  |  | WP_012249078.1 |  | 2252332 | 2252652 | XRE family transcriptional regulator |
|  |  |  | WP_012249081.1 |  | 2253871 | 2255667 | DUF2326 domain-containing protein |
|  |  |  |  | radC | 2256002 | 2256507 | DNA repair protein RadC |
|  |  |  | WP_012249083.1 |  | 2256792 | 2257142 | DUF2958 domain-containing protein |
| 2279687 | 2284901 | 5214 | WP_012249109.1 |  | 2279687 | 2280583 | LysR family transcriptional regulator |
|  |  |  | WP_197535844.1 |  | 2280775 | 2281881 | threonine aldolase family protein |
|  |  |  | WP_012249111.1 |  | 2282008 | 2282391 | tautomerase family protein |
|  |  |  | WP_041863814.1 |  | 2282396 | 2283625 | MFS transporter |
|  |  |  | WP_012249113.1 |  | 2283682 | 2284626 | LysR family transcriptional regulator |
|  |  |  | WP_012249114.1 |  | 2284623 | 2284901 | EexN family lipoprotein |
| 2380364 | 2401229 | 20865 | WP_151209041.1 |  | 2380364 | 2381371 | phytanoyl-CoA dioxygenase family protein |
|  |  |  | WP_041862866.1 |  | 2381349 | 2382140 | SDR family NAD(P)-dependent oxidoreductase |
|  |  |  | WP_012249206.1 |  | 2382133 | 2383683 | LTA synthase family protein |
|  |  |  | WP_012249207.1 |  | 2383914 | 2385020 | ABC transporter permease |
|  |  |  | WP_197535845.1 |  | 2385039 | 2386136 | polysaccharide biosynthesis/export family protein |
|  |  |  | WP_012249209.1 |  | 2386145 | 2386930 | ABC transporter permease |
|  |  |  | WP_012249210.1 |  | 2386927 | 2387586 | ABC transporter ATP-binding protein |
|  |  |  | WP_012249211.1 |  | 2387673 | 2391293 | beta-3-deoxy-D-manno-oct-2-ulosonic acid transferase |
|  |  |  | WP_012249213.1 | pseB | 2392373 | 2393371 | UDP-N-acetylglucosamine 4,6-dehydratase (inverting) |
|  |  |  | WP_012249214.1 | pseC | 2393374 | 2394534 | UDP-4-amino-4, 6-dideoxy-N-acetyl-beta-L-altrosamine transaminase |
|  |  |  | WP_197535846.1 | pseF | 2394528 | 2395232 | pseudaminic acid cytidylyltransferase |
|  |  |  | WP_151208958.1 | pseG | 2395229 | 2396323 | UDP-2,4-diacetamido-2,4, 6-trideoxy-beta-L-altropyranose hydrolase |
|  |  |  | WP_197535841.1 | pseH | 2396320 | 2396838 | UDP-4-amino-4, 6-dideoxy-N-acetyl-beta-L-altrosamine N-acetyltransferase |
|  |  |  | WP_012249218.1 | pseI | 2396847 | 2397902 | pseudaminic acid synthase |
|  |  |  | WP_151209042.1 |  | 2397971 | 2400016 | capsular polysaccharide biosynthesis protein |
|  |  |  | WP_012249220.1 |  | 2400018 | 2401229 | capsular biosynthesis protein |
| 3026294 | 3035297 | 9003 | WP_012249787.1 |  | 3026294 | 3027436 | CoA transferase |
|  |  |  | WP_012249788.1 |  | 3027554 | 3028456 | LysR family transcriptional regulator |
|  |  |  | WP_012249789.1 |  | 3028779 | 3029987 | tyrosine-type recombinase/integrase |
|  |  |  | WP_012249790.1 |  | 3030078 | 3030281 | AlpA family transcriptional regulator |
|  |  |  | WP_012249794.1 |  | 3034854 | 3035297 | terminase small subunit |
| 3028779 | 3037468 | 8689 | WP_012249789.1 |  | 3028779 | 3029987 | tyrosine-type recombinase/integrase |
|  |  |  | WP_012249790.1 |  | 3030078 | 3030281 | AlpA family transcriptional regulator |
|  |  |  | WP_012249794.1 |  | 3034854 | 3035297 | terminase small subunit |
|  |  |  | WP_012249796.1 |  | 3035531 | 3037468 | phage tail tape measure protein |
| 3153538 | 3158192 | 4654 | WP_085970191.1 |  | 3153538 | 3154712 | IS3 family transposase |
|  |  |  | WP_012249921.1 |  | 3154791 | 3158192 | tetratricopeptide repeat protein |
| 3912548 | 3930880 | 18332 | WP_151209067.1 |  | 3912548 | 3913861 | integrase arm-type DNA-binding domain-containing protein |
|  |  |  | WP_012250600.1 |  | 3914296 | 3914961 | conjugal transfer protein TrbM |
|  |  |  | WP_012250601.1 |  | 3914973 | 3915638 | lytic transglycosylase domain-containing protein |
|  |  |  | WP_012250602.1 |  | 3915729 | 3916049 | TrbC/VirB2 family protein |
|  |  |  | WP_012250603.1 |  | 3916053 | 3916373 | VirB3 family type IV secretion system protein |
|  |  |  | WP_012250604.1 |  | 3916385 | 3918829 | VirB4 family type IV secretion/conjugal transfer ATPase |
|  |  |  | WP_012250607.1 |  | 3920011 | 3920715 | type IV secretion system protein |
|  |  |  | WP_012250608.1 |  | 3920725 | 3921675 | type IV secretion system protein |
|  |  |  | WP_012250610.1 |  | 3921904 | 3922590 | conjugal transfer protein TraJ |
|  |  |  | WP_012250611.1 |  | 3922618 | 3923505 | TrbG/VirB9 family P-type conjugative transfer protein |
|  |  |  | WP_012250612.1 | virB10 | 3923502 | 3924635 | type IV secretion system protein VirB10 |
|  |  |  | WP_012250613.1 | virB11 | 3924613 | 3925632 | P-type DNA transfer ATPase VirB11 |
|  |  |  | WP_012250618.1 |  | 3929234 | 3930880 | type IV secretion system DNA-binding domain-containing protein |
| 3935068 | 3945118 | 10050 | WP_050978259.1 |  | 3935068 | 3935682 | GntR family transcriptional regulator |
|  |  |  | WP_012250621.1 | gorA | 3935987 | 3937357 | glutathione-disulfide reductase |
|  |  |  | WP_012250622.1 |  | 3937357 | 3938016 | glutathione S-transferase N-terminal domain-containing protein |
|  |  |  | WP_012250623.1 |  | 3938057 | 3938671 | glutathione S-transferase family protein |
|  |  |  | WP_012250624.1 |  | 3938668 | 3939300 | glutathione S-transferase N-terminal domain-containing protein |
|  |  |  | WP_050978261.1 |  | 3939676 | 3939993 | MarR family transcriptional regulator |
|  |  |  | WP_012250627.1 |  | 3940343 | 3941416 | maleylacetate reductase |
|  |  |  | WP_012250628.1 |  | 3941416 | 3941949 | enolase |
|  |  |  | WP_085970224.1 |  | 3941962 | 3943523 | IS3 family transposase |
|  |  |  | WP_012250631.1 |  | 3943566 | 3944231 | muconate cycloisomerase |
|  |  |  | WP_012250632.1 |  | 3944228 | 3944929 | dienelactone hydrolase family protein |
| 3944228 | 3964968 | 20740 | WP_012250631.1 |  | 3943566 | 3944231 | muconate cycloisomerase |
|  |  |  | WP_012250632.1 |  | 3944228 | 3944929 | dienelactone hydrolase family protein |
|  |  |  | WP_012250633.1 |  | 3945315 | 3946382 | IS630-like element IS1066 family transposase |
|  |  |  | WP_012250634.1 |  | 3946637 | 3947467 | alpha/beta fold hydrolase |
|  |  |  | WP_041863076.1 |  | 3947553 | 3948905 | Rieske 2Fe-2S domain-containing protein |
|  |  |  | WP_012250636.1 |  | 3949015 | 3949578 | 3-phenylpropionate/cinnamic acid dioxygenase subunit beta |
|  |  |  | WP_012250637.1 |  | 3949587 | 3949910 | non-heme iron oxygenase ferredoxin subunit |
|  |  |  | WP_012250638.1 |  | 3949910 | 3951142 | oxidoreductase |
|  |  |  | WP_012250639.1 | bphB | 3951139 | 3951966 | cis-2,3-dihydrobiphenyl-2,3-diol dehydrogenase |
|  |  |  | WP_081483019.1 |  | 3951967 | 3953154 | Rieske 2Fe-2S domain-containing protein |
|  |  |  | WP_012250641.1 |  | 3953141 | 3953626 | nuclear transport factor 2 family protein |
|  |  |  | WP_151209069.1 |  | 3953744 | 3955336 | MFS transporter |
|  |  |  | WP_158310089.1 |  | 3955495 | 3956445 | helix-turn-helix domain-containing protein |
|  |  |  | WP_012250644.1 |  | 3956647 | 3957531 | LysR family transcriptional regulator |
|  |  |  | WP_012250645.1 |  | 3957681 | 3958436 | chlorocatechol 1,2-dioxygenase |
|  |  |  | WP_011255151.1 |  | 3958433 | 3959545 | muconate cycloisomerase family protein |
|  |  |  | WP_158310090.1 |  | 3959682 | 3960557 | tripartite tricarboxylate transporter substrate binding protein |
|  |  |  | WP_011255149.1 |  | 3960579 | 3961295 | dienelactone hydrolase family protein |
|  |  |  | WP_011255148.1 |  | 3961292 | 3962350 | maleylacetate reductase |
|  |  |  |  |  | 3962432 | 3963037 | AraC family transcriptional regulator |
|  |  |  | WP_012250647.1 |  | 3963312 | 3963491 | AraC family transcriptional regulator |
|  |  |  | WP_196491415.1 |  | 3963488 | 3964189 | ATP-binding cassette domain-containing protein |
|  |  |  | WP_012250649.1 |  | 3964207 | 3964968 | ABC transporter ATP-binding protein |
|  |  |  | WP_050978265.1 |  | 3964965 | 3965978 | branched-chain amino acid ABC transporter permease |
| 4423618 | 4430698 | 7080 | WP_012251067.1 |  | 4423618 | 4423887 | WXG100 family type VII secretion target |
|  |  |  | WP_012251068.1 |  | 4424048 | 4425028 | IS5 family transposase |
|  |  |  | WP_012251069.1 |  | 4425176 | 4425814 | CoA transferase subunit B |
|  |  |  | WP_012251070.1 |  | 4425859 | 4427028 | acetyl-CoA C-acetyltransferase |
|  |  |  |  |  | 4427191 | 4427957 | IS5 family transposase |
|  |  |  | WP_012251073.1 |  | 4428521 | 4429150 | TetR family transcriptional regulator |
|  |  |  | WP_012251074.1 |  | 4429152 | 4429826 | TetR/AcrR family transcriptional regulator |
|  |  |  | WP_012251075.1 |  | 4429847 | 4430698 | universal stress protein |
| 4443235 | 4574105 | 130870 | WP_012251087.1 |  | 4443235 | 4444233 | aldo/keto reductase |
|  |  |  | WP_012251088.1 |  | 4444256 | 4445074 | aldo/keto reductase |
|  |  |  |  |  | 4445138 | 4445904 | IS5 family transposase |
|  |  |  |  | traD | 4446039 | 4447851 | type IV conjugative transfer system coupling protein TraD |
|  |  |  |  |  | 4447856 | 4448011 | integrating conjugative element protein |
|  |  |  |  |  | 4448009 | 4448401 | lytic transglycosylase domain-containing protein |
|  |  |  |  |  | 4448383 | 4448757 | TIGR03759 family integrating conjugative element protein |
|  |  |  | WP_041863140.1 |  | 4448745 | 4450028 | FAD-dependent oxidoreductase |
|  |  |  |  |  | 4450350 | 4451134 | ATP-binding cassette domain-containing protein |
|  |  |  | WP_108627534.1 |  | 4451137 | 4452825 | cyclic peptide export ABC transporter |
|  |  |  | WP_012251098.1 | fhuB | 4452834 | 4454837 | Fe(3+)-hydroxamate ABC transporter permease FhuB |
|  |  |  | WP_012251099.1 |  | 4454840 | 4455850 | ABC transporter substrate-binding protein |
|  |  |  | WP_012251100.1 |  | 4455865 | 4458099 | TonB-dependent receptor |
|  |  |  | WP_041863143.1 |  | 4458275 | 4459066 | helix-turn-helix transcriptional regulator |
|  |  |  | WP_197535829.1 |  | 4459087 | 4459983 | AMP-binding protein |
|  |  |  | WP_050978273.1 |  | 4460020 | 4462053 | AMP-binding protein |
|  |  |  | WP_012251102.1 |  | 4462263 | 4462775 | single-stranded DNA-binding protein |
|  |  |  |  |  | 4462852 | 4463376 | DUF3158 family protein |
|  |  |  |  |  | 4463373 | 4463594 | DUF1845 family protein |
|  |  |  |  |  | 4463589 | 4465010 | ParB family protein |
|  |  |  | WP_012251105.1 |  | 4465247 | 4465990 | ParA family protein |
|  |  |  | WP_197535830.1 |  | 4466024 | 4466134 | AlpA family phage regulatory protein |
|  |  |  | WP_102773543.1 |  | 4466250 | 4466879 | TetR/AcrR family transcriptional regulator |
|  |  |  | WP_012251108.1 |  | 4467479 | 4468015 | TetR family transcriptional regulator |
|  |  |  | WP_041863145.1 |  | 4468177 | 4469172 | polyprenyl synthetase family protein |
|  |  |  | WP_041864336.1 |  | 4469546 | 4469962 | MarR family transcriptional regulator |
|  |  |  | WP_012251111.1 |  | 4470620 | 4471867 | multidrug effflux MFS transporter |
|  |  |  | WP_158310095.1 |  | 4472393 | 4473142 | helix-turn-helix domain-containing protein |
|  |  |  | WP_012251113.1 |  | 4473552 | 4474169 | LysR family transcriptional regulator |
|  |  |  | WP_012251114.1 |  | 4474277 | 4474561 | helix-turn-helix transcriptional regulator |
|  |  |  | WP_012251115.1 |  | 4474588 | 4475481 | LysR family transcriptional regulator |
|  |  |  |  |  | 4475879 | 4476053 | prepilin-type N-terminal cleavage/methylation domain-containing protein |
|  |  |  | WP_012251116.1 |  | 4476812 | 4477657 | universal stress protein |
|  |  |  | WP_012251117.1 |  | 4477751 | 4479223 | efflux transporter outer membrane subunit |
|  |  |  |  |  | 4479241 | 4479864 | efflux RND transporter permease subunit |
|  |  |  | WP_012206014.1 |  | 4480017 | 4480658 | substrate-binding domain-containing protein |
|  |  |  | WP_012251119.1 |  | 4480780 | 4481085 | helix-turn-helix transcriptional regulator |
|  |  |  | WP_012206012.1 |  | 4481123 | 4482013 | LysR family transcriptional regulator |
|  |  |  | WP_012206011.1 |  | 4482293 | 4483201 | WYL domain-containing protein |
|  |  |  | WP_012251120.1 |  | 4483214 | 4486447 | DEAD/DEAH box helicase family protein |
|  |  |  | WP_012251121.1 |  | 4486444 | 4487259 | DUF4391 domain-containing protein |
|  |  |  | WP_012251122.1 |  | 4487301 | 4489199 | site-specific DNA-methyltransferase |
|  |  |  | WP_012251123.1 |  | 4489211 | 4492216 | DEAD/DEAH box helicase family protein |
|  |  |  | WP_012251124.1 |  | 4492297 | 4493031 | VWA domain-containing protein |
|  |  |  | WP_012251067.1 |  | 4493031 | 4493300 | WXG100 family type VII secretion target |
|  |  |  |  |  | 4494253 | 4495805 | IS3 family transposase |
|  |  |  | WP_197535831.1 |  | 4496841 | 4499525 | DUF87 domain-containing protein |
|  |  |  | WP_012251130.1 |  | 4499522 | 4501285 | DUF1887 family protein |
|  |  |  | WP_012206000.1 |  | 4501767 | 4502483 | protein phosphatase 2C domain-containing protein |
|  |  |  | WP_012205999.1 |  | 4502476 | 4503819 | serine/threonine protein kinase |
|  |  |  | WP_012251131.1 |  | 4503816 | 4507196 | AAA family ATPase |
|  |  |  | WP_008265595.1 |  | 4509133 | 4511769 | chromosome segregation ATPase |
|  |  |  | WP_041863150.1 |  | 4511766 | 4515101 | cold shock domain-containing protein |
|  |  |  | WP_012205994.1 |  | 4515132 | 4516985 | TraI domain-containing protein |
|  |  |  | WP_008265574.1 |  | 4517283 | 4517600 | type II toxin-antitoxin system PrlF family antitoxin |
|  |  |  | WP_012205993.1 |  | 4517600 | 4518058 | type II toxin-antitoxin system YhaV family toxin |
|  |  |  | WP_008264713.1 |  | 4518086 | 4518445 | DUF3742 family protein |
|  |  |  | WP_008266205.1 |  | 4518452 | 4519975 | conjugal transfer protein TraG |
|  |  |  | WP_012205992.1 |  | 4520359 | 4521765 | integrating conjugative element protein |
|  |  |  | WP_012205991.1 |  | 4521776 | 4522723 | TIGR03756 family integrating conjugative element protein |
|  |  |  | WP_008266034.1 |  | 4522720 | 4523166 | TIGR03757 family integrating conjugative element protein |
|  |  |  | WP_012205990.1 | radC | 4523331 | 4523825 | DNA repair protein RadC |
|  |  |  | WP_014596091.1 |  | 4524004 | 4524768 | DsbA family protein |
|  |  |  | WP_012205988.1 |  | 4524784 | 4527693 | conjugative transfer ATPase |
|  |  |  | WP_012251134.1 |  | 4527693 | 4528142 | TIGR03751 family conjugal transfer lipoprotein |
|  |  |  | WP_012251135.1 |  | 4528123 | 4529532 | TIGR03752 family integrating conjugative element protein |
|  |  |  | WP_033945092.1 |  | 4529522 | 4530451 | TIGR03749 family integrating conjugative element protein |
|  |  |  | WP_012205984.1 |  | 4530457 | 4531149 | TIGR03746 family integrating conjugative element protein |
|  |  |  | WP_012205983.1 |  | 4531146 | 4531556 | TIGR03750 family conjugal transfer protein |
|  |  |  | WP_012205982.1 |  | 4531570 | 4531929 | TIGR03745 family integrating conjugative element membrane protein |
|  |  |  | WP_012205981.1 |  | 4531946 | 4532179 | TIGR03758 family integrating conjugative element protein |
|  |  |  | WP_012251136.1 |  | 4532176 | 4532559 | RAQPRD family integrative conjugative element protein |
|  |  |  | WP_012205979.1 |  | 4532658 | 4533419 | TIGR03747 family integrating conjugative element membrane protein |
|  |  |  |  | traD | 4533416 | 4535600 | type IV conjugative transfer system coupling protein TraD |
|  |  |  | WP_012205977.1 |  | 4535605 | 4536153 | integrating conjugative element protein |
|  |  |  | WP_012205976.1 |  | 4536150 | 4536755 | transglycosylase SLT domain-containing protein |
|  |  |  | WP_012205975.1 |  | 4536737 | 4537474 | TIGR03759 family integrating conjugative element protein |
|  |  |  | WP_012205973.1 |  | 4538127 | 4538705 | PilL N-terminal domain-containing protein |
|  |  |  | WP_081483032.1 |  | 4538848 | 4540569 | ATP-dependent helicase |
|  |  |  | WP_012205971.1 |  | 4540566 | 4542368 | AAA family ATPase |
|  |  |  |  |  | 4542541 | 4544502 | DEAD/DEAH box helicase |
|  |  |  |  |  | 4547040 | 4547357 | DEAD/DEAH box helicase |
|  |  |  | WP_041863154.1 |  | 4548282 | 4549370 | SAM-dependent methyltransferase |
|  |  |  | WP_012248065.1 | ltrA | 4549438 | 4551153 | group II intron reverse transcriptase/maturase |
|  |  |  | WP_085970191.1 |  | 4551433 | 4552607 | IS3 family transposase |
|  |  |  | WP_012248063.1 | ltrA | 4554103 | 4555821 | group II intron reverse transcriptase/maturase |
|  |  |  | WP_012205968.1 |  | 4556817 | 4557515 | DUF3275 family protein |
|  |  |  | WP_012205967.1 |  | 4557611 | 4558438 | DUF945 domain-containing protein |
|  |  |  | WP_012205966.1 |  | 4558589 | 4559503 | DUF3577 domain-containing protein |
|  |  |  | WP_012205960.1 |  | 4564022 | 4566034 | DNA topoisomerase III |
|  |  |  | WP_012205959.1 |  | 4566304 | 4566744 | single-stranded DNA-binding protein |
|  |  |  | WP_012205958.1 |  | 4566818 | 4567345 | DUF3158 family protein |
|  |  |  | WP_012205957.1 |  | 4567342 | 4568142 | TIGR03761 family integrating conjugative element protein |
|  |  |  | WP_008267477.1 |  | 4569708 | 4570268 | DUF2857 domain-containing protein |
|  |  |  | WP_012205955.1 |  | 4570284 | 4571876 | ParB family protein |
|  |  |  | WP_012251147.1 |  | 4572101 | 4572976 | ParA family protein |
|  |  |  | WP_012205952.1 |  | 4573019 | 4573231 | AlpA family transcriptional regulator |
| 4492297 | 4515101 | 22804 | WP_012251124.1 |  | 4492297 | 4493031 | VWA domain-containing protein |
|  |  |  | WP_012251067.1 |  | 4493031 | 4493300 | WXG100 family type VII secretion target |
|  |  |  |  |  | 4494253 | 4495805 | IS3 family transposase |
|  |  |  | WP_197535831.1 |  | 4496841 | 4499525 | DUF87 domain-containing protein |
|  |  |  | WP_012251130.1 |  | 4499522 | 4501285 | DUF1887 family protein |
|  |  |  | WP_012206000.1 |  | 4501767 | 4502483 | protein phosphatase 2C domain-containing protein |
|  |  |  | WP_012205999.1 |  | 4502476 | 4503819 | serine/threonine protein kinase |
|  |  |  | WP_012251131.1 |  | 4503816 | 4507196 | AAA family ATPase |
|  |  |  | WP_008265595.1 |  | 4509133 | 4511769 | chromosome segregation ATPase |
|  |  |  | WP_041863150.1 |  | 4511766 | 4515101 | cold shock domain-containing protein |
| 4652954 | 4665843 | 12889 | WP_012251230.1 |  | 4654626 | 4654958 | putative holin |
|  |  |  | WP_197535852.1 |  | 4654987 | 4655592 | transglycosylase SLT domain-containing protein |
|  |  |  | WP_085970278.1 |  | 4656339 | 4656494 | TraR/DksA C4-type zinc finger protein |
|  |  |  | WP_012251235.1 |  | 4656494 | 4656835 | DUF2730 family protein |
|  |  |  | WP_012251237.1 |  | 4657140 | 4657688 | DUF3486 family protein |
|  |  |  | WP_012251239.1 |  | 4659407 | 4660882 | DUF935 family protein |
|  |  |  | WP_012251240.1 |  | 4660883 | 4661677 | F protein (gpF) (protein gp30) |
|  |  |  | WP_012251241.1 |  | 4661682 | 4662194 | phage virion morphogenesis protein |
|  |  |  | WP_012251243.1 |  | 4663580 | 4663924 | DUF2190 family protein |
|  |  |  | WP_012251244.1 |  | 4663949 | 4664884 | major capsid protein |
|  |  |  | WP_041863171.1 |  | 4665433 | 4665843 | DUF1320 family protein |
| 4674340 | 4679794 | 5454 | WP_012251258.1 |  | 4674340 | 4675392 | baseplate J/gp47 family protein |
|  |  |  | WP_012251259.1 |  | 4675377 | 4676003 | DUF2313 domain-containing protein |
|  |  |  | WP_081483074.1 |  | 4677899 | 4678090 | Com family DNA-binding transcriptional regulator |
|  |  |  | WP_197535833.1 |  | 4678062 | 4678805 | site-specific DNA-methyltransferase |
|  |  |  | WP_012251263.1 |  | 4678808 | 4679794 | site-specific integrase |
| 4745033 | 4762613 | 17580 | WP_012251320.1 |  | 4745033 | 4745905 | branched-chain amino acid ABC transporter permease |
|  |  |  | WP_012251321.1 |  | 4745914 | 4746852 | branched-chain amino acid ABC transporter permease |
|  |  |  | WP_012251322.1 |  | 4746849 | 4747607 | ABC transporter ATP-binding protein |
|  |  |  | WP_012251323.1 |  | 4747594 | 4748319 | ABC transporter ATP-binding protein |
|  |  |  | WP_197535853.1 |  | 4756515 | 4756628 | SEC-C domain-containing protein |
|  |  |  | WP_012251328.1 |  | 4757212 | 4757688 | anion permease |
|  |  |  | WP_012251331.1 |  | 4759238 | 4759852 | tyrosine-type recombinase/integrase |
|  |  |  | WP_041863183.1 |  | 4760087 | 4760716 | LysE family translocator |
|  |  |  | WP_012251333.1 |  | 4760916 | 4761599 | glutathione S-transferase family protein |
|  |  |  | WP_012251334.1 |  | 4761849 | 4762613 | phytanoyl-CoA dioxygenase family protein |
| 4748664 | 4761599 | 12935 | WP_197535853.1 |  | 4756515 | 4756628 | SEC-C domain-containing protein |
|  |  |  | WP_012251328.1 |  | 4757212 | 4757688 | anion permease |
|  |  |  | WP_012251331.1 |  | 4759238 | 4759852 | tyrosine-type recombinase/integrase |
|  |  |  | WP_041863183.1 |  | 4760087 | 4760716 | LysE family translocator |
|  |  |  | WP_012251333.1 |  | 4760916 | 4761599 | glutathione S-transferase family protein |
| 4804647 | 4813426 | 8779 | WP_012251373.1 |  | 4804647 | 4806005 | integrase arm-type DNA-binding domain-containing protein |
|  |  |  | WP_012251374.1 |  | 4805980 | 4806240 | AlpA family phage regulatory protein |
|  |  |  | WP_041863196.1 |  | 4806444 | 4806749 | BrnT family toxin |
|  |  |  | WP_012251376.1 |  | 4806730 | 4807038 | BrnA antitoxin family protein |
|  |  |  | WP_012251377.1 |  | 4807704 | 4808117 | antirestriction protein |
|  |  |  | WP_151208989.1 | arsH | 4808163 | 4808870 | arsenical resistance protein ArsH |
|  |  |  | WP_012251379.1 | arsC | 4808881 | 4809303 | arsenate reductase (glutaredoxin) |
|  |  |  | WP_012251380.1 | arsB | 4809316 | 4810398 | ACR3 family arsenite efflux transporter |
|  |  |  | WP_012251381.1 |  | 4810406 | 4810903 | arsenate reductase ArsC |
|  |  |  | WP_041863198.1 |  | 4810908 | 4811231 | helix-turn-helix transcriptional regulator |
|  |  |  | WP_081483037.1 |  | 4811580 | 4811972 | ATPase |
|  |  |  | WP_012251385.1 |  | 4812902 | 4813426 | DNA repair protein RadC |
| 4816993 | 4823466 | 6473 | WP_012251389.1 |  | 4819128 | 4819427 | helix-turn-helix transcriptional regulator |
|  |  |  | WP_012251390.1 |  | 4819699 | 4820046 | DUF2958 domain-containing protein |
|  |  |  | WP_012251391.1 |  | 4820160 | 4820957 | DUF2285 domain-containing protein |
|  |  |  | WP_012251392.1 |  | 4821093 | 4821377 | helix-turn-helix domain-containing protein |
|  |  |  | WP_012251393.1 |  | 4821405 | 4822289 | replication initiator protein A |
|  |  |  | WP_012251394.1 |  | 4822280 | 4823236 | AAA family ATPase |
| 4826942 | 4876400 | 49458 | WP_012251398.1 |  | 4827381 | 4828841 | multicopper oxidase domain-containing protein |
|  |  |  | WP_012251399.1 |  | 4829093 | 4831411 | copper-translocating P-type ATPase |
|  |  |  | WP_012251400.1 |  | 4832089 | 4833330 | TolC family protein |
|  |  |  | WP_151209081.1 |  | 4833366 | 4834832 | efflux RND transporter periplasmic adaptor subunit |
|  |  |  | WP_012251402.1 |  | 4834829 | 4837996 | efflux RND transporter permease subunit |
|  |  |  | WP_197535854.1 |  | 4838058 | 4838384 | copper-binding protein |
|  |  |  | WP_085970280.1 |  | 4838955 | 4839233 | DUF4148 domain-containing protein |
|  |  |  | WP_012251407.1 |  | 4839832 | 4840086 | DUF2933 domain-containing protein |
|  |  |  | WP_012251408.1 |  | 4840287 | 4841474 | thioredoxin family protein |
|  |  |  | WP_012251410.1 |  | 4842098 | 4842532 | metal-binding protein |
|  |  |  | WP_012251411.1 |  | 4842564 | 4843004 | cytochrome c |
|  |  |  | WP_012251413.1 | copD | 4843636 | 4844553 | copper homeostasis membrane protein CopD |
|  |  |  | WP_085970230.1 |  | 4844570 | 4845657 | IS3 family transposase |
|  |  |  | WP_012251414.1 | copC | 4845763 | 4846149 | copper homeostasis periplasmic binding protein CopC |
|  |  |  | WP_197535855.1 |  | 4846588 | 4847109 | DUF305 domain-containing protein |
|  |  |  | WP_041864399.1 |  | 4847633 | 4848490 | copper resistance protein B |
|  |  |  | WP_012251418.1 |  | 4848749 | 4850608 | copper resistance system multicopper oxidase |
|  |  |  | WP_012251419.1 |  | 4850656 | 4851159 | cupredoxin family protein |
|  |  |  | WP_012251420.1 |  | 4851236 | 4852618 | heavy metal sensor histidine kinase |
|  |  |  | WP_012251421.1 |  | 4852615 | 4853289 | heavy metal response regulator transcription factor |
|  |  |  | WP_012251422.1 |  | 4853954 | 4854337 | DUF4148 domain-containing protein |
|  |  |  | WP_012251423.1 |  | 4854456 | 4855568 | glycosyltransferase |
|  |  |  | WP_151209082.1 |  | 4856549 | 4856797 | DUF4148 domain-containing protein |
|  |  |  | WP_012251425.1 |  | 4856955 | 4857566 | cation transporter |
|  |  |  | WP_012251426.1 |  | 4857711 | 4858298 | ChbG/HpnK family deacetylase |
|  |  |  |  |  | 4858405 | 4859957 | IS3 family transposase |
|  |  |  | WP_158310100.1 |  | 4859943 | 4860146 | ChbG/HpnK family deacetylase |
|  |  |  | WP_012251427.1 |  | 4860222 | 4860617 | GtrA family protein |
|  |  |  | WP_012251428.1 |  | 4860673 | 4862130 | glycosyltransferase family 39 protein |
|  |  |  | WP_012251429.1 |  | 4862117 | 4863154 | glycosyltransferase family 2 protein |
|  |  |  | WP_012251430.1 |  | 4863336 | 4863671 | DUF190 domain-containing protein |
|  |  |  | WP_012251431.1 |  | 4863668 | 4866925 | CusA/CzcA family heavy metal efflux RND transporter |
|  |  |  | WP_012251432.1 |  | 4866928 | 4868091 | efflux RND transporter periplasmic adaptor subunit |
|  |  |  | WP_041863221.1 |  | 4868326 | 4869573 | TolC family protein |
|  |  |  | WP_041864401.1 |  | 4869856 | 4870545 | heavy metal response regulator transcription factor |
|  |  |  | WP_012251435.1 |  | 4870545 | 4871891 | heavy metal sensor histidine kinase |
|  |  |  | WP_041863222.1 |  | 4872148 | 4872834 | heavy metal response regulator transcription factor |
|  |  |  | WP_012251437.1 |  | 4873063 | 4874220 | porin |
|  |  |  | WP_012251438.1 |  | 4874795 | 4875100 | DUF4148 domain-containing protein |
|  |  |  | WP_012251439.1 |  | 4875471 | 4876400 | LysR family transcriptional regulator |
| 4844570 | 4879242 | 34672 | WP_085970230.1 |  | 4844570 | 4845657 | IS3 family transposase |
|  |  |  | WP_012251414.1 | copC | 4845763 | 4846149 | copper homeostasis periplasmic binding protein CopC |
|  |  |  | WP_197535855.1 |  | 4846588 | 4847109 | DUF305 domain-containing protein |
|  |  |  | WP_041864399.1 |  | 4847633 | 4848490 | copper resistance protein B |
|  |  |  | WP_012251418.1 |  | 4848749 | 4850608 | copper resistance system multicopper oxidase |
|  |  |  | WP_012251419.1 |  | 4850656 | 4851159 | cupredoxin family protein |
|  |  |  | WP_012251420.1 |  | 4851236 | 4852618 | heavy metal sensor histidine kinase |
|  |  |  | WP_012251421.1 |  | 4852615 | 4853289 | heavy metal response regulator transcription factor |
|  |  |  | WP_012251422.1 |  | 4853954 | 4854337 | DUF4148 domain-containing protein |
|  |  |  | WP_012251423.1 |  | 4854456 | 4855568 | glycosyltransferase |
|  |  |  | WP_151209082.1 |  | 4856549 | 4856797 | DUF4148 domain-containing protein |
|  |  |  | WP_012251425.1 |  | 4856955 | 4857566 | cation transporter |
|  |  |  | WP_012251426.1 |  | 4857711 | 4858298 | ChbG/HpnK family deacetylase |
|  |  |  |  |  | 4858405 | 4859957 | IS3 family transposase |
|  |  |  | WP_158310100.1 |  | 4859943 | 4860146 | ChbG/HpnK family deacetylase |
|  |  |  | WP_012251427.1 |  | 4860222 | 4860617 | GtrA family protein |
|  |  |  | WP_012251428.1 |  | 4860673 | 4862130 | glycosyltransferase family 39 protein |
|  |  |  | WP_012251429.1 |  | 4862117 | 4863154 | glycosyltransferase family 2 protein |
|  |  |  | WP_012251430.1 |  | 4863336 | 4863671 | DUF190 domain-containing protein |
|  |  |  | WP_012251431.1 |  | 4863668 | 4866925 | CusA/CzcA family heavy metal efflux RND transporter |
|  |  |  | WP_012251432.1 |  | 4866928 | 4868091 | efflux RND transporter periplasmic adaptor subunit |
|  |  |  | WP_041863221.1 |  | 4868326 | 4869573 | TolC family protein |
|  |  |  | WP_041864401.1 |  | 4869856 | 4870545 | heavy metal response regulator transcription factor |
|  |  |  | WP_012251435.1 |  | 4870545 | 4871891 | heavy metal sensor histidine kinase |
|  |  |  | WP_041863222.1 |  | 4872148 | 4872834 | heavy metal response regulator transcription factor |
|  |  |  | WP_012251437.1 |  | 4873063 | 4874220 | porin |
|  |  |  | WP_012251438.1 |  | 4874795 | 4875100 | DUF4148 domain-containing protein |
|  |  |  | WP_012251439.1 |  | 4875471 | 4876400 | LysR family transcriptional regulator |
|  |  |  | WP_012251440.1 |  | 4876733 | 4878742 | conjugal transfer protein TraG |
| 5093910 | 5102940 | 9030 | WP_012251651.1 |  | 5093910 | 5095142 | glycosyltransferase |
|  |  |  | WP_012251653.1 | wecB | 5096227 | 5097309 | UDP-N-acetylglucosamine 2-epimerase (non-hydrolyzing) |
|  |  |  | WP_012251654.1 |  | 5097309 | 5098574 | glycosyltransferase family 4 protein |
|  |  |  | WP_012251655.1 |  | 5098571 | 5099827 | glycosyltransferase |
|  |  |  | WP_151208998.1 |  | 5101144 | 5102940 | ABC transporter ATP-binding protein |
