# Supplementary material for: Genomic, morphological, and biochemical analyses of a multi-metal resistant but multi-drug susceptible strain of Bordetella petrii from hospital soil
Source: Sci Rep. 2022 May 19;12:8439. doi: 10.1038/s41598-022-12435-7 (PMC9120033; doi:10.1038/s41598-022-12435-7)
Supplement: Supplementary file 3 — Supplementary Information 3. [file 41598_2022_12435_MOESM3_ESM.docx]

**Supplementary File 3.** Specialty genes present within different species of *Bordetella petrii*

Specialty genes related to virulence factor, antibiotic resistance, transporter, and drug target were annotated from PATRIC server

| **Transporter** | | | | | |
| --- | --- | --- | --- | --- | --- |
| **Genome Name** | **Product** | **Function** | **Gene** | **Source ID** | **Source Organism** |
| *B*. *petrii*  DSM 12804 | Nitric-oxide reductase subunit C (EC 1.7.99.7) | the proton-translocating cytochrome oxidase (cox) superfamily. | norC | Q59646 | Pseudomonas aeruginosa ATCC 15692 |
|  | Hemin ABC transporter, permease protein | the atp-binding cassette (abc) superfamily. |  | Q7W024 | Bordetella pertussis |
|  | Nitric-oxide reductase subunit B (EC 1.7.99.7) | the proton-translocating cytochrome oxidase (cox) superfamily. | norB | P98008 | Pseudomonas stutzeri |
|  | Flagellar biosynthesis protein FlhA | the type iii (virulence-related) secretory pathway (iiisp) family. | flhA1 | P40729 | Salmonella typhimurium |
|  | Zinc transporter, ZIP family | the zinc (zn(2+))-iron (fe(2+)) permease (zip) family. |  | Q2KXZ6 | Bordetella avium 197N |
|  | Phosphate ABC transporter, ATP-binding protein PstB (TC 3.A.1.7.1) | the atp-binding cassette (abc) superfamily. | pstB | P0AAH0 | Escherichia coli |
|  | NADH-ubiquinone oxidoreductase chain B (EC 1.6.5.3) | the h(+) or na(+)-translocating nadh dehydrogenase (ndh) family. | nuoB | Q42577 | Arabidopsis thaliana |
|  | Uncharacterized MFS-type transporter | the major facilitator superfamily (mfs). |  | Q7W0Q7 | Bordetella pertussis |
|  | ABC transporter, ATP-binding protein (cluster 3, basic aa/glutamine/opines) | the atp-binding cassette (abc) superfamily. |  | Q52815 | Rhizobium leguminosarum |
| *B. petrii*  J49 | Phosphate ABC transporter, ATP-binding protein PstB (TC 3.A.1.7.1) | the atp-binding cassette (abc) superfamily. |  | P0AAH0 | Escherichia coli |
|  | Glycerol ABC transporter, permease protein GlpQ | the atp-binding cassette (abc) superfamily. |  | G3LHZ1 | Rhizobium leguminosarum |
|  | Hemin ABC transporter, permease protein | the atp-binding cassette (abc) superfamily. |  | Q7W024 | Bordetella pertussis |
|  | NADH-ubiquinone oxidoreductase chain B (EC 1.6.5.3) | the h(+) or na(+)-translocating nadh dehydrogenase (ndh) family. |  | Q42577 | Arabidopsis thaliana |
|  | Zinc transporter, ZIP family | the zinc (zn(2+))-iron (fe(2+)) permease (zip) family. |  | Q2KXZ6 | Bordetella avium 197N |
|  | ABC transporter, ATP-binding protein (cluster 3, basic aa/glutamine/opines) | the atp-binding cassette (abc) superfamily. |  | Q52815 | Rhizobium leguminosarum |
|  | Nitric-oxide reductase subunit B (EC 1.7.99.7) | the proton-translocating cytochrome oxidase (cox) superfamily. |  | P98008 | Pseudomonas stutzeri |
|  | Uncharacterized MFS-type transporter | the major facilitator superfamily (mfs). |  | Q7W0Q7 | Bordetella pertussis |
| *B. petrii*  J51 | Uncharacterized MFS-type transporter | the major facilitator superfamily (mfs). |  | Q7W0Q7 | Bordetella pertussis |
|  | NADH-ubiquinone oxidoreductase chain B (EC 1.6.5.3) | the h(+) or na(+)-translocating nadh dehydrogenase (ndh) family. |  | Q42577 | Arabidopsis thaliana |
|  | Hemin ABC transporter, permease protein | the atp-binding cassette (abc) superfamily. |  | Q7W024 | Bordetella pertussis |
|  | Nitric-oxide reductase subunit B (EC 1.7.99.7) | the proton-translocating cytochrome oxidase (cox) superfamily. |  | P98008 | Pseudomonas stutzeri |
|  | Lead, cadmium, zinc and mercury transporting ATPase (EC 3.6.3.3) (EC 3.6.3.5); Copper-translocating P-type ATPase (EC 3.6.3.4) | the p-type atpase (p-atpase) superfamily. |  | Q8ZRG7 | Salmonella typhimurium |
|  | Zinc transporter, ZIP family | the zinc (zn(2+))-iron (fe(2+)) permease (zip) family. |  | Q2KXZ6 | Bordetella avium 197N |
|  | Phosphate ABC transporter, ATP-binding protein PstB (TC 3.A.1.7.1) | the atp-binding cassette (abc) superfamily. |  | P0AAH0 | Escherichia coli |
|  | ABC transporter, ATP-binding protein (cluster 3, basic aa/glutamine/opines) | the atp-binding cassette (abc) superfamily. |  | Q52815 | Rhizobium leguminosarum |
| *B*. *petrii* BMC_SI_3 | Phosphate ABC transporter, ATP-binding protein PstB (TC 3.A.1.7.1) | the atp-binding cassette (abc) superfamily. |  | P0AAH0 | *Escherichia coli* |
|  | Uncharacterized MFS-type transporter | the major facilitator superfamily (mfs). |  | Q7W0Q7 | *Bordetella pertussis* |
|  | NADH-ubiquinone oxidoreductase chain B (EC 1.6.5.3) | the h(+) or na(+)-translocating nadh dehydrogenase (ndh) family. |  | Q42577 | *Arabidopsis thaliana* (Mouse-ear cress) |
|  | Hemin ABC transporter, permease protein | the atp-binding cassette (abc) superfamily. |  | Q7W024 | *Bordetella pertussis* |
|  | Nitric-oxide reductase subunit B (EC 1.7.99.7) | the proton-translocating cytochrome oxidase (cox) superfamily. |  | P98008 | *Pseudomonas stutzeri* |
|  | TRAP dicarboxylate transporter, DctM subunit, unknown substrate 6 | the tripartite atp-independent periplasmic transporter (trap-t) family. |  | D5ATK1 | *Rhodobacter capsulatus* (strain ATCC) |
|  | ABC transporter, ATP-binding protein (cluster 3, basic aa/glutamine/opines) | the atp-binding cassette (abc) superfamily. |  | Q52815 | *Rhizobium leguminosarum* |
|  | ATP synthase beta chain (EC 3.6.3.14) | the h(+)- or na(+)-translocating f-type, v-type and a-type atpase (f-atpase) superfamily. |  | P0ABB4 | *Escherichia coli* |
|  | Nitric-oxide reductase subunit C (EC 1.7.99.7) | the proton-translocating cytochrome oxidase (cox) superfamily. |  | Q59646 | *Pseudomonas aeruginosa* (strain ATCC 15692) |
|  | Zinc transporter, ZIP family | the zinc (zn(2+))-iron (fe(2+)) permease (zip) family. |  | Q2KXZ6 | *Bordetella avium* (strain 197N) |
| *B. petrii*  BT 1 9.2 | Hemin ABC transporter, permease protein | the atp-binding cassette (abc) superfamily. |  | Q7W024 | *Bordetella pertussis* |
|  | Nitric-oxide reductase subunit B (EC 1.7.99.7) | the proton-translocating cytochrome oxidase (cox) superfamily. |  | P98008 | *Pseudomonas stutzeri* |
|  | ABC transporter involved in cytochrome c biogenesis, CcmB subunit | the atp-binding cassette (abc) superfamily. | ccmB | K6BIH6 | *Pseudomonas viridiflava* UASWS0038 |
|  | Aquaporin Z | the major intrinsic protein (mip) family. | aqpZ | P60844 | *Escherichia coli* |
|  | NADH-ubiquinone oxidoreductase chain B (EC 1.6.5.3) | the h(+) or na(+)-translocating nadh dehydrogenase (ndh) family. |  | Q42577 | *Arabidopsis thaliana* |
|  | Lead, cadmium, zinc and mercury transporting ATPase (EC 3.6.3.3) (EC 3.6.3.5); Copper-translocating P-type ATPase (EC 3.6.3.4) | the p-type atpase (p-atpase) superfamily. |  | E3HXW5 | *Achromobacter xylosoxidans* (strain A8) |
|  | Phosphate ABC transporter, ATP-binding protein PstB (TC 3.A.1.7.1) | the atp-binding cassette (abc) superfamily. | pstB | P0AAH0 | *Escherichia coli* |
|  | Nitric-oxide reductase subunit C (EC 1.7.99.7) | the proton-translocating cytochrome oxidase (cox) superfamily. |  | Q59646 | *Pseudomonas aeruginosa* (strain ATCC 15692) |
|  | Zinc transporter, ZIP family | the zinc (zn(2+))-iron (fe(2+)) permease (zip) family. |  | Q2KXZ6 | *Bordetella avium* (strain 197N) |
|  | Cobalt/zinc/cadmium resistance protein CzcD | the cation diffusion facilitator (cdf) family. |  | E3HXW8 | *Achromobacter xylosoxidans* (strain A8) |
| **Antibiotic Resistance** | | | | | |
| **Genome Name** | **Product** | **Function** | **Gene** | **Source ID** | **Source Organism** |
| *Bordetella petrii* DSM 12804 | DNA-directed RNA polymerase beta' subunit (EC 2.7.7.6) |  | rpoC |  |  |
|  | SSU ribosomal protein S12p (S23e) |  | rpsL |  |  |
|  | 1-deoxy-D-xylulose 5-phosphate reductoisomerase (EC 1.1.1.267) |  | dxr |  |  |
|  | Multidrug efflux system MdtABC-TolC, inner-membrane proton/drug antiporter MdtB (RND type) |  |  |  |  |
|  | DNA gyrase subunit A (EC 5.99.1.3) |  | gyrA |  |  |
|  | Outer membrane factor (OMF) lipoprotein associated wth MdtABC efflux system |  |  |  |  |
|  | Transcription termination factor Rho |  | rho |  |  |
|  | 16S rRNA (guanine(527)-N(7))-methyltransferase (EC 2.1.1.170) |  | gidB |  |  |
|  | Dihydropteroate synthase (EC 2.5.1.15) |  | folP |  |  |
|  | 3-oxoacyl-[acyl-carrier-protein] synthase, KASII (EC 2.3.1.179) |  |  |  |  |
|  | Outer membrane factor (OMF) lipoprotein associated wth EmrAB-OMF efflux system |  |  |  |  |
|  | SSU ribosomal protein S10p (S20e) |  | rpsJ | YP_208874.1 | *Neisseria gonorrhoeae* FA 1090 |
|  | Alanine racemase (EC 5.1.1.1) |  | dadX |  |  |
|  | Glycerophosphoryl diester phosphodiesterase (EC 3.1.4.46) |  | ugpQ |  |  |
|  | 3-oxoacyl-[acyl-carrier-protein] synthase, KASII (EC 2.3.1.179) |  | fabF |  |  |
|  | Class C beta-lactamase (EC 3.5.2.6) |  | ampC |  |  |
|  | Multidrug efflux system EmrAB-OMF, inner-membrane proton/drug antiporter EmrB (MFS type) |  |  |  |  |
|  | CDP-diacylglycerol--glycerol-3-phosphate 3-phosphatidyltransferase (EC 2.7.8.5) |  | pgsA |  |  |
|  | Hydrogen peroxide-inducible genes activator => OxyR |  |  |  |  |
|  | Multidrug efflux system, inner membrane proton/drug antiporter (RND type) => MexB of MexAB-OprM |  |  |  |  |
|  | Translation elongation factor Tu |  | tuf2 |  |  |
|  | Translation elongation factor G |  | fusA2 |  |  |
|  | DNA gyrase subunit B (EC 5.99.1.3) |  | gyrB |  |  |
|  | SSU ribosomal protein S10p (S20e) |  | rpsJ |  |  |
|  | Dihydrofolate reductase (EC 1.5.1.3) |  | folA |  |  |
|  | LSU ribosomal protein L6p (L9e) |  | rplF |  |  |
|  | DNA-directed RNA polymerase beta subunit (EC 2.7.7.6) |  | rpoB |  |  |
|  | Isoleucyl-tRNA synthetase (EC 6.1.1.5) |  | ileS |  |  |
|  | Multidrug efflux system, outer membrane factor lipoprotein of OprM/OprM family |  | cusC1 |  |  |
|  | Multidrug efflux system MdtABC-TolC, membrane fusion component MdtA |  |  |  |  |
|  | Multidrug efflux system EmrAB-OMF, membrane fusion component EmrA |  |  |  |  |
|  | Lipid A 4'-phosphatase LpxF-like, putative |  |  |  |  |
|  | Enoyl-[acyl-carrier-protein] reductase [NADH] (EC 1.3.1.9) |  | fabI |  |  |
|  | Multidrug efflux system, membrane fusion component => MexA of MexAB-OprM |  | acrA |  |  |
|  | Multidrug efflux system MdtABC-TolC, inner-membrane proton/drug antiporter MdtC (RND type) |  |  |  |  |
|  | Translation elongation factor G |  | fusA1 |  |  |
|  | Translation elongation factor Tu |  | tuf1 |  |  |
|  | UDP-N-acetylglucosamine 1-carboxyvinyltransferase (EC 2.5.1.7) |  | murA |  |  |
|  | D-alanine--D-alanine ligase (EC 6.3.2.4) |  | ddl |  |  |
| *Bordetella petrii* J49 | Glycerophosphoryl diester phosphodiesterase (EC 3.1.4.46) |  |  |  |  |
|  | Translation elongation factor G |  |  |  |  |
|  | DNA-directed RNA polymerase beta' subunit (EC 2.7.7.6) |  |  |  |  |
|  | Class A beta-lactamase (EC 3.5.2.6) |  |  |  |  |
|  | Enoyl-[acyl-carrier-protein] reductase [NADH] (EC 1.3.1.9) |  |  |  |  |
|  | LSU ribosomal protein L6p (L9e) |  |  |  |  |
|  | Multidrug efflux system, membrane fusion component => MexA of MexAB-OprM |  |  |  |  |
|  | Translation elongation factor G |  |  |  |  |
|  | 1-deoxy-D-xylulose 5-phosphate reductoisomerase (EC 1.1.1.267) |  |  |  |  |
|  | Alanine racemase (EC 5.1.1.1) |  |  |  |  |
|  | Multidrug efflux system MdtABC-TolC, inner-membrane proton/drug antiporter MdtC (RND type) |  |  |  |  |
|  | 3-oxoacyl-[acyl-carrier-protein] synthase, KASII (EC 2.3.1.179) |  |  |  |  |
|  | DNA gyrase subunit A (EC 5.99.1.3) |  |  |  |  |
|  | DNA-directed RNA polymerase beta subunit (EC 2.7.7.6) |  |  |  |  |
|  | Translation elongation factor Tu |  |  |  |  |
|  | Transcription termination factor Rho |  |  |  |  |
|  | 16S rRNA (guanine(527)-N(7))-methyltransferase (EC 2.1.1.170) |  |  |  |  |
|  | Isoleucyl-tRNA synthetase (EC 6.1.1.5) |  |  |  |  |
|  | Translation elongation factor Tu |  |  |  |  |
|  | 3-oxoacyl-[acyl-carrier-protein] synthase, KASII (EC 2.3.1.179) |  |  |  |  |
|  | Multidrug efflux system MdtABC-TolC, inner-membrane proton/drug antiporter MdtB (RND type) |  |  |  |  |
|  | SSU ribosomal protein S10p (S20e) |  |  |  |  |
|  | SSU ribosomal protein S12p (S23e) |  |  |  |  |
|  | Outer membrane factor (OMF) lipoprotein associated wth MdtABC efflux system |  |  |  |  |
|  | Multidrug efflux system, outer membrane factor lipoprotein of OprM/OprM family |  |  |  |  |
|  | Multidrug efflux system MdtABC-TolC, membrane fusion component MdtA |  |  |  |  |
|  | UDP-N-acetylglucosamine 1-carboxyvinyltransferase (EC 2.5.1.7) |  |  |  |  |
|  | D-alanine--D-alanine ligase (EC 6.3.2.4) |  |  |  |  |
|  | Dihydrofolate reductase (EC 1.5.1.3) |  |  |  |  |
|  | Aminoglycoside N(6')-acetyltransferase (EC 2.3.1.82) => AAC(6')-Ic,f,g,h,j,k,l,r-z |  |  |  |  |
|  | SSU ribosomal protein S10p (S20e) |  |  | YP_208874.1 | *Neisseria gonorrhoeae* FA 1090 |
|  | CDP-diacylglycerol--glycerol-3-phosphate 3-phosphatidyltransferase (EC 2.7.8.5) |  |  |  |  |
|  | DNA gyrase subunit B (EC 5.99.1.3) |  |  |  |  |
|  | Dihydropteroate synthase (EC 2.5.1.15) |  |  |  |  |
|  | Hydrogen peroxide-inducible genes activator => OxyR |  |  |  |  |
| *Bordetella petrii* J51 | SSU ribosomal protein S10p (S20e) |  |  | YP_208874.1 | *Neisseria gonorrhoeae* FA 1090 |
|  | Multidrug efflux system, membrane fusion component => MexA of MexAB-OprM |  |  |  |  |
|  | 16S rRNA (guanine(527)-N(7))-methyltransferase (EC 2.1.1.170) |  |  |  |  |
|  | Translation elongation factor Tu |  |  |  |  |
|  | Macrolide-specific efflux protein MacA |  |  |  |  |
|  | Aminoglycoside 3''-nucleotidyltransferase (EC 2.7.7.-) => ANT(3'')-Ia (AadA family) |  |  |  |  |
|  | Glycerophosphoryl diester phosphodiesterase (EC 3.1.4.46) |  |  |  |  |
|  | Translation elongation factor G |  |  |  |  |
|  | Alanine racemase (EC 5.1.1.1) |  |  |  |  |
|  | Translation elongation factor Tu |  |  |  |  |
|  | Multidrug efflux system, outer membrane factor lipoprotein of OprM/OprM family |  |  |  |  |
|  | Multidrug efflux system EmrAB-OMF, membrane fusion component EmrA |  |  |  |  |
|  | Outer membrane factor (OMF) lipoprotein associated wth MdtABC efflux system |  |  |  |  |
|  | 3-oxoacyl-[acyl-carrier-protein] synthase, KASII (EC 2.3.1.179) |  |  |  |  |
|  | DNA gyrase subunit A (EC 5.99.1.3) |  |  |  |  |
|  | Transcription termination factor Rho |  |  |  |  |
|  | 3-oxoacyl-[acyl-carrier-protein] synthase, KASII (EC 2.3.1.179) |  |  |  |  |
|  | D-alanine--D-alanine ligase (EC 6.3.2.4) |  |  |  |  |
|  | Translation elongation factor G |  |  |  |  |
|  | DNA-directed RNA polymerase beta' subunit (EC 2.7.7.6) |  |  |  |  |
|  | Hydrogen peroxide-inducible genes activator => OxyR |  |  |  |  |
|  | CDP-diacylglycerol--glycerol-3-phosphate 3-phosphatidyltransferase (EC 2.7.8.5) |  |  |  |  |
|  | UDP-N-acetylglucosamine 1-carboxyvinyltransferase (EC 2.5.1.7) |  |  |  |  |
|  | Enoyl-[acyl-carrier-protein] reductase [NADH] (EC 1.3.1.9) |  |  |  |  |
|  | Multidrug efflux system MdtABC-TolC, membrane fusion component MdtA |  |  |  |  |
|  | Dihydrofolate reductase (EC 1.5.1.3) |  |  |  |  |
|  | Multidrug efflux system MdtABC-TolC, inner-membrane proton/drug antiporter MdtC (RND type) |  |  |  |  |
|  | SSU ribosomal protein S12p (S23e) |  |  |  |  |
|  | Aminoglycoside 6-phosphotransferase, putative |  |  |  |  |
|  | Undecaprenyl-diphosphatase BcrC (EC 3.6.1.27), conveys bacitracin resistance |  |  |  |  |
|  | 1-deoxy-D-xylulose 5-phosphate reductoisomerase (EC 1.1.1.267) |  |  |  |  |
|  | DNA-directed RNA polymerase beta subunit (EC 2.7.7.6) |  |  |  |  |
|  | Isoleucyl-tRNA synthetase (EC 6.1.1.5) |  |  |  |  |
|  | Outer membrane factor (OMF) lipoprotein associated wth EmrAB-OMF efflux system |  |  |  |  |
|  | LSU ribosomal protein L6p (L9e) |  |  |  |  |
|  | Multidrug efflux system MdtABC-TolC, inner-membrane proton/drug antiporter MdtB (RND type) |  |  |  |  |
|  | Dihydropteroate synthase (EC 2.5.1.15) |  |  |  |  |
|  | Macrolide export ATP-binding/permease protein MacB |  |  |  |  |
|  | DNA gyrase subunit B (EC 5.99.1.3) |  |  |  |  |
|  | Class C beta-lactamase (EC 3.5.2.6) |  |  |  |  |
|  | Glycerophosphoryl diester phosphodiesterase (EC 3.1.4.46) |  |  |  |  |
|  | Multidrug efflux system EmrAB-OMF, inner-membrane proton/drug antiporter EmrB (MFS type) |  |  |  |  |
|  | SSU ribosomal protein S10p (S20e) |  |  |  |  |
| *Bordetella petrii* BMC_SI_3 | Multidrug efflux system MdtABC-TolC, inner-membrane proton/drug antiporter MdtC (RND type) | Multidrug efflux system MdtABC-TolC, inner-membrane proton/drug antiporter MdtC (RND type) | MdtABC-TolC |  |  |
|  | RND efflux system, inner membrane transporter |  | ceoB | AAB58161.1 | *Burkholderia cepacia* |
|  | Outer membrane factor (OMF) lipoprotein associated wth EmrAB-OMF efflux system | Outer membrane factor (OMF) lipoprotein associated wth EmrAB-OMF efflux system | EmrAB-OMF |  |  |
|  | 1-deoxy-D-xylulose 5-phosphate reductoisomerase (EC 1.1.1.267) | 1-deoxy-D-xylulose 5-phosphate reductoisomerase (EC 1.1.1.267) | dxr |  |  |
|  | Class C beta-lactamase (EC 3.5.2.6) | Class C beta-lactamase (EC 3.5.2.6) |  |  |  |
|  | Dihydropteroate synthase (EC 2.5.1.15) | Dihydropteroate synthase (EC 2.5.1.15) | folP |  |  |
|  | Translation elongation factor Tu | Translation elongation factor Tu | EF-Tu |  |  |
|  | Multidrug efflux system MdtABC-TolC, membrane fusion component MdtA | Multidrug efflux system MdtABC-TolC, membrane fusion component MdtA | MdtABC-TolC |  |  |
|  | Transcription termination factor Rho | Transcription termination factor Rho | rho |  |  |
|  | CDP-diacylglycerol--glycerol-3-phosphate 3-phosphatidyltransferase (EC 2.7.8.5) | CDP-diacylglycerol--glycerol-3-phosphate 3-phosphatidyltransferase (EC 2.7.8.5) | PgsA |  |  |
|  | Alanine racemase (EC 5.1.1.1) | Alanine racemase (EC 5.1.1.1) | Alr |  |  |
|  | Dihydrofolate reductase (EC 1.5.1.3) | Dihydrofolate reductase (EC 1.5.1.3) | folA, Dfr |  |  |
|  | 3-oxoacyl-[acyl-carrier-protein] synthase, KASII (EC 2.3.1.179) | 3-oxoacyl-[acyl-carrier-protein] synthase, KASII (EC 2.3.1.179) | kasA |  |  |
|  | Translation elongation factor Tu | Translation elongation factor Tu | EF-Tu |  |  |
|  | Translation elongation factor Tu | Translation elongation factor Tu | EF-Tu |  |  |
|  | SSU ribosomal protein S10p (S20e) | SSU ribosomal protein S10p (S20e) | S10p |  |  |
|  | DNA gyrase subunit A (EC 5.99.1.3) | DNA gyrase subunit A (EC 5.99.1.3) | gyrA |  |  |
|  | Isoleucyl-tRNA synthetase (EC 6.1.1.5) | Isoleucyl-tRNA synthetase (EC 6.1.1.5) | Iso-tRNA |  |  |
|  | SSU ribosomal protein S10p (S20e) |  | rpsJ | YP_208874.1 | *Neisseria gonorrhoeae* FA 1090 |
|  | Multidrug efflux system EmrAB-OMF, membrane fusion component EmrA | Multidrug efflux system EmrAB-OMF, membrane fusion component EmrA | EmrAB-TolC |  |  |
|  | Multidrug efflux system, membrane fusion component => MexA of MexAB-OprM | Multidrug efflux system, membrane fusion component => MexA of MexAB-OprM | MexAB-OprM |  |  |
|  | 16S rRNA (guanine(527)-N(7))-methyltransferase (EC 2.1.1.170) | 16S rRNA (guanine(527)-N(7))-methyltransferase (EC 2.1.1.170) | gidB |  |  |
|  | SSU ribosomal protein S12p (S23e) | SSU ribosomal protein S12p (S23e) | S12p |  |  |
|  | Aminoglycoside 3'-phosphotransferase (EC 2.7.1.95) => APH(3')-II/APH(3')-XV | Aminoglycoside 3'-phosphotransferase (EC 2.7.1.95) => APH(3')-II/APH(3')-XV | APH(3')-II/APH(3')-XV |  |  |
|  | Hydrogen peroxide-inducible genes activator => OxyR | Hydrogen peroxide-inducible genes activator => OxyR | OxyR |  |  |
|  | DNA-directed RNA polymerase beta subunit (EC 2.7.7.6) | DNA-directed RNA polymerase beta subunit (EC 2.7.7.6) | rpoB |  |  |
|  | DNA-directed RNA polymerase beta' subunit (EC 2.7.7.6) | DNA-directed RNA polymerase beta' subunit (EC 2.7.7.6) | rpoC |  |  |
|  | Multidrug efflux system, inner membrane proton/drug antiporter (RND type) => MexB of MexAB-OprM | Multidrug efflux system, inner membrane proton/drug antiporter (RND type) => MexB of MexAB-OprM | MexAB-OprM |  |  |
|  | Multidrug efflux system EmrAB-OMF, inner-membrane proton/drug antiporter EmrB (MFS type) | Multidrug efflux system EmrAB-OMF, inner-membrane proton/drug antiporter EmrB (MFS type) | EmrAB-TolC |  |  |
|  | UDP-N-acetylglucosamine 1-carboxyvinyltransferase (EC 2.5.1.7) | UDP-N-acetylglucosamine 1-carboxyvinyltransferase (EC 2.5.1.7) | MurA |  |  |
|  | Glycerophosphoryl diester phosphodiesterase (EC 3.1.4.46) | Glycerophosphoryl diester phosphodiesterase (EC 3.1.4.46) | GdpD |  |  |
|  | Translation elongation factor G | Translation elongation factor G | EF-G |  |  |
|  | Multidrug efflux system MdtABC-TolC, inner-membrane proton/drug antiporter MdtB (RND type) | Multidrug efflux system MdtABC-TolC, inner-membrane proton/drug antiporter MdtB (RND type) | MdtABC-TolC |  |  |
|  | Enoyl-[acyl-carrier-protein] reductase [NADH] (EC 1.3.1.9) | Enoyl-[acyl-carrier-protein] reductase [NADH] (EC 1.3.1.9) | inhA, fabI |  |  |
|  | DNA gyrase subunit B (EC 5.99.1.3) | DNA gyrase subunit B (EC 5.99.1.3) | gyrB |  |  |
|  | Outer membrane factor (OMF) lipoprotein associated wth MdtABC efflux system | Outer membrane factor (OMF) lipoprotein associated wth MdtABC efflux system | MdtABC-OMF |  |  |
|  | Translation elongation factor G | Translation elongation factor G | EF-G |  |  |
|  | D-alanine--D-alanine ligase (EC 6.3.2.4) | D-alanine--D-alanine ligase (EC 6.3.2.4) | Ddl |  |  |
|  | Translation elongation factor Tu | Translation elongation factor Tu | EF-Tu |  |  |
|  | Multidrug efflux system, outer membrane factor lipoprotein of OprM/OprM family | Multidrug efflux system, outer membrane factor lipoprotein of OprM/OprM family | OprM/OprM family |  |  |
| *Bordetella petrii* strain BT 1 9.2 | DNA-directed RNA polymerase beta' subunit (EC 2.7.7.6) | DNA-directed RNA polymerase beta' subunit (EC 2.7.7.6) | rpoC |  |  |
|  | Multidrug efflux system EmrAB-OMF, membrane fusion component EmrA | Multidrug efflux system EmrAB-OMF, membrane fusion component EmrA | EmrAB-TolC |  |  |
|  | Hydrogen peroxide-inducible genes activator => OxyR | Hydrogen peroxide-inducible genes activator => OxyR | OxyR |  |  |
|  | Multidrug efflux system EmrAB-OMF, membrane fusion component EmrA | Multidrug efflux system EmrAB-OMF, membrane fusion component EmrA | EmrAB-TolC |  |  |
|  | Translation elongation factor G | Translation elongation factor G | EF-G |  |  |
|  | DNA gyrase subunit B (EC 5.99.1.3) | DNA gyrase subunit B (EC 5.99.1.3) | gyrB |  |  |
|  | Transcription termination factor Rho | Transcription termination factor Rho | rho |  |  |
|  | Multidrug efflux system EmrAB-OMF, inner-membrane proton/drug antiporter EmrB (MFS type) | Multidrug efflux system EmrAB-OMF, inner-membrane proton/drug antiporter EmrB (MFS type) | EmrAB-TolC |  |  |
|  | DNA gyrase subunit A (EC 5.99.1.3) | DNA gyrase subunit A (EC 5.99.1.3) | gyrA |  |  |
|  | Aminoglycoside 6-phosphotransferase, putative | Aminoglycoside 6-phosphotransferase, putative |  |  |  |
|  | UDP-N-acetylglucosamine 1-carboxyvinyltransferase (EC 2.5.1.7) | UDP-N-acetylglucosamine 1-carboxyvinyltransferase (EC 2.5.1.7) | MurA |  |  |
|  | Multidrug efflux system, outer membrane factor lipoprotein of OprM/OprM family | Multidrug efflux system, outer membrane factor lipoprotein of OprM/OprM family | OprM/OprM family |  |  |
|  | Enoyl-[acyl-carrier-protein] reductase [NADH] (EC 1.3.1.9) | Enoyl-[acyl-carrier-protein] reductase [NADH] (EC 1.3.1.9) | inhA, fabI |  |  |
|  | Outer membrane factor (OMF) lipoprotein associated wth MdtABC efflux system | Outer membrane factor (OMF) lipoprotein associated wth MdtABC efflux system | MdtABC-OMF |  |  |
|  | 1-deoxy-D-xylulose 5-phosphate reductoisomerase (EC 1.1.1.267) | 1-deoxy-D-xylulose 5-phosphate reductoisomerase (EC 1.1.1.267) | dxr |  |  |
|  | Multidrug efflux system MdtABC-TolC, inner-membrane proton/drug antiporter MdtB (RND type) | Multidrug efflux system MdtABC-TolC, inner-membrane proton/drug antiporter MdtB (RND type) | MdtABC-TolC |  |  |
|  | DNA-directed RNA polymerase beta subunit (EC 2.7.7.6) | DNA-directed RNA polymerase beta subunit (EC 2.7.7.6) | rpoB |  |  |
|  | 16S rRNA (guanine(527)-N(7))-methyltransferase (EC 2.1.1.170) | 16S rRNA (guanine(527)-N(7))-methyltransferase (EC 2.1.1.170) | gidB |  |  |
|  | 3-oxoacyl-[acyl-carrier-protein] synthase, KASII (EC 2.3.1.179) | 3-oxoacyl-[acyl-carrier-protein] synthase, KASII (EC 2.3.1.179) | kasA |  |  |
|  | Translation elongation factor Tu | Translation elongation factor Tu | EF-Tu |  |  |
|  | Glycerophosphoryl diester phosphodiesterase (EC 3.1.4.46) | Glycerophosphoryl diester phosphodiesterase (EC 3.1.4.46) | GdpD |  |  |
|  | CDP-diacylglycerol--glycerol-3-phosphate 3-phosphatidyltransferase (EC 2.7.8.5) | CDP-diacylglycerol--glycerol-3-phosphate 3-phosphatidyltransferase (EC 2.7.8.5) | PgsA |  |  |
|  | D-alanine--D-alanine ligase (EC 6.3.2.4) | D-alanine--D-alanine ligase (EC 6.3.2.4) | Ddl |  |  |
|  | SSU ribosomal protein S10p (S20e) | SSU ribosomal protein S10p (S20e) | S10p |  |  |
|  | Translation elongation factor Tu | Translation elongation factor Tu | EF-Tu |  |  |
|  | RND efflux system, inner membrane transporter |  | ceoB | AAB58161.1 | *Burkholderia cepacia* |
|  | Multidrug efflux system MdtABC-TolC, inner-membrane proton/drug antiporter MdtC (RND type) | Multidrug efflux system MdtABC-TolC, inner-membrane proton/drug antiporter MdtC (RND type) | MdtABC-TolC |  |  |
|  | SSU ribosomal protein S10p (S20e) |  | rpsJ | YP_208874.1 | *Neisseria gonorrhoeae* FA 1090 |
|  | Outer membrane factor (OMF) lipoprotein associated wth EmrAB-OMF efflux system | Outer membrane factor (OMF) lipoprotein associated wth EmrAB-OMF efflux system | EmrAB-OMF |  |  |
|  | Alanine racemase (EC 5.1.1.1) | Alanine racemase (EC 5.1.1.1) | Alr |  |  |
|  | Multidrug efflux system, membrane fusion component => MexA of MexAB-OprM | Multidrug efflux system, membrane fusion component => MexA of MexAB-OprM | MexAB-OprM |  |  |
|  | 3-oxoacyl-[acyl-carrier-protein] synthase, KASII (EC 2.3.1.179) | 3-oxoacyl-[acyl-carrier-protein] synthase, KASII (EC 2.3.1.179) | kasA |  |  |
|  | Glycerophosphoryl diester phosphodiesterase (EC 3.1.4.46) | Glycerophosphoryl diester phosphodiesterase (EC 3.1.4.46) | GdpD |  |  |
|  | Dihydropteroate synthase (EC 2.5.1.15) | Dihydropteroate synthase (EC 2.5.1.15) | folP |  |  |
|  | Multidrug efflux system MdtABC-TolC, membrane fusion component MdtA | Multidrug efflux system MdtABC-TolC, membrane fusion component MdtA | MdtABC-TolC |  |  |
|  | Multidrug efflux system EmrAB-OMF, inner-membrane proton/drug antiporter EmrB (MFS type) | Multidrug efflux system EmrAB-OMF, inner-membrane proton/drug antiporter EmrB (MFS type) | EmrAB-TolC |  |  |
|  | Translation elongation factor G | Translation elongation factor G | EF-G |  |  |
|  | SSU ribosomal protein S12p (S23e) | SSU ribosomal protein S12p (S23e) | S12p |  |  |
|  | Dihydrofolate reductase (EC 1.5.1.3) | Dihydrofolate reductase (EC 1.5.1.3) | folA, Dfr |  |  |
|  | Isoleucyl-tRNA synthetase (EC 6.1.1.5) | Isoleucyl-tRNA synthetase (EC 6.1.1.5) | Iso-tRNA |  |  |
| **Virulence Factor** | | | | | |
| **Genome Name** | **Product** | **Function** | **Gene** | **Source ID** | **Source Organism** |
| *Bordetella petrii* DSM 12804 | Putative oxidoreductase |  | wlbA | VFG000038 | *Bordetella pertussis Tohama* I |
|  | Flagellar biosynthesis protein FlhA |  | flhA1 | VFG002522 | *Burkholderia pseudomallei* K96243 |
|  | Argininosuccinate synthase (EC 6.3.4.5) |  | argG | 126207906 | *Actinobacillus pleuropneumoniae* L20 |
|  | LSU ribosomal protein L36p @ LSU ribosomal protein L36p, zinc-dependent |  | rpmJ | 56707499 | *Francisella tularensis subsp. tularensis* SCHU S4 |
|  | Putative oxidoreductase |  | wlbA | 187476613 | *Bordetella avium* |
|  | Putative oxidoreductase |  |  | 187476613 | *Bordetella avium* |
|  | 3-phosphoshikimate 1-carboxyvinyltransferase (EC 2.5.1.19) |  | aroA | 33602442 | *Bordetella bronchiseptica* RB50 |
|  | Putative oxidoreductase |  |  | VFG000038 | *Bordetella pertussis Tohama* I |
|  | RNA-binding protein Hfq |  | hfq | S4595 | *Shigella flexneri 2a str.* 2457T |
|  | UDP-2-acetamido-3-amino-2,3-dideoxy-D-glucuronic acid acetyltransferase (EC 2.3.1.201) |  | wlbB | VFG000037 | *Bordetella pertussis Tohama* I |
|  | RecA protein |  | recA | 53718415 | *Burkholderia pseudomallei* K96243 |
|  | Chemotaxis regulator - transmits chemoreceptor signals to flagellar motor components CheY |  | cheY2 | VFG043206 | *Yersinia enterocolitica subsp. enterocolitica* 8081 |
|  | Imidazole glycerol phosphate synthase cyclase subunit |  | hisF | 237814060 | *Burkholderia pseudomallei* MSHR346 |
|  | Glutamate--UDP-2-acetamido-2-deoxy-D-ribohex-3-uluronic acid aminotransferase (PLP cofactor) (EC 2.6.1.98) |  | wlbC | VFG000036 | *Bordetella pertussis Tohama* I |
|  | hypothetical protein |  | wlbL2 | VFG000029 | *Bordetella pertussis Tohama* I |
|  | RNA-binding protein Hfq |  | hfq | 15676646 | *Neisseria meningitidis* MC58 |
|  | Chorismate synthase (EC 4.2.3.5) |  | aroC | 126441149 | *Burkholderia pseudomallei* 668 |
| *Bordetella petrii* J49 | RNA-binding protein Hfq |  |  | S4595 | *Shigella flexneri 2a str.* 2457T |
|  | Positive regulator of CheA protein activity (CheW) |  |  | VFG002530 | *Burkholderia pseudomallei* K96243 |
|  | Translation elongation factor Tu |  |  | 15676067 | *Neisseria meningitidis* MC58 |
|  | LSU ribosomal protein L36p @ LSU ribosomal protein L36p, zinc-dependent |  |  | 56707499 | *Francisella tularensis subsp. tularensis* SCHU S4 |
|  | Putative oxidoreductase |  |  | 187476613 | *Bordetella avium* |
|  | RecA protein |  |  | 53718415 | *Burkholderia pseudomallei* K96243 |
|  | Argininosuccinate synthase (EC 6.3.4.5) |  |  | 126207906 | *Actinobacillus pleuropneumoniae* L20 |
|  | UDP-2-acetamido-3-amino-2,3-dideoxy-D-glucuronic acid acetyltransferase (EC 2.3.1.201) |  |  | VFG000037 | *Bordetella pertussis Tohama* I |
|  | Chemotaxis regulator - transmits chemoreceptor signals to flagellar motor components CheY |  |  | VFG002525 | *Burkholderia pseudomallei* K96243 |
|  | Imidazole glycerol phosphate synthase cyclase subunit |  |  | 237814060 | *Burkholderia pseudomallei* MSHR346 |
|  | 3-phosphoshikimate 1-carboxyvinyltransferase (EC 2.5.1.19) |  |  | 33602442 | *Bordetella bronchiseptica* RB50 |
|  | Translation elongation factor Tu |  |  | 15676067 | *Neisseria meningitidis* MC58 |
|  | Putative oxidoreductase |  |  | VFG000038 | *Bordetella pertussis Tohama* I |
|  | RNA-binding protein Hfq |  |  | 15676646 | *Neisseria meningitidis* MC58 |
|  | Glutamate--UDP-2-acetamido-2-deoxy-D-ribohex-3-uluronic acid aminotransferase (PLP cofactor) (EC 2.6.1.98) |  |  | VFG000036 | *Bordetella pertussis Tohama* I |
| *Bordetella petrii* J51 | 3',5'-cyclic-nucleotide phosphodiesterase (EC 3.1.4.17) |  |  | 33591381 | *Bordetella pertussis Tohama* I |
|  | Imidazole glycerol phosphate synthase cyclase subunit |  |  | 237814060 | *Burkholderia pseudomallei* MSHR346 |
|  | Argininosuccinate synthase (EC 6.3.4.5) |  |  | 126207906 | *Actinobacillus pleuropneumoniae* L20 |
|  | 3-phosphoshikimate 1-carboxyvinyltransferase (EC 2.5.1.19) |  |  | 33602442 | *Bordetella bronchiseptica* RB50 |
|  | RNA-binding protein Hfq |  |  | 15676646 | *Neisseria meningitidis* MC58 |
|  | Chorismate synthase (EC 4.2.3.5) |  |  | 126441149 | *Burkholderia pseudomallei* 668 |
|  | Chemotaxis regulator - transmits chemoreceptor signals to flagellar motor components CheY |  |  | VFG002525 | *Burkholderia pseudomallei* K96243 |
|  | RecA protein |  |  | 53718415 | *Burkholderia pseudomallei* K96243 |
|  | RNA-binding protein Hfq |  |  | S4595 | *Shigella flexneri 2a str.* 2457T |
| *Bordetella petrii* BMC_SI_3 | UDP-2-acetamido-3-amino-2,3-dideoxy-D-glucuronic acid acetyltransferase (EC 2.3.1.201) |  | bplB | VFG000037 | *Bordetella pertussis* Tohama I |
|  | 3',5'-cyclic-nucleotide phosphodiesterase (EC 3.1.4.17) |  | BP0123 | 33591381 | *Bordetella pertussis* Tohama I |
|  | RNA-binding protein Hfq |  | hfq | 15676646 | *Neisseria meningitidis* MC58 |
|  | LSU ribosomal protein L36p @ LSU ribosomal protein L36p, zinc-dependent |  | rpmJ | 56707499 | *Francisella tularensis* subsp. *tularensis* SCHU S4 |
|  | Putative oxidoreductase |  | bplA | 187476613 | *Bordetella avium* |
|  | RNA-binding protein Hfq |  | hfq | S4595 | *Shigella flexneri* 2a str. 2457T |
|  | Translation elongation factor Tu |  | tufA | 15676067 | *Neisseria meningitidis* MC58 |
|  | Translation elongation factor Tu |  | tufA | 15676067 | *Neisseria meningitidis* MC58 |
|  | RecA protein |  | recA | 53718415 | *Burkholderia pseudomallei* K96243 |
|  | Imidazole glycerol phosphate synthase cyclase subunit |  | hisF | 237814060 | *Burkholderia pseudomallei* MSHR346 |
|  | 3-phosphoshikimate 1-carboxyvinyltransferase (EC 2.5.1.19) |  | aroA | 33602442 | *Bordetella bronchiseptica* RB50 |
|  | Argininosuccinate synthase (EC 6.3.4.5) |  | argG | 126207906 | *Actinobacillus pleuropneumoniae* L20 |
|  | Putative oxidoreductase |  | bplA | VFG000038 | *Bordetella pertussis* Tohama I |
|  | Glutamate--UDP-2-acetamido-2-deoxy-D-ribohex-3-uluronic acid aminotransferase (PLP cofactor) (EC 2.6.1.98) |  | bplC | VFG000036 | *Bordetella pertussis* Tohama I |
| *Bordetella petrii* strain BT 1 9.2 | 3-phosphoshikimate 1-carboxyvinyltransferase (EC 2.5.1.19) |  | aroA | 33602442 | *Bordetella bronchiseptica* RB50 |
|  | Argininosuccinate synthase (EC 6.3.4.5) |  | argG | 126207906 | *Actinobacillus pleuropneumoniae* L20 |
|  | Chemotaxis regulator - transmits chemoreceptor signals to flagellar motor components CheY |  | cheY | VFG002525 | *Burkholderia pseudomallei* K96243 |
|  | RecA protein |  | recA | 53718415 | *Burkholderia pseudomallei* K96243 |
|  | RNA-binding protein Hfq |  | hfq | 15676646 | *Neisseria meningitidis* MC58 |
|  | Imidazole glycerol phosphate synthase cyclase subunit |  | hisF | 237814060 | *Burkholderia pseudomallei* MSHR346 |
|  | RNA-binding protein Hfq |  | hfq | S4595 | *Shigella flexneri* 2a str. 2457T |
|  | LSU ribosomal protein L36p @ LSU ribosomal protein L36p, zinc-dependent |  | rpmJ | 56707499 | *Francisella tularensis* subsp. *tularensis* SCHU S4 |
| **Human Homolog** | | | | | |
| **Genome Name** | **Product** | **Function** | **Gene** | **Source ID** | **Source Organism** |
| *Bordetella petrii* DSM 12804 | Maleylacetoacetate isomerase (EC 5.2.1.2) @ Glutathione S-transferase, zeta (EC 2.5.1.18) |  |  | NP_665877.1 | Homo sapiens |
|  | [4Fe-4S] cluster assembly scaffold protein Mrp (=ApbC) |  | mrp2 | NP_079428.2 | Homo sapiens |
|  | Methylcrotonyl-CoA carboxylase carboxyl transferase subunit (EC 6.4.1.4) |  | accB1 | NP_071415.1 | Homo sapiens |
|  | Succinyl-CoA ligase [ADP-forming] alpha chain (EC 6.2.1.5) |  | sucD | NP_003840.2 | Homo sapiens |
|  | hypothetical protein |  |  | NP_006102.2 | Homo sapiens |
|  | NADH-ubiquinone oxidoreductase chain I (EC 1.6.5.3) |  | nuoI | XP_005274072.1 | Homo sapiens |
|  | Succinyl-CoA:3-ketoacid-coenzyme A transferase subunit A (EC 2.8.3.5) |  | lpsI | NP_000427.1 | Homo sapiens |
|  | Succinyl-CoA:3-ketoacid-coenzyme A transferase subunit B (EC 2.8.3.5) |  | scoB | NP_000427.1 | Homo sapiens |
|  | Glycine dehydrogenase [decarboxylating] (glycine cleavage system P protein) (EC 1.4.4.2) |  | gcvP | NP_000161.2 | Homo sapiens |
|  | 2-keto-3-deoxy-L-fuconate dehydrogenase |  |  | NP_064524.3 | Homo sapiens |
|  | 3'-to-5' oligoribonuclease (orn) |  | orn | NP_056338.2 | Homo sapiens |
|  | [4Fe-4S] cluster assembly scaffold protein Mrp (=ApbC) |  | mrp1 | NP_079428.2 | Homo sapiens |
|  | NADH-ubiquinone oxidoreductase chain D (EC 1.6.5.3) |  | nuoD | XP_005245265.1 | Homo sapiens |
|  | Efflux ABC transporter, permease/ATP-binding protein mlr7818 |  |  | NP_001258627.1 | Homo sapiens |
|  | Adenylate kinase (EC 2.7.4.3) |  | adk | NP_037543.1 | Homo sapiens |
|  | Succinyl-CoA:3-ketoacid-coenzyme A transferase subunit B (EC 2.8.3.5) |  |  | NP_000427.1 | Homo sapiens |
|  | S-(hydroxymethyl)glutathione dehydrogenase (EC 1.1.1.284) |  |  | NP_000662.3 | Homo sapiens |
|  | Acetyl-CoA acetyltransferase (EC 2.3.1.9) |  | fadA9 | NP_005882.2 | Homo sapiens |
|  | Glutaredoxin-related protein |  |  | NP_057501.2 | Homo sapiens |
|  | Cysteine desulfurase (EC 2.8.1.7) => IscS |  | iscS | NP_066923.3 | Homo sapiens |
|  | 5-methyltetrahydrofolate--homocysteine methyltransferase (EC 2.1.1.13) |  | metH | NP_000245.2 | Homo sapiens |
|  | ATP-dependent Clp protease ATP-binding subunit ClpX |  | clpX | NP_006651.2 | Homo sapiens |
|  | Hydroxymethylglutaryl-CoA lyase (EC 4.1.3.4) |  |  | NP_000182.2 | Homo sapiens |
|  | Fumarate hydratase class II (EC 4.2.1.2) |  | fumC | NP_000134.2 | Homo sapiens |
|  | Malate dehydrogenase (EC 1.1.1.37) |  | mdh1 | NP_005908.1 | Homo sapiens |
|  | Homogentisate 1,2-dioxygenase (EC 1.13.11.5) |  | hmgA | NP_000178.2 | Homo sapiens |
|  | Catalase KatE (EC 1.11.1.6) |  |  | NP_001743.1 | Homo sapiens |
|  | Peptide-methionine (R)-S-oxide reductase MsrB (EC 1.8.4.12) |  | msrB1 | NP_001180390.1 | Homo sapiens |
|  | Dihydrolipoamide succinyltransferase component (E2) of 2-oxoglutarate dehydrogenase complex (EC 2.3.1.61) |  | odhB | NP_001924.2 | Homo sapiens |
|  | Electron transfer flavoprotein-ubiquinone oxidoreductase (EC 1.5.5.1) |  |  | NP_001268667.1 | Homo sapiens |
|  | Phosphoglycerate mutase (EC 5.4.2.11) |  | gpmA | NP_002620.1 | Homo sapiens |
|  | Pterin-4-alpha-carbinolamine dehydratase (EC 4.2.1.96) |  | phhB | NP_000272.1 | Homo sapiens |
|  | Succinyl-CoA:3-ketoacid-coenzyme A transferase subunit B (EC 2.8.3.5) |  | lpsJ2 | NP_000427.1 | Homo sapiens |
|  | Methylmalonate-semialdehyde dehydrogenase (EC 1.2.1.27) |  | mmsA1 | NP_005580.1 | Homo sapiens |
|  | Cytochrome c oxidase polypeptide I (EC 1.9.3.1) |  | coxA2 | YP_003024028.1 | Homo sapiens |
|  | Electron transfer flavoprotein, beta subunit |  | etfB1 | NP_001976.1 | Homo sapiens |
|  | UDP-glucuronate decarboxylase (EC 4.1.1.35) |  |  | NP_079352.2 | Homo sapiens |
|  | Peptide-methionine (R)-S-oxide reductase MsrB (EC 1.8.4.12) |  | msrB2 | NP_001180390.1 | Homo sapiens |
|  | Phosphoribosylformylglycinamidine cyclo-ligase (EC 6.3.3.1) |  | purM | XP_006724053.1 | Homo sapiens |
|  | Iron-sulfur cluster assembly scaffold protein IscU |  | nifU | NP_998760.1 | Homo sapiens |
|  | Electron transfer flavoprotein, beta subunit |  | etfB2 | NP_001976.1 | Homo sapiens |
|  | Branched-chain acyl-CoA dehydrogenase (EC 1.3.99.12) |  | acd13 | NP_055199.1 | Homo sapiens |
|  | SSU ribosomal protein S12p (S23e) |  | rpsL | NP_203526.1 | Homo sapiens |
|  | Succinate dehydrogenase flavoprotein subunit (EC 1.3.5.1) |  | sdhA | NP_004159.2 | Homo sapiens |
|  | Cyclohex-1-ene-1-carboxyl-CoA hydratase |  |  | NP_004083.3 | Homo sapiens |
|  | Chaperone protein DnaK |  | dnaK | NP_004125.3 | Homo sapiens |
|  | Glutaryl-CoA dehydrogenase (EC 1.3.8.6) |  | acd14 | NP_000150.1 | Homo sapiens |
|  | NAD(P) transhydrogenase subunit beta (EC 1.6.1.2) |  | pntB2 | XP_005248332.1 | Homo sapiens |
|  | Isovaleryl-CoA dehydrogenase (EC 1.3.8.4) |  | ivd | NP_002216.2 | Homo sapiens |
|  | Methylcrotonyl-CoA carboxylase carboxyl transferase subunit (EC 6.4.1.4) |  | pccB | NP_071415.1 | Homo sapiens |
|  | Succinate-semialdehyde dehydrogenase [NAD(P)+] (EC 1.2.1.16) |  | gabD2 | NP_001071.1 | Homo sapiens |
|  | Alkyl hydroperoxide reductase subunit C-like protein |  | lsfA1 | NP_004896.1 | Homo sapiens |
|  | 3-hydroxyacyl-CoA dehydrogenase |  |  | NP_004484.1 | Homo sapiens |
|  | Heat shock protein 60 kDa family chaperone GroEL |  | groEL | XP_005246575.1 | Homo sapiens |
|  | Transaldolase (EC 2.2.1.2) |  | talB | NP_006746.1 | Homo sapiens |
|  | Succinate dehydrogenase iron-sulfur protein (EC 1.3.5.1) |  | sdhB | NP_002991.2 | Homo sapiens |
|  | NADH-ubiquinone oxidoreductase chain B (EC 1.6.5.3) |  | nuoB | NP_077718.3 | Homo sapiens |
|  | ATP synthase alpha chain (EC 3.6.3.14) |  | atpA | NP_001001937.1 | Homo sapiens |
|  | Aconitate hydratase (EC 4.2.1.3) @ 2-methylisocitrate dehydratase (EC 4.2.1.99) |  | acnA | XP_005251533.1 | Homo sapiens |
|  | 3-oxoadipate CoA-transferase subunit A (EC 2.8.3.6) |  |  | NP_000427.1 | Homo sapiens |
|  | Electron transfer flavoprotein, alpha subunit |  | etfA1 | NP_000117.1 | Homo sapiens |
|  | S-formylglutathione hydrolase (EC 3.1.2.12) |  |  | XP_005266335.1 | Homo sapiens |
|  | biphenyl-2,3-diol 1,2-dioxygenase III-related protein |  |  | NP_001073958.2 | Homo sapiens |
|  | ATP synthase beta chain (EC 3.6.3.14) |  | atpD | NP_001677.2 | Homo sapiens |
|  | Protein QmcA (possibly involved in integral membrane quality control) |  |  | NP_038470.1 | Homo sapiens |
|  | Adenosylhomocysteinase (EC 3.3.1.1) |  | acyH | NP_000678.1 | Homo sapiens |
|  | Electron transfer flavoprotein, alpha subunit |  | etfA2 | NP_000117.1 | Homo sapiens |
|  | S-adenosylmethionine synthetase (EC 2.5.1.6) |  | metK | XP_005269899.1 | Homo sapiens |
|  | ATP-dependent Clp protease proteolytic subunit ClpP (EC 3.4.21.92) |  | clpP | NP_006003.1 | Homo sapiens |
|  | Succinyl-CoA:3-ketoacid-coenzyme A transferase subunit B (EC 2.8.3.5) |  |  | NP_000427.1 | Homo sapiens |
|  | NAD(P) transhydrogenase C-domain of subunit alpha (EC 1.6.1.2) |  | pntAB | XP_005248332.1 | Homo sapiens |
|  | Translation elongation factor Tu |  | tuf2 | NP_003312.3 | Homo sapiens |
|  | Succinyl-CoA:3-ketoacid-coenzyme A transferase subunit B (EC 2.8.3.5) |  | lpsJ1 | NP_071403.1 | Homo sapiens |
|  | Translation elongation factor Tu |  | tuf1 | NP_003312.3 | Homo sapiens |
|  | D-Lactate dehydrogenase, cytochrome c-dependent (EC 1.1.2.4) |  |  | NP_919417.1 | Homo sapiens |
| *Bordetella petrii* J49 | Pterin-4-alpha-carbinolamine dehydratase (EC 4.2.1.96) |  |  | NP_000272.1 | Homo sapiens |
|  | Adenosylhomocysteinase (EC 3.3.1.1) |  |  | NP_000678.1 | Homo sapiens |
|  | S-adenosylmethionine synthetase (EC 2.5.1.6) |  |  | XP_005269899.1 | Homo sapiens |
|  | Electron transfer flavoprotein-ubiquinone oxidoreductase (EC 1.5.5.1) |  |  | NP_001268667.1 | Homo sapiens |
|  | NADH-ubiquinone oxidoreductase chain D (EC 1.6.5.3) |  |  | XP_005245265.1 | Homo sapiens |
|  | Hydroxymethylglutaryl-CoA lyase (EC 4.1.3.4) |  |  | NP_000182.2 | Homo sapiens |
|  | Glutaryl-CoA dehydrogenase (EC 1.3.8.6) |  |  | NP_000150.1 | Homo sapiens |
|  | Succinyl-CoA:3-ketoacid-coenzyme A transferase subunit A (EC 2.8.3.5) |  |  | NP_000427.1 | Homo sapiens |
|  | Aconitate hydratase (EC 4.2.1.3) @ 2-methylisocitrate dehydratase (EC 4.2.1.99) |  |  | XP_005251533.1 | Homo sapiens |
|  | 2-keto-3-deoxy-L-fuconate dehydrogenase |  |  | NP_064524.3 | Homo sapiens |
|  | Succinyl-CoA ligase [ADP-forming] alpha chain (EC 6.2.1.5) |  |  | NP_003840.2 | Homo sapiens |
|  | UDP-glucuronate decarboxylase (EC 4.1.1.35) |  |  | NP_079352.2 | Homo sapiens |
|  | S-(hydroxymethyl)glutathione dehydrogenase (EC 1.1.1.284) |  |  | NP_000662.3 | Homo sapiens |
|  | Guanidinobutyrase (EC 3.5.3.7) |  |  | NP_079034.3 | Homo sapiens |
|  | Cyclohex-1-ene-1-carboxyl-CoA hydratase |  |  | NP_004083.3 | Homo sapiens |
|  | Branched-chain acyl-CoA dehydrogenase (EC 1.3.99.12) |  |  | NP_055199.1 | Homo sapiens |
|  | Cysteine desulfurase (EC 2.8.1.7) => IscS |  |  | NP_066923.3 | Homo sapiens |
|  | 3-hydroxyacyl-CoA dehydrogenase |  |  | NP_004484.1 | Homo sapiens |
|  | Adenylate kinase (EC 2.7.4.3) |  |  | NP_037543.1 | Homo sapiens |
|  | Phosphoribosylformylglycinamidine cyclo-ligase (EC 6.3.3.1) |  |  | XP_006724053.1 | Homo sapiens |
|  | Dihydrolipoamide succinyltransferase component (E2) of 2-oxoglutarate dehydrogenase complex (EC 2.3.1.61) |  |  | NP_001924.2 | Homo sapiens |
|  | ATP synthase alpha chain (EC 3.6.3.14) |  |  | NP_001001937.1 | Homo sapiens |
|  | SSU ribosomal protein S12p (S23e) |  |  | NP_203526.1 | Homo sapiens |
|  | NADH-ubiquinone oxidoreductase chain I (EC 1.6.5.3) |  |  | XP_005274072.1 | Homo sapiens |
|  | Uracil-DNA glycosylase, family 1 (EC 3.2.2.27) |  |  | NP_550433.1 | Homo sapiens |
|  | 5-methyltetrahydrofolate--homocysteine methyltransferase (EC 2.1.1.13) |  |  | NP_000245.2 | Homo sapiens |
|  | Catalase KatE (EC 1.11.1.6) |  |  | NP_001743.1 | Homo sapiens |
|  | NAD(P) transhydrogenase subunit beta (EC 1.6.1.2) |  |  | XP_005248332.1 | Homo sapiens |
|  | Succinate dehydrogenase flavoprotein subunit (EC 1.3.5.1) |  |  | NP_004159.2 | Homo sapiens |
|  | Isovaleryl-CoA dehydrogenase (EC 1.3.8.4) |  |  | NP_002216.2 | Homo sapiens |
|  | Glycine dehydrogenase [decarboxylating] (glycine cleavage system P protein) (EC 1.4.4.2) |  |  | NP_000161.2 | Homo sapiens |
|  | ATP synthase beta chain (EC 3.6.3.14) |  |  | NP_001677.2 | Homo sapiens |
|  | Methylmalonate-semialdehyde dehydrogenase (EC 1.2.1.27) |  |  | NP_005580.1 | Homo sapiens |
|  | Protein QmcA (possibly involved in integral membrane quality control) |  |  | NP_038470.1 | Homo sapiens |
|  | Heat shock protein 60 kDa family chaperone GroEL |  |  | XP_005246575.1 | Homo sapiens |
|  | ATP-dependent Clp protease ATP-binding subunit ClpX |  |  | NP_006651.2 | Homo sapiens |
|  | Glutaredoxin-related protein |  |  | NP_057501.2 | Homo sapiens |
|  | Succinate dehydrogenase iron-sulfur protein (EC 1.3.5.1) |  |  | NP_002991.2 | Homo sapiens |
|  | Aldehyde dehydrogenase B (EC 1.2.1.22) |  |  | NP_001188306.1 | Homo sapiens |
|  | ATP-dependent Clp protease proteolytic subunit ClpP (EC 3.4.21.92) |  |  | NP_006003.1 | Homo sapiens |
|  | Peptide-methionine (R)-S-oxide reductase MsrB (EC 1.8.4.12) |  |  | NP_001180390.1 | Homo sapiens |
|  | Catalase KatE (EC 1.11.1.6) |  |  | NP_001743.1 | Homo sapiens |
|  | Iron-sulfur cluster assembly scaffold protein IscU |  |  | NP_998760.1 | Homo sapiens |
|  | Phosphoenolpyruvate carboxykinase [GTP] (EC 4.1.1.32) |  |  | NP_002582.3 | Homo sapiens |
|  | Malate dehydrogenase (EC 1.1.1.37) |  |  | NP_005908.1 | Homo sapiens |
|  | S-formylglutathione hydrolase (EC 3.1.2.12) |  |  | XP_005266335.1 | Homo sapiens |
|  | Delta-1-pyrroline-5-carboxylate dehydrogenase (EC 1.2.1.88) |  |  | NP_733844.1 | Homo sapiens |
|  | Alkyl hydroperoxide reductase subunit C-like protein |  |  | NP_004896.1 | Homo sapiens |
|  | Succinyl-CoA:3-ketoacid-coenzyme A transferase subunit B (EC 2.8.3.5) |  |  | NP_000427.1 | Homo sapiens |
|  | Succinate-semialdehyde dehydrogenase [NAD(P)+] (EC 1.2.1.16) |  |  | NP_001071.1 | Homo sapiens |
|  | NADH-ubiquinone oxidoreductase chain B (EC 1.6.5.3) |  |  | NP_077718.3 | Homo sapiens |
|  | Chaperone protein DnaK |  |  | NP_004125.3 | Homo sapiens |
|  | Heat shock protein 60 kDa family chaperone GroEL |  |  | XP_005246575.1 | Homo sapiens |
|  | Succinate-semialdehyde dehydrogenase [NAD(P)+] (EC 1.2.1.16) |  |  | NP_001071.1 | Homo sapiens |
|  | D-Lactate dehydrogenase, cytochrome c-dependent (EC 1.1.2.4) |  |  | NP_919417.1 | Homo sapiens |
|  | Fumarate hydratase class II (EC 4.2.1.2) |  |  | NP_000134.2 | Homo sapiens |
|  | Transaldolase (EC 2.2.1.2) |  |  | NP_006746.1 | Homo sapiens |
|  | Methylcrotonyl-CoA carboxylase carboxyl transferase subunit (EC 6.4.1.4) |  |  | NP_071415.1 | Homo sapiens |
|  | Phosphoglycerate mutase (EC 5.4.2.11) |  |  | NP_002620.1 | Homo sapiens |
|  | Succinate-semialdehyde dehydrogenase [NAD(P)+] (EC 1.2.1.16) |  |  | NP_001071.1 | Homo sapiens |
| Bordetella petrii J51 | NADH-ubiquinone oxidoreductase chain D (EC 1.6.5.3) |  |  | XP_005245265.1 | Homo sapiens |
|  | Methylmalonate-semialdehyde dehydrogenase (EC 1.2.1.27) |  |  | NP_005580.1 | Homo sapiens |
|  | Chaperone protein DnaK |  |  | NP_004125.3 | Homo sapiens |
|  | Glutaryl-CoA dehydrogenase (EC 1.3.8.6) |  |  | NP_000150.1 | Homo sapiens |
|  | Maleylacetoacetate isomerase (EC 5.2.1.2) @ Glutathione S-transferase, zeta (EC 2.5.1.18) |  |  | NP_665877.1 | Homo sapiens |
|  | Glutaryl-CoA dehydrogenase (EC 1.3.8.6) |  |  | NP_000150.1 | Homo sapiens |
|  | S-formylglutathione hydrolase (EC 3.1.2.12) |  |  | XP_005266335.1 | Homo sapiens |
|  | ATP-dependent Clp protease ATP-binding subunit ClpX |  |  | NP_006651.2 | Homo sapiens |
|  | Peptide-methionine (R)-S-oxide reductase MsrB (EC 1.8.4.12) |  |  | NP_001180390.1 | Homo sapiens |
|  | NADH-ubiquinone oxidoreductase chain B (EC 1.6.5.3) |  |  | NP_077718.3 | Homo sapiens |
|  | NAD(P) transhydrogenase C-domain of subunit alpha (EC 1.6.1.2) |  |  | XP_005248332.1 | Homo sapiens |
|  | ATP synthase alpha chain (EC 3.6.3.14) |  |  | NP_001001937.1 | Homo sapiens |
|  | NADH-ubiquinone oxidoreductase chain I (EC 1.6.5.3) |  |  | XP_005274072.1 | Homo sapiens |
|  | Uracil-DNA glycosylase, family 1 (EC 3.2.2.27) |  |  | NP_550433.1 | Homo sapiens |
|  | Iron-sulfur cluster assembly scaffold protein IscU |  |  | NP_998760.1 | Homo sapiens |
|  | Succinate dehydrogenase flavoprotein subunit (EC 1.3.5.1) |  |  | NP_004159.2 | Homo sapiens |
|  | [4Fe-4S] cluster assembly scaffold protein Mrp (=ApbC) |  |  | NP_079428.2 | Homo sapiens |
|  | Succinate dehydrogenase iron-sulfur protein (EC 1.3.5.1) |  |  | NP_002991.2 | Homo sapiens |
|  | Heat shock protein 60 kDa family chaperone GroEL |  |  | XP_005246575.1 | Homo sapiens |
|  | Isovaleryl-CoA dehydrogenase (EC 1.3.8.4) |  |  | NP_002216.2 | Homo sapiens |
|  | Succinate-semialdehyde dehydrogenase [NAD(P)+] (EC 1.2.1.16) |  |  | NP_001071.1 | Homo sapiens |
|  | Cysteine desulfurase (EC 2.8.1.7) => IscS |  |  | NP_066923.3 | Homo sapiens |
|  | Methylcrotonyl-CoA carboxylase carboxyl transferase subunit (EC 6.4.1.4) |  |  | NP_071415.1 | Homo sapiens |
|  | Ribulose-5-phosphate 4-epimerase and related epimerases and aldolases |  |  | NP_054909.2 | Homo sapiens |
|  | Electron transfer flavoprotein-ubiquinone oxidoreductase (EC 1.5.5.1) |  |  | NP_001268667.1 | Homo sapiens |
|  | Electron transfer flavoprotein, alpha subunit |  |  | NP_000117.1 | Homo sapiens |
|  | Phosphoglycerate mutase (EC 5.4.2.11) |  |  | NP_002620.1 | Homo sapiens |
|  | Malate dehydrogenase (EC 1.1.1.37) |  |  | NP_005908.1 | Homo sapiens |
|  | Phosphoribosylformylglycinamidine cyclo-ligase (EC 6.3.3.1) |  |  | XP_006724053.1 | Homo sapiens |
|  | Succinyl-CoA ligase [ADP-forming] alpha chain (EC 6.2.1.5) |  |  | NP_003840.2 | Homo sapiens |
|  | UDP-glucuronate decarboxylase (EC 4.1.1.35) |  |  | NP_001240804.1 | Homo sapiens |
|  | Pterin-4-alpha-carbinolamine dehydratase (EC 4.2.1.96) |  |  | NP_000272.1 | Homo sapiens |
|  | Fumarate hydratase class II (EC 4.2.1.2) |  |  | NP_000134.2 | Homo sapiens |
|  | Adenosylhomocysteinase (EC 3.3.1.1) |  |  | NP_000678.1 | Homo sapiens |
|  | 3-ketoacyl-CoA thiolase (EC 2.3.1.16) |  |  | XP_005271160.1 | Homo sapiens |
|  | Adenylate kinase (EC 2.7.4.3) |  |  | NP_037543.1 | Homo sapiens |
|  | 5-methyltetrahydrofolate--homocysteine methyltransferase (EC 2.1.1.13) |  |  | NP_000245.2 | Homo sapiens |
|  | 3-hydroxyacyl-CoA dehydrogenase |  |  | NP_004484.1 | Homo sapiens |
|  | Cyclohex-1-ene-1-carboxyl-CoA hydratase |  |  | NP_004083.3 | Homo sapiens |
|  | Protein QmcA (possibly involved in integral membrane quality control) |  |  | NP_038470.1 | Homo sapiens |
|  | SSU ribosomal protein S12p (S23e) |  |  | NP_203526.1 | Homo sapiens |
|  | Alkyl hydroperoxide reductase subunit C-like protein |  |  | NP_004896.1 | Homo sapiens |
|  | Electron transfer flavoprotein, beta subunit |  |  | NP_001976.1 | Homo sapiens |
|  | 2-keto-3-deoxy-L-fuconate dehydrogenase |  |  | NP_064524.3 | Homo sapiens |
|  | Heat shock protein 60 kDa family chaperone GroEL |  |  | XP_005246575.1 | Homo sapiens |
|  | Catalase KatE (EC 1.11.1.6) |  |  | NP_001743.1 | Homo sapiens |
|  | Dihydrolipoamide succinyltransferase component (E2) of 2-oxoglutarate dehydrogenase complex (EC 2.3.1.61) |  |  | NP_001924.2 | Homo sapiens |
|  | D-Lactate dehydrogenase, cytochrome c-dependent (EC 1.1.2.4) |  |  | NP_919417.1 | Homo sapiens |
|  | ATP-dependent Clp protease proteolytic subunit ClpP (EC 3.4.21.92) |  |  | NP_006003.1 | Homo sapiens |
|  | [4Fe-4S] cluster assembly scaffold protein Mrp (=ApbC) |  |  | NP_079428.2 | Homo sapiens |
|  | Hydroxymethylglutaryl-CoA lyase (EC 4.1.3.4) |  |  | NP_000182.2 | Homo sapiens |
|  | Succinyl-CoA:3-ketoacid-coenzyme A transferase subunit A (EC 2.8.3.5) |  |  | NP_000427.1 | Homo sapiens |
|  | S-adenosylmethionine synthetase (EC 2.5.1.6) |  |  | XP_005269899.1 | Homo sapiens |
|  | Homogentisate 1,2-dioxygenase (EC 1.13.11.5) |  |  | NP_000178.2 | Homo sapiens |
|  | ATP-dependent Clp protease proteolytic subunit ClpP (EC 3.4.21.92) |  |  | NP_006003.1 | Homo sapiens |
|  | Glycine dehydrogenase [decarboxylating] (glycine cleavage system P protein) (EC 1.4.4.2) |  |  | NP_000161.2 | Homo sapiens |
|  | ATP synthase beta chain (EC 3.6.3.14) |  |  | NP_001677.2 | Homo sapiens |
|  | Branched-chain acyl-CoA dehydrogenase (EC 1.3.99.12) |  |  | NP_055199.1 | Homo sapiens |
|  | Transaldolase (EC 2.2.1.2) |  |  | NP_006746.1 | Homo sapiens |
|  | S-(hydroxymethyl)glutathione dehydrogenase (EC 1.1.1.284) |  |  | NP_000662.3 | Homo sapiens |
|  | Succinyl-CoA:3-ketoacid-coenzyme A transferase subunit B (EC 2.8.3.5) |  |  | NP_000427.1 | Homo sapiens |
|  | biphenyl-2,3-diol 1,2-dioxygenase III-related protein |  |  | NP_001073958.2 | Homo sapiens |
