# Supplementary material for: Genomic, morphological, and biochemical analyses of a multi-metal resistant but multi-drug susceptible strain of Bordetella petrii from hospital soil
Source: Sci Rep. 2022 May 19;12:8439. doi: 10.1038/s41598-022-12435-7 (PMC9120033; doi:10.1038/s41598-022-12435-7)
Supplement: Supplementary file 4 — Supplementary Information 4. [file 41598_2022_12435_MOESM4_ESM.docx]

| **Gene/Protein**  **Supplementary File 4.** Unique genes present within different species of the genus *Bordetella*  Unique genes were identified from all sixteen genomes of different species of *Bordetella* that were compared along with the available strains of *B*. *petrii* using *B*. *petrii* DSM 12804 as a reference. | **Position**  **(Kbp)** | **Organism** | | | | | | | | | | | | | | | | |
| --- | --- | --- | --- | --- | --- | --- | --- | --- | --- | --- | --- | --- | --- | --- | --- | --- | --- | --- |
|  |  | DSM_12804 | BT_19.2 | BMC_SI_3 | J51 | J49 | H050680373 | AU17976 | AU10664 | Tohama_I | 253 | Bpp5 | 51541 | H044680328 | 197N | F582 | HI4681 |  |
| hipA | 0-50 | P |  |  |  |  |  |  |  |  |  |  |  |  |  |  |  |  |
| pat_1 | 40-60 | P |  | P |  |  |  |  | P |  |  |  |  |  |  |  |  |  |
| aceK | 60-80 | P | P | P | P | P |  |  | P | P | P |  |  |  |  |  |  |  |
| sasA_1 | 100-120 | P | p |  |  |  |  |  |  |  |  |  |  |  |  |  |  |  |
| pyrK | 100-120 | P | P |  |  |  |  |  |  |  |  |  |  |  |  |  |  |  |
| IS110 family transposase IS1663 | 100-120 | P |  | P |  |  |  |  |  | p |  |  |  |  |  |  |  |  |
| fimD | 120-130 | P |  | P | p |  |  |  |  |  |  |  |  |  |  |  |  |  |
| IS3 family transposase ISpa 195 | 220-230 | P | P |  |  | P | P |  |  |  |  |  | P | p |  |  |  |  |
| IS3 family transposaseISRso12 | 220-240 | P | P |  |  | P |  |  |  | P |  |  | p | p |  |  | p |  |
| IS3 family transposase ISBxe1 | 220-240 | P |  |  |  |  | P |  |  |  |  |  | p |  |  |  |  |  |
| Msh_A3 | 260-280 | P |  | P | P |  | P | P | p |  |  |  |  |  |  |  |  |  |
| bepA_1 | 260-280 | P |  | P | P |  | P | P | P |  |  |  |  |  |  |  |  |  |
| Putative monooxygenase | 280-300 | P |  | P |  | P |  |  |  |  |  |  |  |  |  |  |  |  |
| alsT | 320-340 | P |  | P | P |  |  |  |  |  |  |  |  |  |  |  |  |  |
| nosF_1 | 320-340 | P | P | P | P | P | P | P | P | P | P | P | P |  |  |  | p |  |
| IS110 family transposase IS1663 | 380-400 | P |  | P |  |  |  |  | P | p |  |  |  |  |  |  |  |  |
| argP_1 | 380-400 | P | P | P | P | P |  | P | P |  |  |  | P |  |  |  |  |  |
| Nitronate monooxygenase | 420-440 | P | P |  | P |  |  |  | p |  |  |  |  |  |  |  |  |  |
| Trehalase | 420-440 | P | P | P | P | P | P | P | P |  |  |  |  |  |  |  |  |  |
| frdB | 420-440 | P | P | P | P | P | P | P | P |  |  |  |  |  |  |  |  |  |
| ttdA/ttdB | 420-480 | p |  |  |  |  |  |  |  |  |  |  |  |  |  |  |  |  |
| Yfde_2 | 500-520 | P |  |  |  | P | p |  |  |  |  |  |  |  |  |  |  |  |
| btuB_1 | 520-550 | P | P | P | P | P | P |  |  |  |  |  |  |  |  |  |  |  |
| IS3 family transposase ISBope_1 | 540-560 | p |  |  |  |  |  |  |  |  |  |  |  |  |  |  |  |  |
| shlB | 660-680 | p |  |  |  |  |  |  |  |  |  |  |  |  |  |  |  |  |
| IS3 family transposase ISPa31 | 660-680 | p |  |  |  |  |  |  |  |  |  |  |  |  |  |  |  |  |
| IS3 family transposase ISBope_1 | 720-740 | p |  |  |  |  |  |  |  |  |  |  |  |  |  |  |  |  |
| ybaK_1 | 720-740 | P | P | P | P | P | P |  |  |  |  |  |  |  |  |  |  |  |
| KdgA 1 | 960-970 | p | p | p | p | p | p | p | p |  |  |  |  |  |  | p | p |  |
| KdgK 1 |  | p | p | p | p | p | p | p | p |  |  |  |  |  |  | p | p |  |
| Light activated DNA binding protein EL222 | 970-980 | P | P |  | P |  |  |  |  |  |  |  |  | P |  |  |  |  |
| fadA_1 |  | p | p | p | p |  |  |  |  |  |  |  |  | p |  |  |  |  |
| Solute binding protein | 980-990 | p |  | p |  |  |  |  |  |  |  |  |  | p |  |  |  |  |
| yhjB | 1020-1030 | p | p | p |  | p |  |  |  |  |  |  |  |  |  |  |  |  |
| IS3 family transposase ISKpn40 | 1030-1040 | p | p | p |  | p |  |  | p | p |  |  |  | p |  |  |  |  |
| xerC_1 |  | p |  |  |  |  |  |  |  |  |  |  |  | p |  |  |  |  |
| arsH_1 | 1080-1090 | p | p | p | p | p | p | p | p | p | p | p |  | p | p |  | p |  |
| hmrR_1 |  | p |  |  |  |  |  |  |  |  |  |  |  |  |  |  |  |  |
| IS3 family transposase ISAzo10 |  | p |  |  |  |  |  |  |  |  |  |  |  |  |  |  |  |  |
| IS3 family transposase ISRme 15 | 1090-1100 | p | p |  |  |  | p |  | p |  |  |  |  |  |  |  |  |  |
| cueR |  | p |  |  |  |  |  |  |  |  |  |  |  |  |  |  |  |  |
| arsC_1 |  | p | p | p | p | p | p |  | p | p | p | p | p | p | p | p | p |  |
| acr3_1 | 1090_ 1100 | p | p | p | p |  | p |  | p |  |  |  |  |  |  | p | p |  |
| arsc_2 |  | p | p | p | p |  | p | p | p |  |  |  |  |  |  | p | p |  |
| cadl |  | p |  |  |  |  | p |  |  |  |  |  |  |  |  |  |  |  |
| soj_1 |  | p | p |  |  |  | p |  |  |  |  | p |  |  |  |  | p |  |
| single stranded DNA binding protein | 1100-1110 | p | p |  |  |  | p |  |  |  |  | p |  |  |  | p |  |  |
| topB_2 |  | p | p |  |  |  | p |  |  |  |  | p |  |  |  | p |  |  |
| ydip |  | p |  |  |  |  | p |  |  |  |  |  |  |  |  |  |  |  |
| xerD_1 |  | p |  |  |  |  |  |  |  |  |  |  |  |  |  |  |  |  |
| xerC_2 |  | p |  |  |  |  |  |  |  |  |  |  |  |  |  |  |  |  |
| xerC_3 | 1110-1120 | p |  |  |  |  |  |  |  |  |  |  |  |  |  |  |  |  |
| IS256 family transposase ISBeen18 | 1150-1160 | p | p |  |  | p |  | p |  |  |  |  |  |  |  |  |  |  |
| IS110 family transposase ISAfe1 | 1160-1170 | p | p |  |  |  |  |  |  |  |  |  |  |  |  |  |  |  |
| rdpA_1 |  | p | p |  |  |  |  |  |  |  |  |  |  |  |  |  |  |  |
| IS3 family transposase ISIde1 |  | p |  |  |  |  |  |  |  |  |  |  |  |  |  |  |  |  |
| rdpA_2 | 1160-1170 | p |  |  |  |  |  |  |  |  |  |  |  |  |  |  |  |  |
| IS3 family transposase ISPa31 |  | p |  |  |  |  |  |  |  |  |  |  |  |  |  |  |  |  |
| IS3 family tramsposase ISAs20 |  | p | p |  |  |  |  |  |  |  |  |  |  |  |  |  |  |  |
| IS3 family transposase ISRme15 | 1170-1180 | p | p |  |  |  | p |  | p |  |  |  |  |  |  |  |  |  |
| ISL3 family transposase ISIde 1 |  | p |  |  |  |  |  |  |  |  |  |  |  |  |  |  |  |  |
| ape 3 |  | p |  |  |  |  |  |  |  |  |  |  |  |  |  |  |  |  |
| IS3 family transposase ISAs20 |  | P |  |  |  |  |  |  |  |  |  |  |  |  |  |  |  |  |
| IS3 family transposase |  | P |  |  |  |  |  |  |  |  |  |  |  |  |  |  |  |  |
| mmgC_3 |  | p | P |  |  |  |  |  |  |  |  |  |  |  |  |  |  |  |
| Phenylacetate coenzyme Aligase | 1180-1190 | p | p |  |  |  |  |  |  |  |  |  |  |  |  |  |  |  |
| Crotonyl_coAhydratase |  | p | P |  |  |  |  |  |  |  |  |  |  |  |  |  |  |  |
| uctC_5 |  | p | P |  |  |  |  |  |  |  |  |  |  |  |  |  |  |  |
| fadD_2 |  | p | p |  |  |  |  |  |  |  |  |  |  |  |  |  |  |  |
| Putative oxidoreductase |  | p | p |  |  |  |  |  |  |  |  |  |  |  |  |  |  |  |
| Long chain fatty acid_coA ligase FadD15 |  | p | p |  |  |  |  |  |  |  |  |  |  |  |  |  |  |  |
| IS3 family transposase ISBxe1 |  | p |  |  |  |  | p |  |  |  |  |  |  |  |  |  |  |  |
| livH_3 | 1190-1200 | P | P |  |  |  |  |  |  |  |  |  |  |  |  |  |  |  |
| dpgD_1 |  | P | p |  |  |  |  |  |  |  |  |  |  |  |  |  |  |  |
| fabG_3 |  | P | P |  |  |  |  |  |  |  |  |  |  |  |  |  |  |  |
| IS3 family transposase ISAs20 | 1200-1210 | P | p | P |  |  |  |  |  |  |  |  |  |  |  |  |  |  |
| IS3 family transposase ISPa31 |  | P | P |  |  |  |  |  |  |  |  |  |  |  |  |  |  |  |
| IS66 family transposase |  | P | p |  |  |  |  |  |  |  |  |  |  |  |  |  |  |  |
| Insertion sequence IS5376 putative ATP binding protein |  | P | p |  |  |  |  |  |  |  |  |  |  |  |  |  |  |  |
| IS3 family transposase ISAs20 | 1200-1250 | p |  |  |  |  |  |  |  |  |  |  |  |  |  |  |  |  |
| IS66 family transposase ISEe8 |  | P | P |  |  |  |  |  |  |  |  |  |  |  |  |  |  |  |
| Insertion sequence IS5376 putative ATP binding protein |  | p | P |  |  |  |  |  |  |  |  |  |  |  |  |  |  |  |
| IS66 family transposase ISBaps1 |  | p | p |  |  |  |  |  |  |  |  |  |  |  |  |  |  |  |
| paaF_1 |  | p | P |  |  |  |  |  |  |  |  |  |  |  |  |  |  |  |
| nemA_1 |  | P |  |  |  |  |  |  |  |  |  |  |  |  |  |  |  |  |
| pgrR_1 |  | P |  |  |  |  |  |  |  |  |  |  |  |  |  |  |  |  |
| dhpH |  | P |  |  |  |  |  |  |  |  |  |  |  |  |  |  |  |  |
| Leu/Ile/Val binding protein |  | P |  |  |  |  |  |  |  |  |  |  |  |  |  |  |  |  |
| cocE | 1250-1300 | p |  |  |  |  |  |  |  |  |  |  |  |  |  |  |  |  |
| Irp_5 |  | P |  |  |  |  |  |  |  |  |  |  |  |  |  |  |  |  |
| garR_2 |  | P |  |  |  |  |  |  |  |  |  |  |  |  |  |  |  |  |
| dmIR_6 |  | P |  |  |  |  |  |  |  |  |  |  |  |  |  |  |  |  |
| IS3 family transposase ISBmu 11 |  | P |  |  |  |  |  |  |  |  |  |  |  |  |  |  |  |  |
| IS3 family transposase ISAs20 |  | P |  |  |  |  |  |  |  |  |  |  |  |  |  |  |  |  |
| dmIR_1 |  | P |  |  |  |  |  |  |  |  |  |  |  |  |  |  |  |  |
| oprM_2 |  | P |  |  |  |  |  |  |  |  |  |  |  |  |  |  |  |  |
| aaeA_1 |  | p |  |  |  |  |  |  |  |  |  |  |  |  |  |  |  |  |
| mmgC_4 |  | p |  |  |  |  |  |  |  |  |  |  |  |  |  |  |  |  |
| uctC_7 |  | p |  |  |  |  |  |  |  |  |  |  |  |  |  |  |  |  |
| acdA_2 |  |  |  |  |  |  |  |  |  |  |  |  |  |  |  |  |  |  |
| pka |  | P |  |  |  |  |  |  |  |  |  |  |  |  |  |  |  |  |
| Nitronate monooxygenase |  | P |  |  |  |  |  |  |  |  |  |  |  |  |  |  |  |  |
| Solute binding protein paaF_2 |  | p |  |  |  |  |  |  |  |  |  |  |  |  |  |  |  |  |
| 4- chlorobenzoyl coenzyme A dehalogenase |  | P |  |  |  |  |  |  |  |  |  |  |  |  |  |  |  |  |
| uctC_9 |  | p |  |  |  |  |  |  | P |  |  |  |  |  |  |  |  |  |
| fabG_4 | 1300-1350 | p |  |  |  |  |  |  |  |  |  |  |  |  |  |  |  |  |
| Putative acyl _coA dehydrogenase fadE25 |  | p |  |  |  |  |  |  |  |  |  |  |  |  |  |  |  |  |
| 3- hydroxybutyryl-coA dehydrogenase |  | p |  |  |  |  |  |  |  |  |  |  |  |  |  |  |  |  |
| acp |  | P |  |  |  |  |  |  |  |  |  |  |  |  |  |  |  |  |
| naiP_1 |  | P |  |  |  |  |  |  |  |  |  |  |  |  |  |  |  |  |
| Fliy_1 |  | P |  |  |  |  |  |  |  |  |  |  |  |  |  |  |  |  |
| xerC_4 | 1350-1400 | P |  |  |  |  |  |  |  |  |  |  |  |  |  |  |  |  |
| xerC_5 |  | p |  |  |  |  |  |  |  |  |  |  |  |  |  |  |  |  |
| xerD_2 |  | P |  |  |  |  |  |  |  |  |  |  |  |  |  |  |  |  |
| hemA_1 |  | p |  |  |  |  |  |  |  |  |  |  |  |  |  |  |  |  |
| asnO |  | P |  |  |  |  |  |  |  |  |  |  |  |  |  |  |  |  |
| pbuE_1 |  | P |  |  |  |  |  |  |  |  |  |  |  |  |  |  |  |  |
| alaS_1 | 1400-1450 | P |  |  |  |  |  |  |  |  |  |  |  |  |  |  |  |  |
| dmIR_8 |  | p |  |  |  |  |  |  |  |  |  |  |  |  |  |  |  |  |
| dosP |  | P |  |  |  |  |  |  |  |  |  |  |  |  |  |  |  |  |
| cbdA | 1450-1500 | P |  |  |  |  |  |  |  |  |  |  |  |  |  |  |  |  |
| catA |  | P |  |  |  |  |  |  |  |  |  |  |  |  |  |  |  |  |
| catB_1 |  | P |  |  |  |  |  |  |  |  |  |  |  |  |  |  |  |  |
| Low molecular weight cobalt containing nitrile hydratase subunit alpha |  | P |  |  |  |  |  |  |  |  |  |  |  |  |  |  |  |  |
| aer_3 |  | P |  |  |  |  |  |  |  |  |  |  |  |  |  |  |  |  |
| nagX |  | P |  |  |  |  |  |  |  |  |  |  |  |  |  |  |  |  |
| topB_5 | 1500-1550 | p | p |  |  |  | p |  |  |  |  | p |  |  |  | p |  |  |
| xerC_7 |  | P |  |  |  |  |  |  |  |  |  |  |  |  |  |  |  |  |
| IS3 family transposase ISBxe1 xerC_8 |  | p |  |  |  |  |  |  |  |  |  |  |  |  |  |  |  |  |
| IS3 family transposase ISBxe1 |  | P |  |  |  |  |  |  |  |  |  |  |  |  |  |  |  |  |
| ItrA_1 |  | p |  |  |  |  |  |  |  |  |  |  |  |  |  |  |  |  |
| IS3 family transposase ISBxe1 |  | P |  |  |  |  |  |  |  |  |  |  |  |  |  |  |  |  |
| xerD_3 |  | P |  |  |  |  |  |  |  |  |  |  |  |  |  |  |  |  |
| xerC_9 |  | P |  |  |  |  |  |  |  |  |  |  |  |  |  |  |  |  |
| XerC_10 |  | P |  |  |  |  |  |  |  |  |  |  |  |  |  |  |  |  |
| bacC_2 | 1550-1600 | P |  |  |  |  |  |  |  |  |  |  |  |  |  |  |  |  |
| bphE |  | P |  |  |  |  |  |  |  |  |  |  |  |  |  |  |  |  |
| IS91 family transposase ISPps1 |  | P |  |  |  |  |  |  |  |  |  |  |  |  |  |  |  |  |
| ycil |  | P |  |  |  |  |  |  |  |  |  |  |  |  |  |  |  |  |
| IS91 family transpose ISPps1 |  | P |  |  |  |  |  |  |  |  |  |  |  |  |  |  |  |  |
| IS1595 family transposase ISAisp1 |  | P |  |  |  |  |  |  |  |  |  |  |  |  |  |  |  |  |
| pcaK_3 |  | P |  |  |  |  |  |  |  |  |  |  |  |  |  |  |  |  |
| rclc | 1950-2000 | P |  |  |  |  |  |  |  |  |  |  |  |  |  |  |  |  |
| cdhR_1 | 2150-2200 | P |  |  |  |  |  |  |  |  |  |  |  |  |  |  |  |  |
| dmIR_10 |  | P |  |  |  |  |  |  |  |  |  |  |  |  |  |  |  |  |
| qorA_1 |  | P |  |  |  |  |  |  |  |  |  |  |  |  |  |  |  |  |
| pcpR_1 |  | P |  |  |  |  |  |  |  |  |  |  |  |  |  |  |  |  |
| kefF |  | P |  |  |  |  |  |  |  |  |  |  |  |  |  |  |  |  |
| IS3 family transposase ISBxe |  | P |  |  |  |  |  |  |  |  |  |  |  |  |  |  |  |  |
| IS3 family transposase ISBxe1 | 2200-2250 | P |  |  |  |  |  |  |  |  |  |  |  |  |  |  |  |  |
| ioLS_1 | 2250-2300 | P |  |  |  |  |  |  |  |  |  |  |  |  |  |  |  |  |
| thlA_2 |  | P |  |  |  |  |  |  |  |  |  |  |  |  |  |  |  |  |
| bcr_1 |  | P |  |  |  |  |  |  |  |  |  |  |  |  |  |  |  |  |
| gcvA_6 |  | P |  |  |  |  |  |  |  |  |  |  |  |  |  |  |  |  |
| virB4_1 |  | p | p | p |  |  | p |  |  |  |  |  |  |  |  |  | p |  |
| rhaR_1 | 2300-2350 | p |  |  | P |  |  |  |  |  |  |  |  |  |  |  |  |  |
| qacA |  | P |  |  |  |  |  |  |  |  |  |  |  |  |  |  |  |  |
| pseB | 2350-2400 | P |  |  |  |  |  |  |  |  |  |  |  |  |  |  |  |  |
| pseC |  | p |  | p |  |  |  |  |  |  |  |  |  | P |  |  |  |  |
| neuA |  | p |  | p |  |  |  |  |  |  |  |  |  | p |  |  |  |  |
| pseG |  | p |  | p |  |  |  |  |  |  |  |  |  | P |  |  |  |  |
| psel |  | p |  | p |  |  |  |  |  |  |  |  |  | P |  |  |  |  |
| ssuE |  | p |  |  | P |  |  |  |  |  |  |  |  |  |  |  |  |  |
| ssuA | 2500-2550 | P |  |  | P |  |  |  |  |  |  |  |  |  |  |  |  |  |
| msuD |  | P |  |  | P |  |  |  |  |  |  |  |  |  |  |  |  |  |
| acyll |  | p |  | p | p | p | p | P |  |  |  |  |  |  |  |  |  |  |
| menH_2 |  | p | p | p |  | P |  |  |  |  |  |  |  |  |  |  |  |  |
| arcD_1 |  | p | p | p |  | P |  |  |  |  |  |  |  |  |  |  |  |  |
| areA |  | p | p | p |  | P |  |  |  |  |  |  |  |  |  |  |  |  |
| arcD_2 |  | p | p | p |  | P |  |  |  |  |  |  |  |  |  |  |  |  |
| intA_1 | 3000-3050 | P |  |  |  |  |  |  |  |  |  |  |  |  |  |  |  |  |
| gabR_3 | 3100-3150 |  |  |  |  |  |  | p | P |  |  |  |  |  |  |  |  |  |
| hdfR_3 | 3150-3200 | p | p | P |  |  |  |  |  |  |  |  |  |  |  |  |  |  |
| cysJ |  | p | p | P |  |  |  |  |  |  |  |  |  |  |  |  |  |  |
| cysI |  | p | p | P |  |  |  |  |  |  |  |  |  |  |  |  |  |  |
| hemN_2 | 3200-3250 | p | p | p | p | P |  |  |  |  |  |  |  |  |  |  |  |  |
| nrdD |  | p | p | p | p | P |  |  |  |  |  |  |  |  |  |  |  |  |
| yhbU |  | p | p | p | p | P |  |  |  |  |  |  |  |  |  |  |  |  |
| fpvA |  |  |  |  |  |  |  |  |  |  |  |  | P | p |  |  |  |  |
| Leu/Ile/Val binding protein | 3450-3500 | p |  | p |  |  |  | p | P |  |  |  |  |  |  |  |  |  |
| IivH_10 |  | p |  | p |  |  |  | p | P |  |  |  |  |  |  |  |  |  |
| box A | 3750-3800 | p |  | p | p | p | p | p | p |  |  |  | p |  |  | p | P |  |
| box B |  | p |  | p | p | p | p | p | p |  |  |  | p |  |  | p | P |  |
| box C |  | p |  | p | p | p | p | p | p |  |  |  | p |  |  | p | P |  |
| aroK_1 |  | p |  | p | p | p | p | p | p |  |  |  | p |  |  | p | P |  |
| box D |  | p |  | p | p | p | p | p | p |  |  |  | p |  |  | p | P |  |
| napA_1 | 3800-3850 | p | p | p | p | p | p | p | p | P |  |  |  |  |  |  |  |  |
| nasD |  | p | p | p | p | p | p | p | p | p |  |  |  |  |  |  |  |  |
| ptlB |  | p | P |  |  |  |  |  |  |  |  |  |  |  |  |  |  |  |
| virB4_2 |  | p | P |  |  |  |  |  |  |  |  |  |  |  |  |  |  |  |
| virB5 | 3900-3950 | p | P |  |  |  |  |  |  |  |  |  |  |  |  |  |  |  |
| virB6 |  | p | P |  |  |  |  |  |  |  |  |  |  |  |  |  |  |  |
| virB8 |  | p | P |  |  |  |  |  |  |  |  |  |  |  |  |  |  |  |
| virB9 |  | p | p |  |  |  |  |  |  |  |  |  |  |  |  |  |  |  |
| Tn3 family transposase ISPa43 |  | P |  |  |  |  |  |  |  |  |  |  |  |  |  |  |  |  |
| xylF_1 |  | P |  |  |  |  |  |  |  |  |  |  |  |  |  |  |  |  |
| bnzB |  | P |  |  |  |  |  |  |  |  |  |  |  |  |  |  |  |  |
| todD | 3950-4000 | P |  |  |  |  |  |  |  |  |  |  |  |  |  |  |  |  |
| andAd_2 |  | p |  |  |  |  |  |  |  |  |  |  |  |  |  |  |  |  |
| nanT_2 |  | P |  |  |  |  |  |  |  |  |  |  |  |  |  |  |  |  |
| tfdF_3 |  | p |  |  |  |  |  |  |  |  |  |  |  |  |  |  |  |  |
| IS66 family transposase ISPa82 |  | P |  |  |  |  |  |  |  |  |  |  |  |  |  |  |  |  |
| Tn3 family transposase IS1071 |  | P |  |  |  |  |  |  |  |  |  |  |  |  |  |  |  |  |
| IS3 family transposase ISBxe1 |  | P |  |  |  |  |  |  |  |  |  |  |  |  |  |  |  |  |
| kdgR_4 |  | P |  |  |  |  |  |  |  |  |  |  |  |  |  |  |  |  |
| paaz_3 |  | P |  |  |  |  |  |  |  |  |  |  |  |  |  |  |  |  |
| Pat_4 |  | P |  |  |  |  |  |  |  |  |  |  |  |  |  |  |  |  |
| uctC_15 |  | P |  |  |  |  |  |  |  |  |  |  |  |  |  |  |  |  |
| ghrB_2 | 4000-4050 |  |  |  |  |  |  | p | P |  |  |  |  |  |  |  |  |  |
| rbsR |  |  |  |  |  |  |  | p | P |  |  |  |  |  |  |  |  |  |
| Solute binding protein |  |  |  |  |  |  |  | p | P |  |  |  |  |  |  |  |  |  |
| nimT | 4050-4100 | p |  |  | P |  |  |  |  |  |  |  |  |  |  |  |  |  |
| tmoT_3 | 4100-4150 | p | P |  |  |  |  |  |  |  |  |  |  |  |  |  |  |  |
| NADH dehydrogenase like protein |  | p | P |  |  |  |  |  |  |  |  |  |  |  |  |  |  |  |
| sasA_8 |  | p | P |  |  |  |  |  |  |  |  |  |  |  |  |  |  |  |
| yheG putative multidrug efflux transpoter | 4200-4250 | p |  |  |  |  |  |  |  |  |  |  |  |  |  |  |  |  |
| norB | 4250-4300 | p | p | p | p |  |  |  |  |  |  |  |  |  |  |  |  |  |
| qoxC |  | p | p | p | p |  |  |  |  |  |  |  |  |  |  |  |  |  |
| nirQ |  | p | p | p | p |  |  |  |  |  |  |  |  |  |  |  |  |  |
| nirS_1 |  | p | p | p | p |  |  |  |  |  |  |  |  |  |  |  |  |  |
| nirT |  | p | p | p | p |  |  |  |  |  |  |  |  |  |  |  |  |  |
| nirB |  | p | p | p | p |  |  |  |  |  |  |  |  |  |  |  |  |  |
| mftC |  | p | p | p | p |  |  |  |  |  |  |  |  |  |  |  |  |  |
| eysG |  | p | p | p | p |  |  |  |  |  |  |  |  |  |  |  |  |  |
| nirS |  | p | p | p | p |  |  |  |  |  |  |  |  |  |  |  |  |  |
| ccmH | 4300-4350 | p | p | p | p |  |  |  |  |  |  |  |  |  |  |  |  |  |
| napG |  | p | p | p | p |  |  |  |  | p | p |  |  |  |  |  |  |  |
| napH |  | p | p | p | p |  |  |  |  | p | p |  |  |  |  |  |  |  |
| zupT |  | p | p | p | p | p |  |  |  |  |  |  |  |  |  |  |  |  |
| dmsA | 4400-4450 | p |  |  |  |  |  |  |  |  |  |  |  |  |  |  |  |  |
| rupA_ |  | p |  |  |  |  |  |  |  |  |  |  |  |  |  |  |  |  |
| ttgR |  | P |  |  |  |  |  |  |  |  |  |  |  |  |  |  |  |  |
| ispB | 4450-4500 | p |  |  |  |  |  |  |  |  |  |  |  |  |  |  |  |  |
| ber-4 |  | p |  |  |  |  |  |  |  |  |  |  |  |  |  |  |  |  |
| rhaS_6 |  | P |  |  |  |  |  |  |  |  |  |  |  |  |  |  |  |  |
| IS3 family transposase ISAs20 |  | p | P |  |  |  |  |  |  |  |  |  |  |  |  |  |  |  |
| recD | 4500-4550 | P |  |  |  |  |  |  |  |  |  |  |  |  |  |  |  |  |
| yhaV_2 |  | p | p |  |  |  | p |  |  |  |  | p |  |  |  | P |  |  |
| rep_2 |  | P |  |  |  |  |  |  |  |  |  |  |  |  |  |  |  |  |
| ItrA_2 |  | p |  |  |  |  |  |  |  |  |  |  |  |  |  |  |  |  |
| IS3 family tranposase ISBxe1 |  | P |  |  |  |  |  |  |  |  |  |  |  |  |  |  |  |  |
| topB_6 | 4550-4600 | p | p |  |  |  | p |  |  |  |  | p |  |  |  | P |  |  |
| Putative protein | 4550-4600 | p | p |  |  |  | p |  |  |  |  | p |  |  |  | P |  |  |
| acs_2 | 4600-4650 | p |  |  |  |  |  | p | P |  |  |  |  |  |  |  |  |  |
| dpnA | 4650-4700 | P |  |  |  |  |  |  |  |  |  |  |  |  |  |  |  |  |
| kmo |  | p |  |  |  |  |  | p | P |  |  |  |  |  |  |  |  |  |
| fabF_3 |  | p |  |  |  |  |  | p | P |  |  |  |  |  |  |  |  |  |
| cusB | 4800-4850 | p | p | P |  |  |  |  |  |  |  |  |  |  |  |  |  |  |
| cusA |  | p | p | P |  |  |  |  |  |  |  |  |  |  |  |  |  |  |
| dsbD_1 |  | p | p | P |  |  |  |  |  |  |  |  |  |  |  |  |  |  |
| cnrA | 4850-4900 | p | p | P |  |  |  |  |  |  |  |  |  |  |  |  |  |  |
| hcaR_8 |  | p | p | P |  |  |  |  |  |  |  |  |  |  |  |  |  |  |
| gcd | 4900-4950 | p | p | p | P |  |  |  |  |  |  |  |  |  |  |  |  |  |
| iolG | 5050-5100 | p |  | P |  |  |  |  |  |  |  |  |  |  |  |  |  |  |
| wbpl_2 |  | p |  | P |  |  |  |  |  |  |  |  |  |  |  |  |  |  |
| mshA_7 |  | p |  | P |  |  |  |  |  |  |  |  |  |  |  |  |  |  |
| pglK |  | p |  | P |  |  |  |  |  |  |  |  |  |  |  |  |  |  |
| narK | 5250 | p | p | p | p | p |  |  |  |  |  |  |  | P |  |  |  |  |
| narG_2 |  | p | p | p | p | p |  |  |  |  |  |  |  | P |  |  |  |  |
| narH |  | p | p | p | p | p |  |  |  |  |  |  |  | P |  |  |  |  |
| narJ |  | p | p | p | p | p |  |  |  |  |  |  |  | P |  |  |  |  |
| surA_3 |  | p | p | p | p | p |  |  |  |  |  |  |  | P |  |  |  |  |
| degU |  | p | p | p | p | p |  |  |  |  |  |  |  | P |  |  |  |  |

[P= Present]
